# A Sequential Adaptive Intervention Strategy Targeting Remission and Functional Recovery in Young People at Ultrahigh Risk of Psychosis: The Staged Treatment in Early Psychosis (STEP) Sequential Multiple Assignment Randomized Trial

**DOI:** 10.1001/jamapsychiatry.2023.1947

**Published:** 2023-06-28

**Authors:** Patrick D. McGorry, Cristina Mei, G. Paul Amminger, Hok Pan Yuen, Melissa Kerr, Jessica Spark, Nicky Wallis, Andrea Polari, Shelley Baird, Kate Buccilli, Sarah-Jane A. Dempsey, Natalie Ferguson, Melanie Formica, Marija Krcmar, Amelia L. Quinn, Yohannes Mebrahtu, Arlan Ruslins, Rebekah Street, Cassandra Wannan, Lisa Dixon, Cameron Carter, Rachel Loewy, Tara A. Niendam, Martha Shumway, Barnaby Nelson

**Affiliations:** 1 Orygen, Melbourne, Victoria, Australia; 2 Centre for Youth Mental Health, The University of Melbourne, Melbourne, Victoria, Australia; 3 Orygen Specialist Program, Melbourne, Victoria, Australia; 4 Department of Psychiatry, Columbia University, New York, New York; 5 Department of Psychiatry and Behavioral Sciences, University of California, Davis, Sacramento; 6 Department of Psychiatry and Behavioral Sciences, University of California, San Francisco

## Abstract

**Importance:**

Clinical trials have not established the optimal type, sequence, and duration of interventions for people at ultrahigh risk of psychosis.

**Objective:**

To determine the effectiveness of a sequential and adaptive intervention strategy for individuals at ultrahigh risk of psychosis.

**Design, Setting, and Participants:**

The Staged Treatment in Early Psychosis (STEP) sequential multiple assignment randomized trial took place within the clinical program at Orygen, Melbourne, Australia. Individuals aged 12 to 25 years who were seeking treatment and met criteria for ultrahigh risk of psychosis according to the Comprehensive Assessment of At-Risk Mental States were recruited between April 2016 and January 2019. Of 1343 individuals considered, 342 were recruited.

**Interventions:**

Step 1: 6 weeks of support and problem solving (SPS); step 2: 20 weeks of cognitive-behavioral case management (CBCM) vs SPS; and step 3: 26 weeks of CBCM with fluoxetine vs CBCM with placebo with an embedded fast-fail option of ω-3 fatty acids or low-dose antipsychotic medication. Individuals who did not remit progressed through these steps; those who remitted received SPS or monitoring for up to 12 months.

**Main Outcomes and Measures:**

Global Functioning: Social and Role scales (primary outcome), Brief Psychiatric Rating Scale, Scale for the Assessment of Negative Symptoms, Montgomery-Åsberg Depression Rating Scale, quality of life, transition to psychosis, and remission and relapse rates.

**Results:**

The sample comprised 342 participants (198 female; mean [SD] age, 17.7 [3.1] years). Remission rates, reflecting sustained symptomatic and functional improvement, were 8.5%, 10.3%, and 11.4% at steps 1, 2, and 3, respectively. A total of 27.2% met remission criteria at any step. Relapse rates among those who remitted did not significantly differ between SPS and monitoring (step 1: 65.1% vs 58.3%; step 2: 37.7% vs 47.5%). There was no significant difference in functioning, symptoms, and transition rates between SPS and CBCM and between CBCM with fluoxetine and CBCM with placebo. Twelve-month transition rates to psychosis were 13.5% (entire sample), 3.3% (those who ever remitted), and 17.4% (those with no remission).

**Conclusions and Relevance:**

In this sequential multiple assignment randomized trial, transition rates to psychosis were moderate, and remission rates were lower than expected, partly reflecting the ambitious criteria set and challenges with real-world treatment fidelity and adherence. While all groups showed mild to moderate functional and symptomatic improvement, this was typically short of remission. While further adaptive trials that address these challenges are needed, findings confirm substantial and sustained morbidity and reveal relatively poor responsiveness to existing treatments.

**Trial Registration:**

ClinicalTrials.gov Identifier: NCT02751632

## Key Points

**Question:**

What are the optimal type, timing, and sequence of interventions for individuals at ultrahigh risk of psychosis?

**Findings:**

In this sequential multiple assignment randomized trial including 342 individuals, a specialized psychological intervention (cognitive-behavioral case management [CBCM]) and a psychopharmacological intervention (CBCM and antidepressant medication) were not more efficacious than control conditions in improving remission and functional recovery. Relapse rates among individuals who remitted were high.

**Meaning:**

The findings of this study show that addition of sequentially more specialized psychosocial and antidepressant treatment for individuals who did not remit did not lead to superior outcomes, underscoring the need for further adaptive trials, treatment innovation, and an extended duration of care for relapse prevention.

## Teaser

This sequential multiple assignment randomized trial evaluates the efficacy of an intervention strategy for individuals at ultrahigh risk of psychosis.

## Introduction

First-episode psychosis is typically preceded by a prodrome characterized by nonspecific and attenuated psychotic symptoms and functional impairment. Ultrahigh risk criteria were introduced to prospectively identify this prodromal period.^1^ Despite more than 50 ultrahigh risk treatment studies,^2^ including a number of high-quality randomized clinical trials, treatment innovation is urgently needed.^3,4^ While pairwise meta-analytic evidence supports the efficacy of cognitive-behavioral interventions in reducing transition to psychosis,^4,5^ network meta-analyses indicate a lack of evidence for the superiority of any 1 type of intervention.^5,6^ However, group-level improvement has been seen in experimental and active control conditions,^7,8^ indicating that a range of treatments impact transition. Improvements in other symptomatic and functional domains have not been established,^4,9,10,11,12,13^ supporting the need to broaden primary treatment outcomes beyond transition and prioritize functional recovery.^4,14,15^

Unraveling the heterogeneity in the ultrahigh risk population is paramount to identify subgroups for whom treatments can be tailored.^16^ One potential solution is to develop adaptive treatment strategies that are delivered proactively and sequentially, consistent with clinical staging.^15^ Adaptive trial designs are dynamic and enable the treatment type or intensity to be individualized and adjusted in response to treatment progress, thus optimizing outcomes and real-world implementation.^17^ This approach also enhances sample enrichment by addressing issues in the ultrahigh risk field, notably low transition to psychosis rates.^18,19^ That is, individuals who do not respond to simple initial interventions may constitute a subgroup at increased risk of poor clinical outcome.^15,20^

The current study (Staged Treatment in Early Psychosis [STEP]) aimed to evaluate the outcomes of a sequential multiple assignment randomized clinical trial (SMART) comprising support and problem solving (SPS), cognitive-behavioral case management (CBCM), and fluoxetine for individuals at ultrahigh risk for psychosis.^15^ These treatments and their sequence were selected due to their potential benefits and safety profile.^15,21,22^ The sequential treatment strategy, which examined the efficacy of escalating the intensity and duration of treatment for individuals who did not remit on a randomized basis,^15^ was expected to produce superior outcomes over 12 months than single-phase trials. Hypotheses were as follows:

Hypothesis 1. Open-label SPS would produce a remission rate of 50% (step 1), based on modeling using previous randomized clinical trial data.^8^
Hypothesis 2a. CBCM would result in significantly better functional outcome than SPS (step 2).
Hypothesis 2b. In the CBCM group, baseline cognitive biases or vulnerabilities would predict remission and clinical outcome (including functioning) and
Hypothesis 2c. In the CBCM group, participants with better outcomes and those who remitted would show significantly greater change on these variables than those with worse outcomes and those who did not remit.
Hypothesis 3. Antidepressant medication (when added to background CBCM) would result in significantly better clinical outcomes than placebo (step 3).
Hypothesis 4. In those who remitted to step 1 and 2 treatments (SPS and CBCM), a maintenance treatment of SPS would result in a significantly lower rate of relapse than monitoring.

## Methods

### Study Design and Setting

The SMART design comprised 3 steps: open-label SPS (6 weeks), SPS vs CBCM (20 weeks), and CBCM with fluoxetine vs CBCM with placebo (26 weeks) (eFigure 1 in Supplement 1). Assessments occurred at baseline and weeks 4, 6 (step 1), 12, 24 (step 2), 36, and 52 (step 3) (see Supplement 1 for the schedule of assessments). The trial was conducted at the Personal Assessment and Crisis Evaluation (PACE) clinic at Orygen, a youth mental health service and research center,^23^ and at Orygen’s 4 headspace centers (youth-friendly enhanced primary care centers).^24^ Enrollment occurred between April 2016 and January 2019. The study was approved by the Melbourne Health Human Research Ethics Committee. The study protocol^15^ is available in Supplement 1. Baseline data have been reported previously.^20^ The study followed the Consolidated Standards of Reporting Trials (CONSORT) reporting guideline.

### Participants

Individuals seeking treatment were eligible for trial inclusion if they were aged 12 to 25 years, met ultrahigh risk criteria,^25^ spoke adequate English to understand the information and consent form and the questions involved in the clinical instruments, and were able to provide informed consent (including parental or guardian consent for those younger than 18 years). Exclusion criteria were previous psychotic episode of 1 week or longer; attenuated psychotic symptoms only present during acute intoxication; organic brain disease known to cause psychotic symptoms; metabolic, endocrine, or other physical illness; serious developmental disorder; and history of developmental delay or intellectual disability.^20^ Participants taking antidepressant or antipsychotic medication were excluded if medication could not be discontinued at study entry.

Participants were screened using a standardized clinical assessment and the Prodromal Questionnaire-16 (PQ-16).^26^ For those who scored 6 or higher positive symptoms, had a family history of psychotic disorder, or for whom there was a clinical impression of possibly meeting ultrahigh risk criteria, ultrahigh risk status was determined using the Comprehensive Assessment of At-Risk Mental States (CAARMS),^25^ Social and Occupational Functioning Assessment Scale (SOFAS),^27^ SCID-II Schizotypal PD, and Family History Index.^20^

### Randomization

Randomization was based on computer-generated treatment allocations prepared by independent personnel. Participants who met remission criteria at the end of step 1 were randomized to SPS or monitoring; those who did not remit were randomized to CBCM or SPS. Assessors were blind to treatment allocation. Participants in the nonremission arm of the trial were randomized a second time based on remission status at the end of step 2. Those who remitted were randomized to SPS or monitoring; those who did not were randomized to CBCM with fluoxetine or CBCM with placebo, stratified by Montgomery-Åsberg Depression Rating Scale (MADRS) total score (lower than 21 vs 21 or higher). In step 3, assessors and participants were blind to treatment allocation.

### Treatments

#### Monitoring

Clinicians monitored participants’ mental state and risk at 3, 6, 9, and 12 months postentry to step 1. Any deterioration in mental health was appropriately managed.

#### SPS

Manualized supportive counseling and problem-solving strategies were delivered within a positive psychology framework. Sessions were 30 to 50 minutes (weekly to fortnightly). In the relapse prevention and remission trial arm, participants received monthly SPS for up to 12 months.

#### CBCM

CBCM, a specialized manualized psychosocial intervention, consisted of cognitive behavioral therapy (CBT) provided within a case management framework.^28^ CBT included psychoeducation, behavioral strategies or experiments, and cognitive restructuring targeting attenuated psychotic symptoms, stress management, negative symptoms or depression, and other comorbidities. Case management support addressed current practical and social issues. Sessions were 30 to 50 minutes (weekly or as needed). See eMethods 1 in Supplement 2 for further details on SPS and CBCM.

#### Medications

Step 3 comprised 1 capsule of fluoxetine, 20 mg per day, or placebo, increasing to 40 mg per day or 2 placebo capsules after 6 weeks if clinical response was insufficient as judged by the treating psychiatrist. At 12 weeks, a fast-fail option was available for participants who had deteriorated or had not responded to treatment. Through a shared decision-making process, participants chose to either continue as randomized, increase the fluoxetine or placebo dosage, or receive an add-on of low-dose antipsychotic medication (quetiapine, 50-300 mg per day, or aripiprazole, 10-30 mg per day) or long-chain ω-3 fatty acids (2.8 g per day of marine fish oil in 4 × 0.7-g capsules containing approximately 1.4 g eicosapentanoic and docosahexanoic acid).

### Outcome Measures

For power purposes and sample size calculation,^15^ the primary outcome chosen was functioning at 6 months (end of step 2), measured by the Global Functioning: Social and Role scales.^29^ However, an important perspective for some research considerations is the pattern of remission across all steps over 12 months. Remission criteria, reflecting symptomatic and functional remission, were a global rating scale or frequency score less than 3 on the CAARMS unusual thought content, nonbizarre ideas, and perceptual abnormalities subscales; a global rating scale score less than 4 or frequency score less than 3 on the disorganized speech subscale; and improvement of 5 or more points on the SOFAS compared with baseline or a SOFAS score of 70 or higher. Remission rates reported reflect sustained remission at the end of a step; that is, remission criteria were met at weeks 4 and 6 for step 1, 12 and 24 for step 2, and 36 and 52 for step 3. Relapse was recorded if remission criteria were no longer met at 6 or 12 months.

Other outcome measures included the Brief Psychiatric Rating Scale (BPRS);^30^ Scale for the Assessment of Negative Symptoms (SANS);^31^ MADRS;^32^ Assessment of Quality of Life (AQoL-8D); SOFAS; transition to psychosis, defined as daily positive psychotic symptoms for 1 week or longer (CAARMS); and Davos Assessment of Cognitive Biases Scale (DACOBS; validated self-report measure of cognitive problems and biases).^33^

Therapy sessions were audio recorded with consent and independently rated for fidelity. Based on the minimum number of sessions provided for each treatment at each step,^15^ adherence in the nonremission arm was defined as 3 or more sessions of SPS (step 1), 9 or more of SPS (step 2), and 6 or more of CBCM (steps 2 and 3). For the remission arm, the number of sessions was 5 or more for SPS and 2 or more for monitoring (for those who remitted at step 1), 3 or more for SPS, and 1 or more for monitoring (for those who remitted at step 2). Medication adherence was based on participants’ estimation of dose taken (<50%, 51%-75%, and >75%) and capsule count, with adherence achieved when both were greater than 75%. Adverse events were assessed at baseline and each visit.

### Statistical Analysis

The study was powered to detect a small to medium effect size (Cohen *f*) of 0.18 on the primary 6-month functional outcome (end of step 2) and a medium effect size of 0.29 at 12 months (end of step 3) (Nelson et al^15^ and Supplement 1). The intent-to-treat approach was used with missing data handled using multiple imputation (eMethods 2 in Supplement 2). Complete-case (ie, only including participants with nonmissing data) and per-protocol analyses (ie, only including participants who adhered to the allocated treatments) were also conducted. For continuous outcomes, a general linear model analysis compared the groups concerned with the relevant original baseline score (ie, day 1) as a covariate. χ^2^ test compared remission rates between treatments. Relapse rates were examined using the Fisher exact test for individuals who remitted at step 1 and logistic regression for those who remitted at step 2. Relapse at 12 months was the dependent variable, and group factors were SPS vs CBCM and SPS vs monitoring. The interaction term between these 2 factors was included. Transition to psychosis rates were examined using Kaplan-Meier estimation and log-rank test. The correlation between DACOBS and clinical measures and 6-month remission status was examined using Pearson correlation and point-biserial correlation, respectively. The *t* test compared change in DACOBS total score (6-month score minus baseline) between individuals who did and did not remit at step 2. Generalized linear mixed effects logistic regression estimated the probability of treatment fidelity with agreement between allocated treatment and rater-determined treatment as the dependent variable and therapy type (SPS or CBCM) as a fixed factor and participants and clinicians as random factors. Significance level was set at .05.

## Results

### Participant Flow

**Figure 1.**
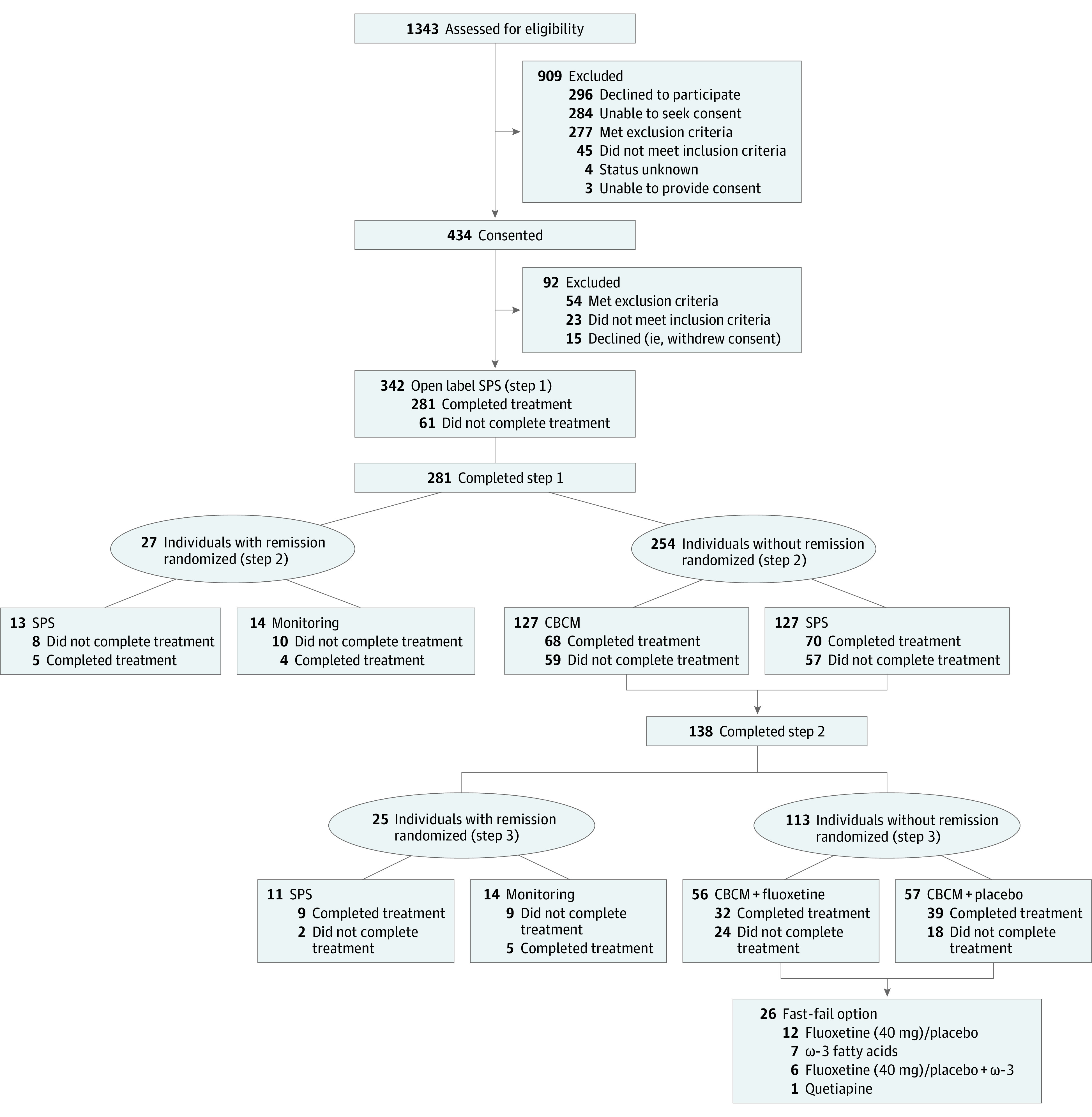
CONSORT Diagram

Of 1343 individuals considered, 342 were included (198 female; mean [SD] age, 17.7 [3.1] years) (Table 1). Assignment to each treatment arm according to remission status is shown in Figure 1. Participants (n = 342) and nonparticipants (n = 787, excluding those who did not meet ultrahigh risk criteria or represent the target population) did not significantly differ according to age and sex. The frequency of concomitant medication for the entire sample (n = 342) over the 24-month study period was 33.0% (n = 113) for antidepressants, 4.7% (n = 16) for antipsychotics, 6.7% (n = 23) for anxiolytics, 8.8% (n = 30) for sedatives, and 78.9% (n = 270) for other medications.

**Table 1.** Sample Demographic Characteristics at Baseline

	Total sample (N = 342)
Age, mean (SD; range), y	17.7 (3.1; 12.5-25.0)
Female, No. (%)	198 (57.9)
Male, No. (%)	144 (42.1)
Currently in education, No. (%)	
Yes	245 (71.6)
No	95 (27.8)
Missing	2 (0.6)
Highest level of education, No. (%)	
Primary school	2 (0.6)
Year 7-10	160 (46.8)
Year 11-12	101 (29.5)
TAFE	30 (8.8)
Undergraduate degree	33 (9.6)
Postgraduate degree	4 (1.2)
Other	10 (2.9)
Missing	2 (0.6)
Current employment, No. (%)	
Unemployed	218 (63.7)
Casual paid	70 (20.5)
Part-time paid	27 (7.9)
Full-time paid	12 (3.5)
Casual unpaid	8 (2.3)
Part-time unpaid	2 (0.6)
Missing	5 (1.5)
CAARMS severity score,^a^ mean (SD)	
Unusual thought content	2.6 (1.8)
Nonbizarre ideas	2.9 (1.7)
Perceptual abnormalities	3.1 (1.5)
Disorganized speech	1.7 (1.2)
CAARMS frequency score,^b^ mean (SD)	
Unusual thought content	2.4 (1.7)
Nonbizarre ideas	3.0 (1.7)
Perceptual abnormalities	2.6 (1.4)
Disorganized speech	2.6 (1.9)
CAARMS composite score,^c^ mean (SD)	
Attenuated positive psychotic severity score	34.6 (16.8)
Negative symptoms score (SANS total score), mean (SD)	18.4 (11.2)
General psychopathology (BPRS total score), mean (SD)	44.6 (8.7)
Depressive symptoms (MADRS total score), mean (SD)	23.5 (9.9)
Global Functioning: Social, mean (SD)	6.6 (1.3)
Global Functioning: Role, mean (SD)	6.3 (1.6)
Social and occupational functioning (SOFAS), mean (SD)	56.7 (11.6)
Recruitment site, No. (%)	
Headspace	310 (90.6)
PACE	32 (9.4)

Abbreviations: BPRS, Brief Psychiatric Rating Scale; CAARMS, Comprehensive Assessment of At-Risk Mental States; MADRS, Montgomery-Åsberg Depression Rating Scale; PACE, Personal Assessment and Crisis Evaluation; SANS, Scale for the Assessment of Negative Symptoms; SPS, support and problem solving; SOFAS, Social and Occupational Functioning Assessment Scale; TAFE, Technical and Further Education.

^a^ Severity score ranges from 0 (never or absent) to 6 (psychotic and severe).

^b^ Frequency score ranges from 0 (absent) to 6 (continuous).

^c^ Composite score is the sum of the product of severity and frequency of the 4 subscales (unusual thought content, nonbizarre ideas, perceptual abnormalities, and disorganized speech).

Discontinuation rates were 17.8% (61 of 342; step 1), 47.7% (134 of 281; step 2), and 38.4% (53 of 138; step 3). Reasons for discontinuation are listed in eTable 1 in Supplement 2. Of 76 participants lost to follow-up, 19 (25.0%) met remission criteria at the last available follow-up, 36 (47.4%) did not meet remission criteria, and 21 (27.6%) were undeterminable due to no follow-up. Among those who discontinued for other reasons (n = 172), 32 (18.6%) met remission criteria at the last assessment before treatment discontinuation, 117 (68.0%) did not meet criteria, and 23 (13.4%) were undeterminable. The following results are from the intent-to-treat analysis with multiple imputation.

#### Hypothesis 1: Remission Rates

Remission rates (ie, sustained remission) across treatment arms at the end of steps 1, 2, and 3 were 8.5%, 10.3%, and 11.4%, respectively. The overall remission rate (ie, sustained remission at the end of at least one step) was 27.2%. There were no significant differences in remission rates between treatments at the end of step 2 (9.8% [CBCM] vs 10.7% [SPS]; *P* = .84) and step 3 (10.6% [CBCM with fluoxetine] vs 12.2% [CBCM with placebo]; *P* = .67). Remission rates of those recruited at PACE vs headspace did not significantly differ at step 1 (4.0% vs 9.0%), 2 (4.6% vs 10.9%), or 3 (14.6% vs 11.1%).

#### Hypothesis 2a: Effect of CBCM on Functioning and Clinical Measures

For the primary outcome, intent-to-treat analysis revealed no significant difference in mean scores on the Global Functioning: Social and Role scales between SPS and CBCM at 6 months (Table 2 and eFigure 1 in Supplement 2). There was no significant difference between SPS and CBCM on any clinical measure (Table 2 and eFigures1, 2A, and 3A in Supplement 2).

**Table 2.** General Linear Model Analysis Comparing Cognitive-Behavioral Case Management (CBCM) and Support and Problem Solving (SPS) at 6 Months and CBCM With Fluoxetine and CBCM With Placebo at 12 Months^e^

	Treatment		Step 2 (mean No. = 159 SPS, 153 CBCM)^d^				Step 3 (mean No. = 139 fluoxetine; 141 placebo)^d^					
			Mean (SE)		Effect size	*P* value^a^	Treatment	Mean (SE)		Effect size	*P* value^b^	*P* value^c^
			Baseline	Month 6				Baseline	Month 12			
GF: social	SPS		6.6 (0.10)	6.7 (0.17)	−0.07	.63	CBCM + placebo	6.5 (0.13)	6.5 (0.27)	−0.03	.85	.83
	CBCM		6.5 (0.10)	6.5 (0.17)			CBCM + fluoxetine	6.6 (0.12)	6.4 (0.29)			
GF: role	SPS		6.1 (0.13)	6.5 (0.19)	−0.03	.85	CBCM + placebo	6.2 (0.15)	6.5 (0.29)	−0.01	.93	.90
	CBCM		6.4 (0.12)	6.6 (0.19)			CBCM + fluoxetine	6.3 (0.15)	6.5 (0.30)			
BPRS total score	SPS		45.7 (0.70)	42.9 (0.82)	−0.02	.90	CBCM + placebo	45.8 (0.81)	41.6 (1.24)	0.15	.37	.90
	CBCM		44.8 (0.67)	42.3 (0.82)			CBCM + fluoxetine	45.9 (0.84)	43.1 (1.27)			
SANS total score	SPS		18.7 (0.92)	17.2 (1.05)	0.06	.65	CBCM + placebo	19.3 (1.06)	16.6 (1.35)	0.08	.60	.93
	CBCM		18.8 (0.89)	17.8 (1.13)			CBCM + fluoxetine	19.0 (1.08)	17.4 (1.50)			
MADRS total score	SPS		23.5 (0.77)	20.3 (0.96)	−0.07	.58	CBCM + placebo	24.8 (0.91)	18.7 (1.39)	0.11	.52	.87
	CBCM		24.9 (0.76)	20.2 (0.95)			CBCM + fluoxetine	24.5 (0.93)	19.8 (1.41)			
SOFAS	SPS		56.3 (0.96)	60.1 (1.21)	−0.04	.75	CBCM + placebo	56.0 (1.10)	60.0 (1.70)	−0.03	.84	.75
	CBCM		56.7 (0.91)	59.8 (1.22)			CBCM + fluoxetine	56.2 (1.13)	59.6 (1.63)			
AQoL total score	SPS		54.1 (0.99)	58.2 (1.10)	0.07	.60	CBCM + placebo	52.6 (1.16)	61.4 (1.53)	−0.10	.54	.83
	CBCM		51.6 (0.96)	57.2 (1.17)			CBCM + fluoxetine	52.0 (1.22)	59.9 (1.60)			
DACOBS total score	SPS		171.3 (2.5)	163.3 (2.8)	−0.11	.43	CBCM + placebo	171.2 (3.1)	152.4 (4.3)	0.08	.61	.84
	CBCM		171.5 (2.6)	160.8 (3.1)			CBCM + fluoxetine	173.8 (3.1)	156.7 (4.1)			
**CAARMS, severity**												
UTC		SPS	2.8 (0.13)	2.1 (0.18)	−0.05	.68	CBCM + placebo	2.8 (0.17)	2.0 (0.24)	0.12	.42	.88
		CBCM	2.7 (0.15)	2.0 (0.19)			CBCM + fluoxetine	2.9 (0.17)	2.2 (0.24)			
NBI		SPS	3.0 (0.13)	2.5 (0.18)	−0.07	.62	CBCM + placebo	3.0 (0.16)	2.0 (0.25)	0.14	.39	.47
		CBCM	3.0 (0.13)	2.3 (0.18)			CBCM + fluoxetine	3.1 (0.16)	2.3 (0.24)			
PA		SPS	3.1 (0.13)	2.8 (0.18)	−0.10	.45	CBCM + placebo	3.3 (0.14)	2.6 (0.23)	0.01	.93	.58
		CBCM	3.3 (0.11)	2.7 (0.18)			CBCM + fluoxetine	3.2 (0.15)	2.7 (0.25)			
DS		SPS	1.7 (0.10)	1.7 (0.15)	0.04	.80	CBCM + placebo	1.7 (0.12)	2.0 (0.21)	0.02	.92	.99
		CBCM	1.7 (0.09)	1.8 (0.14)			CBCM + fluoxetine	1.7 (0.11)	2.0 (0.21)			
**CAARMS, frequency**												
UTC		SPS	2.7 (0.13)	2.0 (0.18)	0.01	.93	CBCM + placebo	2.5 (0.16)	2.1 (0.24)	0.03	.85	.64
		CBCM	2.3 (0.14)	1.9 (0.19)			CBCM + fluoxetine	2.6 (0.16)	2.1 (0.25)			
NBI		SPS	3.2 (0.13)	2.8 (0.18)	−0.02	.87	CBCM + placebo	3.1 (0.16)	2.4 (0.25)	0.04	.78	.59
		CBCM	3.0 (0.13)	2.7 (0.19)			CBCM + fluoxetine	3.3 (0.16)	2.5 (0.25)			
PA		SPS	2.4 (0.12)	2.5 (0.17)	−0.07	0.61	CBCM + placebo	2.7 (0.13)	2.4 (0.24)	0.07	.69	.95
		CBCM	2.8 (0.10)	2.5 (0.18)			CBCM + fluoxetine	2.6 (0.14)	2.5 (0.25)			
DS		SPS	2.7 (0.16)	2.8 (0.20)	−0.06	0.68	CBCM + placebo	2.7 (0.17)	2.8 (0.26)	−0.04	.81	.87
		CBCM	2.5 (0.15)	2.6 (0.19)			CBCM + fluoxetine	2.6 (0.18)	2.7 (0.26)			

Abbreviations: AQoL, Assessment of Quality of Life; BPRS, Brief Psychiatric Rating Scale; CAARMS, Comprehensive Assessment of At-Risk Mental States; DACOBS, Davos Assessment of Cognitive Biases Scale; DS, disorganized speech; GF, Global Functioning Scale; MADRS, Montgomery-Åsberg Depression Rating Scale; NBI, nonbizarre ideas; PA, perceptual abnormalities; SANS, Scale for the Assessment of Negative Symptoms; SOFAS, Social and Occupational Functioning Assessment Scale; UTC, unusual thought content.

^a^ Comparing SPS and CBCM with baseline score as a covariate.

^b^ Comparing CBCM + placebo and CBCM + fluoxetine with baseline score as a covariate.

^c^ Interaction between step 2 treatment (SPS/CBCM) and step 3 treatment (CBCM + placebo / CBCM + fluoxetine) with baseline score as a covariate.

^d^ The mean sample sizes for each group over the multiple imputations.

^e^ Missing data handled by multiple imputation.

#### Hypotheses 2b and 2c: Effect of Baseline Cognitive Biases or Vulnerabilities on Remission and Treatment Outcome

While this hypothesis was intended to be restricted to the CBCM group, CBCM did not produce superior outcomes or greater improvement on cognitive biases or vulnerabilities than SPS (Table 2; eFigure 1 in Supplement 2). Hence, the following analyses include both the CBCM and SPS groups.

The correlation between baseline DACOBS total and 6-month outcomes and between change in DACOBS total and change in outcomes among those who did not remit at step 1 is presented in eTable 2 in Supplement 2. Baseline DACOBS was significantly correlated with BPRS (*r*, 0.15), SANS (*r*, 0.26), AQoL (*r*, −0.27), and SOFAS (*r*, −0.18) at 6 months. The correlation between baseline DACOBS and 6-month remission status was nonsignificant (*r*, −0.11; *P* = .12). At 6 months, improvement in DACOBS showed a significant correlation with improvements in BPRS (*r*, 0.31), MADRS (*r*, 0.26), and AQoL (*r*, −0.38). Participants who remitted at the end of step 2 (n = 26) showed a larger nonsignificant mean improvement in DACOBS than those who did not (n = 148; −13.0 vs −7.8; *P* = .38).

#### Hypothesis 3: Effect of Antidepressant Medication on Clinical Outcome

**Figure 2.**
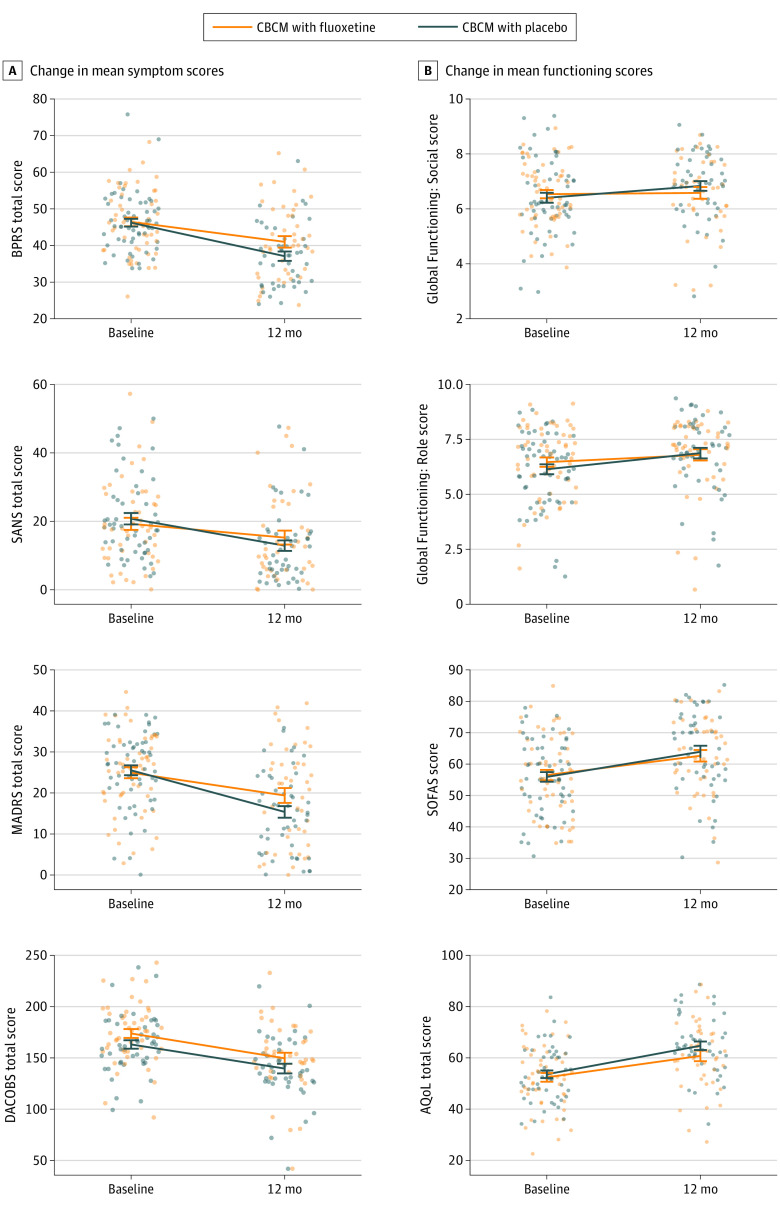
Changes in Mean Symptom and Functioning Scores From Baseline to the End of Step 3 Circles represent individual participant data. Fluoxetine or placebo commenced at 6 months (start of step 3). AQoL indicates assessment of quality of life; BPRS, Brief Psychiatric Rating Scale; CBCM, cognitive-behavioral case management; DACOBS, Davos Assessment of Cognitive Biases Scale; MADRS, Montgomery-Åsberg Depression Rating Scale; SANS, Scale for the Assessment of Negative Symptoms; SOFAS, Social and Occupational Functioning Assessment Scale.

Both treatment arms (CBCM with fluoxetine and CBCM with placebo) showed improvement by the end of step 3, with no significant difference on any measure (Table 2; Figure 2; eFigures 2B and 3B in Supplement 2). The interaction effect between MADRS score (<21 vs ≥21) and treatment (fluoxetine vs placebo) showed that the effect of fluoxetine did not significantly differ according to MADRS score on any measure, indicating that fluoxetine was not more effective for those with severe depression. Mean scores of the fast-fail group are shown in eTable 3 in Supplement 2.

#### Hypothesis 4: Relapse Rates and Outcomes of Participants Who Remitted

Six-month relapse rates were higher in the SPS group (59.8%) than monitoring (16.9%) among participants who remitted at step 1 (*P* = .03). This difference was not maintained at 12 months (65.1% vs 58.3%, respectively; *P* = .72). At the end of step 2, there was no significant difference in 12-month relapse rates (37.7% for SPS vs 47.5% for monitoring; *P* = .32).

Six-month SOFAS scores significantly differed between SPS and monitoring among participants who remitted at step 1 (effect size, 0.91; 95% CI, 0.95-19.6; *P* = .03) (eTable 4 in Supplement 2). The direction of the effect favored the monitoring group. At 12 months, there were no significant differences between the two groups on any measure among those who remitted at steps 1 or 2 (eTables 4-5 in Supplement 2).

### Transition to Psychosis

The Kaplan-Meier–estimated 12-month transition rate was 13.5% (95% CI, 9.1-18.0). The log-rank test indicated no significant difference between groups at 6 and 12 months (Table 3). The 12-month transition rate was 3.3% (95% CI, 0.3-7.0) for those who remitted at any stage and 17.4% (95% CI, 12.5-21.9) for those who did not (*P* = .001).

**Table 3.** Kaplan-Meier Estimated Transition Rates at 6 and 12 Months With Missing Data Handled by Multiple Imputation

		Mean No. over multiple imputations		Estimated transition rate, % (95% CI)	*P* value^a^
		Cases	Transitions		
6 mo					
Step 1 nonremission	SPS	159	11	6.9 (2.9-10.7)	.52
	CBCM	153	13	8.6 (4.1-13.0)	
12 mo					
Step 1 remission	Monitoring	15	1	3.3 (0-19.6)	.61
	SPS	14	1	4.2 (0-21.9)	
Step 2 nonremission	Placebo	141	22	15.3 (9.2-21.0)	.51
	Fluoxetine	139	23	16.1 (9.8-22.0)	
Step 2 remission	Monitoring	17	1	2.9 (0-18.3)	.66
	SPS	15	1	2.7 (0-20.1)	

Abbreviations: CBCM, cognitive-behavioral case management; SPS, support and problem solving.

^a^ *P* value comparing the survival curves of the treatments concerned using log-rank test.

### Overall Treatment Effect and Complete-Case and Per-Protocol Analyses

Across the entire sample, small to medium effect sizes were found at 6 and 12 months on measures of symptoms and functioning (eTable 6 in Supplement 2). The complete-case and per-protocol analyses yielded results consistent with intent-to-treat findings (eTables 7-15 in Supplement 2).

### Treatment Adherence and Fidelity

The mean (SD) number of sessions attended was 2.3 (1.5) and 5.7 (3.9) for SPS at steps 1 and 2, respectively, and 6.2 (3.7) at step 2 and 6.0 (4.2) at step 3 for CBCM. A total of 199 recordings (65 participants; 24 clinicians) were rated for fidelity, a mean (SD) of 3.1 (2.1) per participant and 8.3 (9.3) per clinician. The probability that each type of treatment was given as intended was estimated to be 0.95 (95% CI, 0.88-0.98) for SPS and 0.59 (95% CI, 0.51-0.67) for CBCM, indicating higher fidelity to SPS (*P* < .001). Adherence was low to moderate for SPS, moderate for CBCM, and low for fluoxetine and placebo (eTable 16 in Supplement 2). While adherence with SPS in participants in the step 2 nonremission arm was significantly lower than with CBCM (33 of 127 [26.0%] vs 68 of 127 [53.5%]), when adherence with both treatments was defined similarly (6 or more sessions), adherence with SPS increased (61 of 127 [48.0%]) and did not significantly differ from CBCM. When the per-protocol analyses were repeated with a 6 or more session adherence level for SPS, the results were similar to those obtained using a 9 or more session cut point and the conclusions remained the same.

### Adverse Events

Few adverse and serious adverse events occurred. No significant group differences were observed (eTable 17 in Supplement 2).

## Discussion

This is the first SMART trial to be conducted in the ultrahigh risk population. The primary outcome of functioning at the end of step 2 was selected for sample size and power calculations; however, results should be considered as a whole, spanning all steps. For the primary outcome, there was no difference between CBCM and SPS. While the sample overall showed modest functional and symptomatic improvement over 12 months, remission rates were lower than expected, the transition rate was slightly higher than anticipated, and there was no significant difference between groups on the secondary outcomes. Even when remission was achieved, it was difficult to maintain, and continuing supportive therapy did not reduce relapse rates compared to monitoring. While the monitoring group showed greater improvement in 6-month SOFAS scores than SPS, this was likely due to 4 outliers in the SPS group whose 6-month SOFAS outcomes were worse than the other individuals who remitted at step 1. The findings suggest that enhancing the intensity of treatment with psychological interventions (CBCM) or antidepressant medication in real-world youth mental health services does not produce benefit over continuing simpler care for a longer period. However, some caution is needed given the following caveats.

First, the bar for remission was high, although appropriately so, requiring sustained symptomatic and functional improvement. If remission had been based on symptomatic improvement only, the overall remission rate would have increased to 41.2% (compared to 27.2%).

Second, the rate of treatment discontinuation was substantial (18% at step 1, 48% at step 2, and 38% at step 3). However, this rate was comparable to that of the seminal Sequenced Treatment Alternatives to Relieve Depression (STAR*D) trial,^34^ albeit with a different population focus.

Third, adherence to psychological therapy and medication and fidelity to CBCM were suboptimal. These challenges are common in psychotherapy trials.^35,36,37,38^ The very low adherence to fluoxetine (26.8%) led to insufficient power, though its timing (6 months postbaseline) may have been too delayed to protect against transition to psychosis.^22^ Despite the moderate fidelity to CBCM, findings indicate that the greater the change in cognitive biases or vulnerabilities, the greater the clinical improvement across both treatment groups, suggesting the psychological target of CBCM may represent more of an outcome rather than mediating variable. Compared to ultrahigh risk cohorts who received CBCM in specialized services,^7^ the current predominately primary care-based sample showed more modest improvements and a slightly higher transition rate. The therapist team in this study were not expert research therapists as in past studies but a large, diverse group of frontline primary care clinicians who may not have been as capable of fidelity to the therapeutic model. Further examination of the real-world quality of CBCM, particularly in primary care settings, is needed. Extending the involvement of young people with lived experience beyond the design phase throughout the study may have led to higher retention, adherence, and fidelity.

A positive note is that transition to psychosis was significantly lower among participants who remitted (3.3%) than those who did not (17.4%). While this may indicate that trials with transition as the primary outcome may enroll an enriched sample by filtering out those with early remission, relapse to ultrahigh risk positive status was common. Although all treatment arms showed a trend of symptomatic and functional improvement by the end of steps 2 and 3, improvement was typically modest, slow to emerge, and somewhat fragile. The lack of a no-treatment comparison group makes firm conclusions difficult.

While some caution is warranted, and needs-based care should continue to be provided to all patients at ultrahigh risk, the findings indicate the need for further sequential randomized trials examining existing treatments with improved adherence and fidelity and for the development of innovative, more effective treatments and enhanced modes of delivery. This could include virtual reality; neurofeedback; higher-fidelity CBT; individual placement and support; biotherapies (eg, cannabidiol,^39^ antiinflammatories, and pomaglumetad methionil^40^); neuroprotective agents, including low-dose lithium^41^; and reconsideration of low-dose antipsychotic medication for individuals who do not remit to psychosocial interventions. Attempts to develop new treatments are under way^42,43,44^; however, we also need to better understand the effective elements^45^ of existing treatments, particularly from a transdiagnostic perspective.^46,47,48^

### Strengths and Limitations

To our knowledge, this is the largest ultrahigh risk for psychosis trial to date, reflecting the utility of primary care youth mental health platforms, particularly when recruiting medication-naive participants. However, the treatment provided was insufficient for a large proportion of our sample. Despite high dropout rates, the achieved sample sizes (192 at step 2 and 92 at step 3) were similar to original expectations because of the higher than expected nonremission rates. The moderate fidelity to CBCM, the low adherence to medication, and high degree of treatment discontinuation and missing data may have limited the ability to test hypotheses and identify benefits of treatments.

## Conclusions

Enhancing the intensity of treatment with psychological interventions or medications was challenging to implement with fidelity and adherence in this largely primary care-based sample but nevertheless could not be demonstrated to produce any benefit over and above continuing a simpler form of care. Low remission and high relapse rates confirm the sustained vulnerability and substantial morbidity of the ultrahigh risk population and highlight the need to conduct further adaptive trials, develop new treatments, provide sustained specialist care, and identify subgroups for whom treatments can be tailored.

## Notes

Supplement 1. Trial protocol

Supplement 2. eMethods eTable 1. Reasons for treatment discontinuation (%) eTable 2. Pearson correlation between baseline DACOBS total and 6-month outcomes and between change in DACOBS total and change in outcomes (Step 1 non-remitters) eTable 3. 12-month outcomes of the fast-fail group eTable 4. General linear model analysis comparing SPS and monitoring (Step 1 remitters) at 6 and 12 months with data handled using multiple imputation eTable 5. General linear model analysis comparing SPS and monitoring (Step 2 remitters) at 12 months with data handled using multiple imputation eTable 6. Effect sizes (Cohen’s d) at 6 and 12 months across the entire sample eTable 7. Complete-case and per-protocol analysis comparing CBCM and SPS at 6 months eTable 8. Complete-case and per-protocol analysis comparing CBCM+fluoxetine and CBCM+placebo at 12 months eTable 9. Complete-case and per-protocol analysis of remission rates eTable 10. Complete-case and per-protocol analysis comparing relapse rates between SPS and monitoring (Step 1 remitters) at 6 and 12 months eTable 11. Complete-case and per-protocol analysis comparing relapse rates between SPS and monitoring (Step 2 remitters) at 12 months using logistic regression eTable 12. Complete-case and per-protocol analysis comparing SPS and monitoring (Step 1 remitters) at 6 months eTable 13. Complete-case and per-protocol analysis comparing SPS and monitoring (Step 1 remitters) at 12 months eTable 14. Complete-case and per-protocol analysis comparing SPS and monitoring (Step 2 remitters) at 12 months eTable 15. Kaplan-Meier estimated transition rates (complete-case and per-protocol analysis) eTable 16. Treatment adherence rates eTable 17. Adverse events eFigure 1. Changes in mean symptom and functioning scores from baseline to the end of Step 2 eFigure 2. Changes in mean CAARMS severity scores from baseline to the end of Step 2 (panel A) and Step 3 (panel B) eFigure 3. Changes in mean CAARMS frequency scores from baseline to the end of Step 2 (panel A) and Step 3 (panel B)

Supplement 3. Data sharing statement
