# Supplementary material for: A Sequential Adaptive Intervention Strategy Targeting Remission and Functional Recovery in Young People at Ultrahigh Risk of Psychosis: The Staged Treatment in Early Psychosis (STEP) Sequential Multiple Assignment Randomized Trial
Source: JAMA Psychiatry. 2023 Jun 28;80(9):875–85. doi: 10.1001/jamapsychiatry.2023.1947 (PMC10308298; doi:10.1001/jamapsychiatry.2023.1947)
Supplement: Supplement 1. — Trial protocol [file jamapsychiatry-e231947-s001.pdf]

CLINICAL STUDY PROTOCOL

Staged Treatment in Early Psychosis (STEP):
A SMART Trial of Interventions for Ultra High Risk of Psychosis Patients

**Protocol Number:**	HREC 2015.173

**Sponsor:**	Orygen, The National Centre of Excellence in Youth Mental Health<br>35 Poplar Road, Parkville, VIC 3052<br>AUSTRALIA
**Telephone:**	+61 3 9342 2800
**Facsimile:**	+61 3 9387 3003

**Coordinating / Chief Investigator:**	Prof Patrick McGorry<br>Orygen, The National Centre of Excellence in Youth Mental Health<br>35 Poplar Road, Parkville, VIC, 3052<br>AUSTRALIA

**Version:**	10
**Version Date:**	Tuesday, 6 October 2020

A note on terminology: the remission criteria described in the accompanying paper are referred to as response criteria in this protocol. Similarly, remitters and non-remitters are referred to as responders and non-responders.

Table of Contents

1. Table of Contents 2
2. List of Abbreviations 5
3. Study Synopsis 7
4. Schedule of Assessments and Endpoint Measures 11
5. Introduction 15
5.1. Background 15
5.2. Study Rationale 26
5.3. Study Aims 27
5.4. Hypotheses 27
5.5. Study Objectives 28
6. Study Design 29
6.1 Description 29
6.1. Study Setting 34
6.2. Study Endpoints 34
6.3. Study Duration 35
6.4. Study Risk Assessment 35
7. Participant Population 35
7.1. Description 35
7.2. Sample Size and Power 35
7.3. Participant Selection Record 36
7.4. General Considerations for Enrolment 36
7.5. Inclusion Criteria 36
7.6. Exclusion Criteria 38
7.7. Participants Restrictions 39
7.8. Discontinuation and Withdrawal 39
7.9. Replacement of Participants 41
7.10. Incorrectly enrolled Participants 41
7.11. Recruitment 41
8. Visits and Assessments 42
8.1. Study Measurements and Procedures 45
9. Study Intervention 49

9.1. Study Interventions: Doses, Administration and Treatment Regimen 49
9.2. Intervention Management 52
9.3. Concomitant Medication and Therapies 53
9.4. Method of Assigning Participants to Treatment Groups and Randomisation 54
9.5. Blinding 55
10. Safety Measures 57
10.1. Definitions: 57
10.2. Serious Adverse Event (SAE) 58
10.3. Unexpected Adverse Reactions 58
10.4. AE and SAE Assessment 59
10.5. Recording of Adverse Events and Serious Adverse Events 59
10.6. Expedited and Prompt Reporting of SAEs 60
10.7. Handling in Cases of Unresolved AEs and SAEs at Completion or Withdrawal 60
10.8. Pregnancy 61
10.9. Procedures in Case of Medical Emergency 61
10.10. Procedures in Case of Overdose 61
10.11. Managing participant distress in the EXPECT sub study 61
11. Study Oversight 61
11.1. Data Safety and Monitoring Committee 61
12. Ethical Considerations 62
12.1. Review by an Ethics Committee 62
12.2. Ethical Conduct of the Trial 62
12.3. Investigator Responsibilities 63
12.4. Financing and Insurance 63
12.5. Participant Information and Consent 63
12.6. Participant Data Protection 63
13. Study Management 64
13.1. Monitoring and Auditing 64
13.2. Audits and Inspections 64
13.3. Training of Staff 64
13.4. Changes to the Protocol 65
13.5. Protocol Compliance 65

13.6. Study Timetable and Termination 65
13.7. Data handling and record retention 65
14. Data Management 66
14.1. Documentation 66
14.2. Database management 66
14.3. Quality Control and assurance 67
15. Statistical Methods 67
15.1. Methodology 67
15.2. Analysis Populations 68
15.3. Interim Analysis 68
16. Publication and Use of Study Findings 68
References 70

1. List of Abbreviations

Abbreviation	Term in Full
**AE**	Adverse Event
**APS**	Attenuated Psychosis Syndrome
**ARMS**	At Risk Mental State
**CAARMS**	Comprehensive Assessment of At Risk Mental States
**CBT**	Cognitive Behaviour Therapy
**CBCM**	Cognitive Behavioural Case Management
**CHR**	Clinical High Risk
**CI**	Chief Investigator
**CRF**	Case Report Form
**CRP**	C Reactive Protein
**CSF**	Cerebrospinal Fluid
**CT**	Computed Tomography
**DSM**	Diagnostic and Statistical Manual
**DSMC**	Data Safety Monitoring Committee
**eCRF**	electronic Case Report Form
**ELISA**	Enzyme Linked Immunosorbent assay
**EXPECT in STEP**	Short title for optional parent sub-study
**FBE**	Full Blood Examination
**FPFV**	First Patient First Visit
**GCP**	Good Clinical Practice
**HDL**	High Density Lipoprotein
**HEADSS**	Home, Education, Activities, Drugs, Sexuality, Suicide and Depression
**HEAG**	Human Ethics Advisory Group
**HREC**	Human Research Ethics Committee
**HPA**	Hypothalamic-Pituitary-Adrenal
**ICH**	International Conference for Harmonisation
**IEC**	Independent Ethics Committee

**IP**	Investigational Product
**IRB**	Institutional Review Board
**LDL**	Low Density Lipoprotein
**LFT**	Liver Function Test
**LPLV**	Last Patient Last Visit
**MADRS**	Montgomery Asberg Depression Rating Scale
**MRI**	Magnetic Resonance Imaging
**NAPLS**	North American Prodrome Longitudinal Study
**NH&MRC**	National Health & Medical Research Council
**NIMH**	National Institute of Mental Health
**OYHCP**	Orygen Youth Health Clinical Practice
**Orygen**	Orygen, The National Centre of Excellence in Youth Mental Health
**PACE**	Personal Assessment and Crisis Evaluation
**PI**	Principal Investigator
**PICF**	Participant Information & Consent Form
**PT**	Preferred Term
**RCT**	Randomised Controlled Trial
**RDoC**	Research Domain Criteria
**RRC**	Research Review Committee
**SAE**	Serious Adverse Event
**SAP**	Statistical Analysis Plan
**SAR**	Serious Adverse Reaction
**SCID-5**	Structured Clinical Interview for DSM-V
**SMART**	Sequential Multiple Assignment Randomised Trial
**SOC**	System Organ Class
**SOP**	Standard Operating Procedure
**SPS**	Support and Problem Solving
**SUSAR**	Suspected Unexpected Serious Adverse Reaction
**TFT**	Thyroid Function Test
**U&E**	Urea and Electrolytes

**UHR**	Ultra High Risk

2. Study Synopsis

Item	Description
**Study Phase**	Phase III
**Date of Registration**	1/02/2016
**Indication**	Ultra High Risk (UHR) for Psychosis
**Sample Size**	N=500
**Total No. of Study Centres**	5
**Study Design**	Sequential Multiple Assignment Randomised Trial (SMART) of interventions for patients at Ultra High Risk of developing psychosis
**Study Intervention**	Step 1: Support and Problem Solving (SPS)<br>Step 2: Support and Problem Solving versus Cognitive Behavioural Case Management (CBCM)<br>Step 3: Cognitive Behavioural Case Management + Antidepressant Medication versus Cognitive Behavioural Case Management + Placebo
**Primary Objective**	To test the effect of a sequential treatment approach consisting of SPS/SPS and SPS/CBCM on functioning levels of UHR patients 6 months from baseline (end of Step 2).
**Secondary Objectives**	• To test the effect of a sequential treatment approach consisting of SPS, CBCM and antidepressant medication on functioning levels of UHR patients 12 months from baseline (end of Step 3).<br>• To test the effect of a sequential treatment approach consisting of SPS, CBCM and antidepressant medication on transition to psychotic disorder by 12 months and 24 months from baseline.<br>• To test the effect of a sequential treatment approach on UHR status (maintenance versus remission) 1.5 months, 6 months, 12 and 24 months into treatment.<br>• To test the effect of a sequential treatment approach in UHR patients on level of psychiatric symptomatology,

Item	Description
	including positive psychotic symptoms, negative psychotic symptoms and depressive symptoms 1.5 months, 6 months, 12 months and 24 months into treatment. To test relapse rates (to UHR Positive (UHR+) status) in the relapse prevention/responder arm of the trial (SPS v monitoring) during the first 12 months.
**Exploratory Objectives**	The study will explore moderators and mediators of response to treatment with a view to identifying vulnerability markers and mechanisms of therapeutic action. In the subset of responders to a specific intervention, we will examine whether it is likely that the treatment was effective due to putative psychological and neurobiological mechanisms. We will measure psychological variables at baseline, entry and exit from steps two and three and inflammatory and oxidative stress-related biomarkers, as well as antibodies to infectious agents, complement proteins and immunoglobins, at baseline, entry and exit from step three to test whether these variables moderate or mediate response to treatment.
**Study Endpoints**	**<u>Primary</u>**<br>Global Functioning at 6 months (end of Step 2).<br>**<u>Secondary</u>**<br>Symptoms and functioning 1.5 months into treatment (end of Step 1).<br>Symptoms 6 months into treatment (end of Step 2).<br>Symptoms and functioning 12 months into treatment (end of Step 3).<br>Symptoms and functioning 24 months post baseline (end of follow-up period).
**Study Population**	Males and females 12-25 years of age who meet UHR criteria.
**Inclusion Criteria**	1. Age 12 -25 years (inclusive) at entry.<br>2. Ability to speak adequate English (for assessment purposes).<br>3. Ability to provide informed consent.<br>4. Meeting one or more UHR for psychosis groups as defined in Table 1.
**Exclusion Criteria**	1. Past history of a treated or untreated psychotic episode of

Item	Description
	duration of one week or longer, whether treated with antipsychotic medications or not.<br>2. Attenuated psychotic symptoms only present during acute intoxication.<br>3. Organic brain disease known to cause psychotic symptoms, e.g. temporal lobe epilepsy.<br>4. Any metabolic, endocrine or other physical illness, e.g. thyroid disease, with known neuropsychiatric consequences.<br>5. Diagnosis of a serious developmental disorder, e.g. Severe Autism Spectrum Disorder.<br>6. A documented history of developmental delay or intellectual disability.<br>7. Previous or current SCID diagnosis of bipolar disorder I.
**Treatment Regimen**	Step 1: Support and Problem Solving.<br>Step 2: Support and Problem Solving versus Cognitive Behavioural Case Management<br>Step 3: Cognitive Behavioural Case Management + Antidepressant Medication versus Cognitive Behavioural Case Management + Placebo
**Duration of Treatment**	Step 1: 1.5 months<br>Step 2: 4.5 months<br>Step 3: 6 months<br>Follow up: 12 months<br>Total study duration per participant = 24 months
**Concomitant Therapy**	**Authorised medication**<br>Sedative-hypnotics (benzodiazepines and benzodiazepine-like medications).<br>**Unauthorised medication**<br>Antidepressants in Steps 1 and 2.<br>Antipsychotics and mood stabilisers.
**Control Group**	Step 1: All participants will receive SPS.<br>Step 2: SPS will be compared with CBCM.<br>Step 3: CBCM+SSRI will be compared with CBCM +placebo.<br>Participants in these steps of the trial will be compared with participants who respond to Steps 1 and 2 and are randomised to SPS or Monitoring.

Item	Description
**Study Duration**	FPFV06/2016<br>LPLV: 01/2021
**Data Analysis**	Data analysis is scheduled for Q4 2020. No interim analysis is planned.
**Statistical Methods**	The sample size has been determined using previously collected UHR data and is based on the primary outcome of six-month functioning between the two treatment groups. The primary analysis will compare the two Step 2 treatments using analysis of covariance. Other secondary outcome measures will be examined using ANCOVA, chi-square tests, survival analysis and multi-level modelling.
**Funding Provided by**	NIH Cooperative Agreement

99
3. Schedule of Assessments and Endpoint Measures

VISIT NUMBER	1	2	3	4	5	6	7	8	9	10	11
			STEP 1 (1.5 months)		STEP 2 (4.5 months)		STEP 3 (6 months)[ϖ]			FOLLOW UP[ϕ, ϖ]	
	Day -21 to Day 1		Month 1-1.5		Month 3[ϖ]	Month 6[ϖ]		Month 9	Month 12	Month 18	Month 24
	Screening (Day -21 to Day 1)	Baseline (Day 1)	Week 4[##^]	Week 6[##^]	Week 12[##^]	Week 24[##^]	Week 25[##^]	Week 36[##^]	Week 52[###]	Week 78[###]	Week 104[###]
**Intervention administered**			SPS[1]		SPS V CBCM[2]		CBCM + SSRI V CBCM + Placebo[2]				
**Assessments**											
Informed Consent	X										
Inclusion/Exclusion Criteria[#]		X									
Demographics	X										
Medical History[#]	X										
Pregnancy (blood)							X				
Randomisation				X			X				
**Safety**											
Clinical Bloods (haematology, biochemistry)							X		X		
Physical Examination	X						X		X		
Adverse Events [3]	X	X	X	X	X	X	X	X	X	X	X
Concomitant Med. Review	X	X	X	X	X	X	X	X	X	X	X
**Psychopathology**											
PQ-16	X										
SCID_5		X				X			X		X
SCID-II PD	X	X							X		X
CAARMS[6]	X	X	X	X	X	X		X	X	X	X

VISIT NUMBER	1	2	3	4	5	6	7	8	9	10	11
			STEP 1 (1.5 months)		STEP 2 (4.5 months)		STEP 3 (6 months) ϖ			FOLLOW UP ϕ, ϖ	
	Day -21 to Day 1		Month 1-1.5		Month 3 ϖ	Month 6 ϖ		Month 9	Month 12	Month 18	Month 24
	Screening (Day -21 to Day 1)	Baseline (Day 1)	Week 4 ##^	Week 6 ##^	Week 12 ##^	Week 24 ##^	Week 25 ##^	Week 36 ##^	Week 52 ###	Week 78 ###	Week 104 ###
**Intervention administered**			SPS[1]		SPS V CBCM[2]		CBCM + SSRI V CBCM + Placebo[2]				
**Assessments**											
BPRS		X		X		X		X	X	X	X
SANS		X		X		X		X	X	X	X
MADRS		X		X		X		X	X	X	X
FHI		X									
ASSIST		X			X	X		X	X	X	X
DACOBS[4]		X		X		X			X		
SQUEASE		X									
CTQ[4]		X									
Effects of psychological therapy scale (self-report)									X		X
Working Alliance Inventory (client self-report)						X			X		
Working Alliance Inventory (clinician self-report)						X			X		
CAARMS positive symptoms[8]			These assessments will be conducted post-transition[8].								
SCID V[8] mood module (the relevant sections to derive a psychotic mood diagnosis) and the psychosis module											
GFS&R[8]											

VISIT NUMBER	1	2	3	4	5	6	7	8	9	10	11
			STEP 1 (1.5 months)		STEP 2 (4.5 months)		STEP 3 (6 months)[ϖ]			FOLLOW UP[ϕ, ϖ]	
	Day -21 to Day 1		Month 1-1.5		Month 3[ϖ]	Month 6[ϖ]		Month 9	Month 12	Month 18	Month 24
	Screening (Day -21 to Day 1)	Baseline (Day 1)	Week 4[##^]	Week 6[##^]	Week 12[##^]	Week 24[##^]	Week 25[##^]	Week 36[##^]	Week 52[###]	Week 78[###]	Week 104[###]
**Intervention administered**			**SPS[1]**		**SPS V CBCM[2]**		**CBCM + SSRI V CBCM + Placebo[2]**				
**Assessments**											
**Functioning & Quality of Life**											
SOFAS	X	X	X	X	X	X		X	X	X	X
Global Functioning: Social & Roles Scales		X				X			X	X	X
AQoL-8D		X			X	X		X	X	X	X
**Biological[5]**											
Research bloods		X					X		X		
Genetic sample (blood or saliva)		X									
Hair Cortisol		X			X		X		X		
Cerebrospinal Fluid (CSF)[5 7] *(including clinical safety bloods and brain imaging prior to lumbar puncture)*[9]					X			X			
**Other**											
Medication Compliance Checklist[10]								X			
Neurocognition[5]		X				X			X		
EXPECT in STEP Sub study[11]							X				
Participant Perspectives Questionnaire (SPS and CBCM)[12]						**X**					

Notes.

[1.] SPS sessions delivered as clinically indicated over 6 weeks (1.5 months)
[2.] Weekly sessions or as clinically indicated.
[3.] Participants will also have regular (ideally weekly-fortnightly) contact with their treating team (case managers and/or psychiatrist), during which risk will be monitored.
[4.] Self-reports.
[5.] Optional components only to be conducted in participants consenting accordingly.
[6.] The full CAARMS will be administered across all study visits as indicated, with the exception of the screening visit and post-transition assessment; for which only the positive symptoms component of CAARMS will be administered.[7.] The CSF collection may be conducted at two time points. The first sample will be collected during the middle of Step 2, and the second sample (optional) will typically occur at Week 52 or when the client ceases the on study participation i.e. if they are discontinued.
[8.] Transition is operationally defined via the CAARMS, as in previous studies (142), as positive psychotic symptoms occurring every day for one week or longer.
[9.] Clinical safety blood testing will be conducted prior to the lumbar puncture being performed to ensure the participant's blood clots normally. The test will be performed in advance of the lumbar puncture and only participants with normal clotting levels will undergo the lumbar puncture. Brain imaging via an MRI scan, will also be conducted prior to the lumbar puncture to check for the presence of a brain tumour or other lesion. The procedure will be performed in advance of the lumbar puncture and only participants whose imaging results confirm the absence of brain tumour or lesion will undergo the lumbar puncture.
[10], Once in Step 3, participants will be contacted by study staff every fortnight to complete a medication compliance questionnaire so that we can assess their compliance with the study medication.
[11] Optional Parent sub study titled 'EXPECT in STEP'. The parents of consented participants aged between 15 and 18yrs will be approached following their child's verbal consent to determine if they would like to consider consenting to a parent substudy. The consent process and subsequent interview can occur at any timepoint in the schedule post Day 1 of STEP.
[12] This questionnaire will be administered with all participants after they have ceased the treatment phase of the study. For responders and those who have withdrawn/discontinued, this may be earlier than the month 12 time point.
[#.] Assessments performed by a medical doctor and all participants will be medically reviewed by the end of Step 1.
[##] Study visits 3-8 have a window of ± 7 days
[###] Study visits 9-11 have a window of ± 30 days
[^] Data for these study visits can be captured retrospectively at the proceeding visit
[ϕ] These assessments/visits done by all participants (including Step 1 and Step 2 responders)
[ϖ] All follow-up visits for responders are to contain the same assessments as the corresponding non-responders progressing through Steps 2 and 3.

4. Introduction

4.1. Background

Psychotic disorders are amongst the most disabling and costly disorders for health care systems (1), and early intervention is now seen as the most promising strategy to improve outcomes (2-4). In standard care, long delays in treatment occur routinely and are associated with many damaging psychosocial consequences as well as risk of self-harm, violence, suicide and homicide (5-9). Wyatt (10) proposed such delays could be responsible for much of the disability and that reducing the duration of untreated psychosis prior might improve outcomes. As early intervention services were constructed from the early 1990s to reduce treatment delay and provide phase specific care (11), an even greater opportunity for preventive intervention was highlighted (5, 11). For most patients, an often prolonged ("prodromal") period of subthreshold symptoms and impaired functioning precedes the first psychotic episode (12-19). In fact, much of the disability develops long before the onset of sustained psychosis and is typically difficult to reverse (20, 21). The fact that a clear-cut need for care exists prior to sustained positive psychotic symptoms indicated that intervening at this stage might lead to more profound preventive benefits, through delaying, or even preventing the onset of fully-fledged disorder.

In 1994, our group at Orygen developed operational criteria for prospectively identifying help-seeking young people at increased (incipient) risk of a first episode of psychosis (FEP) within 1-2 years (11, 22). This clinical population was initially described as having an "at risk mental state" (ARMS)(11), more specifically patients were deemed at "ultra high risk" (UHR) for psychosis, and were subsequently referred to in the USA as a prodromal or "clinical high risk" (CHR) group (23). The development of these criteria stimulated a wave of new research internationally over the past two decades (24, 25). A recent meta-analysis comprising approximately 2,500 individuals who met UHR criteria reported mean transition rates of 22% at 1 year, 29% at 2 years, and 36% at 3 years (26). The first long-term follow up study of UHR patients, recently completed by our group, indicated that the risk period is concentrated within the first 3 years, yet extends for up to 10 years (27).

The US-based NAPLS group has conducted multicenter studies examining predictive and potential aetiological factors in a large US cohort (28-30). On the basis of this body of international research, a diagnosis of Attenuated Psychosis Syndrome (APS) was proposed for inclusion in the 5th Edition of the Diagnostic and Statistical Manual (DSM). Due to concerns about the reliability of diagnosing this putative syndrome in routine clinical settings, the potential risks of overtreatment and a move away from the inclusion of new

categories, the DSM committee decided that more research was needed, and included APS in Section III of the new manual, "Conditions Requiring Further Research".

UHR Intervention Research

Intervention research in the UHR group has had two principal foci:

a) The treatment of existing symptomatology and disability and

b) The prevention of transition to psychotic disorder (indicated prevention).

Since our first intervention trial in the late 1990s (31), ten further RCTs have been conducted, testing a range of psychological, pharmacological, nutritional and multicomponent psychosocial interventions. A recent meta-analysis (32) found that the risk reduction of experimental interventions compared to control interventions was 54% over 12 months with a Number Needed to Treat (NNT) of 9. Cognitive-behaviour therapy (CBT) has been found to be of particular benefit and safety, with another meta-analysis indicating that it reduces the relative risk of developing psychosis by 50% at 6, 12 and 18-24 months (33). Integrated psychological interventions have also been shown to be of benefit (34, 35). Antipsychotic medication has also shown some efficacy, although a less favorable risk-benefit ratio means that it is not appropriate for first line therapy (36).

Omega-3 fatty acids have shown a strong and sustained benefit in one double blind placebo controlled trial (4.9% versus 27.5% at 12 months, p<.007) (37). In this study the differences remained significant at 6-7 year follow up (*Amminger, unpublished data*). Attempted replications are currently underway via a large (n=304) international RCT led by the current PI, and an additional US study conducted by the NAPLS group. The initial data from our own replication study indicates that Omega-3 fatty acids confer no advantage over CBT in first line treatment but may be useful still in a subgroup who have failed to respond to CBT.

Cornblatt et al (38) and Fusar-Poli et al (39, 40) reported naturalistic data suggesting that antidepressant medication may be effective in reducing the risk of transition. Antidepressants could reduce risk by improving mood and thereby indirectly reducing faulty appraisal of anomalous experiences linked to future psychosis. They may also modulate response to environmental stress and adversity, which has been found to increase risk for psychosis (39, 41). CBT may reduce risk through similar mechanisms. This effect may occur either directly via acting on the neurochemical pathways that mediate stress response (42) or indirectly by preventing depression and anxiety secondary to these stressors (43). Indeed, Garner and colleagues (44) reported that a 10% increase in pituitary gland volume in at risk individuals was associated with a 20% increase on transition to psychosis, suggesting that stress hormones may play a role in the onset of psychosis. Consistent with this, Cornblatt and colleagues (45) proposed that antidepressants may reduce some of the underlying core vulnerability factors (mood disturbance, social isolation, school failure) and thereby relieve

the stress that might otherwise exacerbate attenuated psychotic symptoms. It also appears that antidepressants may possess some neuroprotective properties (46-48), which may halt a neurodegenerative process associated with the evolution of psychotic disorder (see below).

Longer term follow up of the relatively brief interventions trialled to date reveals significant differences in transition tend to be lost over time (36, 49, 50). This suggests that the intervention for most patients may have merely delayed onset through reducing risk during the period of treatment. It further suggests different treatments, sequences and durations of therapy may be needed for sustained benefits, as seen with early psychosis services in general (51-55).

The first generation of UHR trials have mostly been modest single site studies testing one or two candidate therapies in single step, head to head designs. The next step is for further trials to determine the most effective type, sequence and duration of interventions, and how they might best be personalised or tailored to individual patients, based on clinical presentation, response profile and perhaps ultimately through the use of biomarkers or psychosocial variables.

Broadening the outcomes of interest: Functioning and non-psychotic outcomes

The UHR criteria have shown good predictive validity for progression of the psychosis phenotype, consistent with their original purpose. However, this special valence for psychosis outcomes has been shown to coexist with an increased risk of persistent subthreshold psychotic symptoms (41%), other non-psychotic syndromal outcomes (up to 40%) and a cross-diagnostic risk of poor functional outcome in a substantial minority (56-59). Furthermore, while the symptomatic target outcome of “transition” to sustained psychosis is associated with a greater risk of poor outcome, this is not necessarily always the case since rapid and quality treatment of full threshold psychosis post-transition can lead to full remission and sustained functional recovery (3). Conversely, persistent subthreshold positive symptoms and/or mood and anxiety symptoms may seriously disable, and these syndromes are often not treated due to poor access to or engagement in subsequent care. Adopting a broader perspective for UHR treatment is congruent with the general move to prioritise social and occupational recovery as a primary target for intervention in all mental disorders (60).

Declining transition rates and need for larger-scale, sequential enriched studies

Another recent observation has been the declining transition rate in UHR cohorts worldwide. Early studies reported 12-month transition rates in the 30-40% range whereas more recent studies have reported rates of 10-20% (26, 61). The level of incipient risk is however still well in excess of that seen in risk scenarios for serious physical illnesses, such as ischemic heart

disease and diabetes, where risk reduction strategies are well accepted (62, 63). The fact that UHR patients are not only already symptomatic but impaired underlines the need to clarify an evidence-based intervention sequence which may be translated directly into clinical practice. However, these factors do reduce power and underline the need for larger sample sizes and sequential strategies to enrich for risk.

Innovation

There are multiple innovative aspects to the current study:

1. We have developed a clinical staging model, similar to the US National Institute of Mental Health Research (NIMH) Research Domain Criteria (RDoC) strategy, blending categorical and dimensional characteristics of psychopathology. It has the additional advantage of enabling the benefits of interventions to be balanced against risks, and the role of biomarkers in prediction of response and outcome to be elucidated.
2. The study of biological and psychological markers in relation to response to individual treatments within trials as proposed here is a novel feature in UHR research.
3. Sequential Multiple Assignment Randomized Trial (SMART) methodology has not yet been deployed in UHR trials and is the next logical step given the meta-analytic evidence of efficacy of a range of therapies and the emergence of candidate experimental, mechanism-based therapies. It is also congruent with the principles of clinical staging and the practice of stepwise clinical care.
4. The **headspace** innovation has created a new stigma-free clinical infrastructure in Australia that makes the rapid recruitment of participants to a larger "n" SMART trial design feasible for the first time.

The Clinical Staging Model

The principles underpinning the preventive approach to emerging psychotic illnesses in young people have a potentially wider application to other disorders with which they overlap, and in the earliest stages, seem relatively indistinguishable in terms of clinical phenotype (4, 64). NIMH has recognized the same need for a major conceptual rethink through its development of the RDoC (65-67). The clinical staging model, developed by our group is a heuristic approach similar in intent to the RDoC strategy to transcend traditional diagnostic boundaries aimed at understanding the neurobiological and psychosocial processes underpinning the onset and course of mental disorders. Equally, clinical staging, through integrating stage and timing with evolution of clinical phenotype, allows interventions to be tested from a preventive standpoint in reducing the risk of progression and persistence of illness, with less risk and greater potential benefit (68-71). Staging is a pragmatic blend of categorical and dimensional approaches which aims for clinical and research utility, providing a heuristic framework to study the significance of biomarkers by stage and clinical phenotype, psychosocial risk factors and treatment response. Importantly,

this wider staging model allows us to include other syndromal and functional targets as outcomes in clinical trials.

Applying the clinical staging model to UHR intervention means that a staged approach to treatment is offered, with the safest, most benign and least specialised interventions used initially, and "stronger", more intensive interventions, with potentially more adverse effects, reserved for those who do not respond to the earlier stages of intervention. This adaptive strategy also addresses the lower transition rate through a stepwise enrichment of the sample such that non-responders to initial simpler interventions will be enriched for both higher transition rates to psychosis and for greater functional impairment. The staging model also hypothesises that timing of intervention is critical in that therapeutic agents/strategies with neuroprotective and psychoprotective qualities will be most beneficial in this subthreshold stage of disorder in reducing the impact of the disease process. Finally, this staged approach to UHR intervention importantly addresses ethical concerns regarding intervention in this group, namely the "false positive" issue (particularly given the reducing transition rate globally), potential stigma, and a perceived relative lack of predictive power (72). Since there is a need for care for the presenting clinical problems, then providing safer, nonspecific and benign interventions as a first step will allow those with milder and responsive or self-limiting problems to remit, while those who fail to benefit can progress to more intensive or relatively more specific interventions. As described, this ensures an "enriched" sample, with fewer false positives for both persistent and more severe illness (of any phenotype) and also for transition to psychosis, in which to test more specific intervention strategies in the context of a rigorous clinical trial.

Neuroprotection, Inflammation and Oxidative Stress

Current biological treatment options for established psychoses have predominantly centered on the dopamine theory. However, it is unclear how central this theory is likely to be for UHR patients (73, 74). In addition, the side effects of dopamine antagonists (i.e., antipsychotic medications) significantly limit their preventive use. There is now a clear body of evidence to support a role for both inflammation and oxidative stress in the pathophysiology of psychotic disorders (75, 76). There is consistent evidence of increased blood concentrations of inflammatory cytokines (77). Inflammatory abnormalities are present in subjects with first-episode, drug-naïve psychosis (FEP) compared with controls, suggesting an association independent of antipsychotic medication. Furthermore, the concentrations of some inflammatory molecules vary with the clinical status of patients, i.e., there appear to be separate groups of state and trait markers. State-related markers include interleukin (IL) 1-beta, IL-6, and transforming growth factor-beta. IL-12, interferon-gamma, and tumor necrosis factor-alpha (TNF-alpha) appear to be trait markers (78). Oxidative stress, a state where the levels of available antioxidants is reduced, or the levels of oxidative species is increased, is well documented in schizophrenia and related psychoses (75), with abnormal

oxidative stress parameters reported in peripheral blood cells (i.e., erythrocytes, neutrophils, platelets), cerebrospinal fluid and postmortem brain. Inflammation and oxidative stress also reciprocally induce each other in a positive feedback manner (79). The incorporation of these factors into traditional monoamine neurotransmitter-system models facilitates a more comprehensive model of disease, potentially underpinning course of illness in psychotic disorders (80). This in turn has facilitated the identification of new therapeutic targets and neuroprotective treatments that have the potential to interrupt the identified neurotoxic cascades (81). Although the current study will not test a pharmacological agent that targets these potential pathogenetic mechanisms, we will be measuring relevant biological markers to assess their predictive value and whether they moderate outcome. As mentioned above, it is possible that antidepressant medications have neuroprotective properties.

"Psychoprotection"

The cognitive model of psychosis posits that dysfunctional interpretations of anomalous experiences contribute, against a background of pre-existing biopsychosocial vulnerability, to the emergence and maintenance of psychotic symptoms (82, 83). These consist of cognitive biases, such as jumping to conclusions, selective attention or mental "filtering", confirmatory bias, attributional biases, "all-or-nothing" thinking, overgeneralization, and personalization (84). They are thought to emerge from underlying "core" beliefs or schemas about the self and world that have become entrenched during an individual's developmental years. During the prodromal stage of psychosis, it is thought that such biases contribute to the crystallisation of psychotic-like experiences into frank psychotic symptoms (85, 86). CBT for UHR individuals aims to identify, manage and correct these cognitive biases and vulnerabilities through a variety of talking therapy and behavioural techniques and thereby diminish the risk of developing full-threshold psychotic symptoms (86, 87). In this way, CBT may function as a form of "psychoprotection" against cognitive vulnerabilities. In several trials to date, it has proven effective (NNT=13) though a link to the underlying mechanism of reducing cognitive biases and vulnerabilities, a focus of the present design, has not yet been clearly demonstrated. The use of CBT as a psychological therapy has a number of advantages: (a) CBT is usually more acceptable, tolerable and less stigmatising to patients than medications, particularly antidepressant and antipsychotic medications (88, 89), (b) CBT has already been shown to be effective in a number of previous trials in UHR (31-33, 90-92) and does not expose "false positives" for psychosis transition to pharmacological side effects, and (c) CBT (if delivered flexibly) provides evidence based treatment for such "false positives", who although do not go on to develop psychosis, typically already suffer from or are vulnerable to other disorders, such as mood and anxiety disorders. Although CBT has been found to be more effective than monitoring it is not yet clear whether it is more effective than other, less intensive psychotherapies. In a trial comparing risperidone, CBT

and supportive therapy, supportive therapy resulted in the same degree of symptomatic and functional improvement in UHR patients as CBT (93, 94). The current study will test cognitive-behavioural case management (CBCM), an intervention that delivers CBT in a case management format. It will therefore allow for further investigation of whether CBT provides additional advantages over supportive therapy for this patient group.

Other psychosocial interventions may also be of benefit in this group, including yoga and mindfulness. A recent study by Chen and colleagues (95) found that a group-based yoga intervention improved cognitive performance and reduced psychotic and depressive symptoms in an early psychosis group, as well as being associated with structural brain changes. There is evidence that mindfulness interventions show moderate benefits for symptoms in psychotic samples, particularly for negative symptoms, including preliminary evidence for its potential utility in early psychosis patients (96).

Neurocognitive and neurobiological predictors of psychosis

Neurocognitive impairment is present at the onset of schizophrenia (97). There is considerable literature showing that reduced neurocognitive performance predates the onset of frank psychotic symptoms and is evident in childhood (98) and adolescence (99) in people who go on to develop schizophrenia. UHR patients also show reduced neurocognitive performance compared to healthy controls across a range of domains (100). Within this group, those who transition to psychosis show even greater decrements. The precise pattern of neurocognitive deficits in those who transition is yet to be determined. However, the most consistent findings relate to reduced memory and executive function performance (29, 101-109).

Neurocognition will be assessed in the current study in order to probe the cognitive deficits that mark the pre-onset phase of psychotic disorder and whether neurocognitive profiles predict outcome and moderate/mediate response to treatment.

Biological processes underlying psychosis

Identifying the biological processes that underlie psychotic disorders remains an important goal in human genetics (117). There are over 100 loci in the human genome containing single nucleotide polymorphisms (SNP) haplotypes which associate with risk of schizophrenia, although the function and mechanisms of the alleles at these loci are yet to be determined (118). Current research indicates that the strongest genetic relationship for schizophrenia is association with genetic markers across the major histocompatibility complex locus (MHC) (119-123). The MHC plays a key role in immunity, and contains 18 highly polymorphic human leukocyte antigen (HLA) genes which encode a range of antigen presenting molecules (124,125). Although the association between schizophrenia and the MHC locus has not yet

been explained, evidence indicates that the association arises in part from alleles of the complement component 4 (C4) genes (126).

More recently, Sekar *et al* (127) found that across a set of common, inherited alleles that affect CNS expression of complement component 4 (C4) genes, the risk of schizophrenia increases in proportion to C4A expression. This study demonstrated that post-mortem brain tissue from individuals affected with schizophrenia exhibits on average a 40% elevation in C4 RNA expression, relative to tissue from individuals not affected by schizophrenia. Importantly, this relationship is many times stronger than can be explained by the genetic effect found at C4 (127). A natural interpretation is that this relationship reflects additional influences on C4 expression in the CNS - for example, environmental factors or other unknown genetic factors that also elevate risk. Another possibility is that C4 expression levels also reflect the progression of the illness. Therefore, C4 expression levels contain information above and beyond that what can be obtained from a genetic test.

The mechanism by which C4 expression may influence onset of psychotic disorder is via synaptic pruning. C4 promotes synaptic pruning, i.e. synapse elimination via engulfment by microglia, the phagocytic immune cells of the CNS, which express receptors for complement proteins. Such waves of synapse elimination are known to occur in the human cerebral cortex, intensifying in many critical cortical regions during the second and third decades of life. Excessive loss of gray matter and abnormally low numbers of synapses on certain kinds of cortical neurons in these brain regions (i.e. excessive synaptic pruning) are well-replicated pathological findings in schizophrenia. Complement proteins and their implication of synaptic pruning offer a potential mechanism that may explain these pathological observations. The current study provides a valuable opportunity to extend recent major findings by *prospectively* assessing the role of C4 expression in the onset of psychotic disorder (127).

The hypothalamic-pituitary-adrenal (HPA) axis response to stress and baseline activity of the HPA axis are altered in psychotic disorders, with higher baseline cortisol levels, blunted awakening response and blunted stress response commonly observed throughout the literature (e.g. Berger et al. 2016, Neurosci Biobehav Rev 68:157-166, Ciufolini et al. 2014, Neurosci Biobehav Rev 47:359-368). These findings are supported by studies showing that stressful life experiences can trigger or worsen psychotic symptoms (e.g. Reininghaus et al. 2016, Schizophr Bull 42:712-722). A recent meta-analysis demonstrated altered cortisol awakening response in psychotic disorders including in first-episode psychosis, with two studies providing evidence that HPA-axis activity is related to transition outcomes and predictive of treatment response (Berger et al. 2016, Neurosci Biobehav Rev 68:157-166). However, these studies relied on small samples and it remains unknown if HPA-axis activity

relates to outcomes other than transition to psychosis.

Gene-environment interaction

The high heritability of schizophrenia provides a critically important route of inquiry into the pathophysiology of the disorder. In main effects models, where gene variants are considered individually, common variants are only very weakly associated with schizophrenia. Even combined, all the common variants that have been significantly associated with schizophrenia are thought to account for less than 2% of the total estimated genetic risk (117). The application of novel genetic technology to schizophrenia has also established that rare fairly high penetrance copy number variants (CNVs) may account for a small proportion of the genetic liability of schizophrenia (119). However, unpublished analyses of the International Schizophrenia Consortium (ISC) single nucleotide polymorphism (SNP) data along with other datasets suggest that at least half of the genetic variance results from alleles of small effect (Purcell, presented on behalf of the ISC, ISPG, 2008, Osaka).

A separate and concurrent line of research, mainly in the area of epidemiology, has established that there are high rates of schizophrenia in large cities, immigrant populations, traumatised individuals and cannabis users, at least some of which is thought to be the result of underlying environmental exposures (128, 129). However, the biological mechanisms underlying these established environmental risk factors are largely unknown.

Encouraging findings in other areas of medicine have motivated researchers to turn their attention to better understanding the complex ways in which nature interacts with nurture to produce schizophrenia. This genotype x environmental interaction (GxE) approach differs from the linear gene-phenotype approach by positing a causal role not only for either genes or environment in isolation, but also for their synergistic co-participation in the cause of schizophrenia where the effect of one is conditional on the other (130, 131). For example, genes may moderate the effects of certain drugs of abuse, or the environment may moderate the level of expression of a gene associated with schizophrenia. The current study will collect biological samples of blood and CSF for genetic analysis in order to investigate whether GxE interactions predict outcome and moderate/mediate response to treatment. This will build on the work of studies currently underway, such as the European Union-Gene Environment Interaction in the Onset of Schizophrenia Study (http://www.eu-gei.eu), which Orygen is participating in.

Owing to the relatively invasive nature of CSF collection via lumbar puncture, it would be preferable if C4 levels could be monitored in plasma. However, seeing that C4 and other complement proteins are not thought to cross the blood-brain barrier, measuring the plasma levels of C4 might not accurately reflect the levels of C4 in the CSF. In order to assess this,

our collaborators at The Broad Institute sought to understand the extent to which plasma C4 levels might have information about CSF C4 levels, since this route would ask less of research participants. They measured C4 levels in CSF and plasma samples from the same 50 donors, asking whether CSF and plasma levels of C4 are correlated across individuals. They found that although CSF and plasma levels of C4 each exhibit substantial inter-individual variation, the inter-individual variation in these two compartments is almost completely uncorrelated. For example, the five individuals with the greatest elevation in CSF C4 levels (five times the median CSF C4 level), all had below-average levels of C4 in their plasma samples. These results suggest that plasma analysis will have little information about C4 levels in CSF. Based on these results, we have concluded that analysis of CSF is crucial for monitoring CNS-relevant levels of C4.

We do not specify the exact genes (or more appropriately, genetic markers) to be analysed in this study. This is for two reasons: i) much of genetic research at present is 'genome-wide', i.e. is not hypothesis driven but based on unbiased discovery approaches. Our study will include genome-wide hypothesis generating approaches (such as proteomic or gene expression analysis) which would generate novel genes to look at and therefore it is not possible to say in advance which genetic markers we will examine, ii) the field is changing very quickly; new polymorphisms which are associated with schizophrenia are emerging at an increasing pace. We will use specific genetic polymorphisms including known schizophrenia risk alleles (for example those near TCF4, CACNA1C, TCF7L2, HLA, ZNF804A, miR137) and CNVs (deletion sin neurexin 1, 15q13.3 etc.) and also include novel genetic polymorphisms that might emerge during the lifetime of the project.

It is important to translate such genetic and biological findings into tools and therapies that can contribute to the diagnosis and treatment of patients. While measurements of RNA in *post mortem* brain tissue are useful for determining biological mechanisms, they are not clinically useful because they can only be obtained from brain tissue post mortem. It would be far more valuable clinically to have biomarkers for risk or progression that could inform the care of living individuals, that could be obtained without sampling brain tissue, and that could be used to monitor dynamic biological processes that unfold over time – such as adolescent synaptic pruning and other sequelae of neuron-microglia interactions. As indicated above, C4 RNA expression holds promise for being such a biomarker. Such biomarkers would also facilitate the evaluation of future, innovative therapeutics that seek to modulate these processes.

"SMART" Study Design

Creating an evidence base fit for implementation and for subsequent clinical translation requires a trial methodology which experimentally evaluates interventions via multistage designs, and which builds upon methods intended for the analysis of naturalistically observed strategies. A clinical trial design innovation, the Sequential Multiple Assignment Randomized Trial ("SMART"), used in several recent large scale studies in psychiatry to develop an evidence base to support adaptive clinical care, is a good fit with the clinical staging model, and is ideal for the present study. SMART designs involve multiple intervention stages that correspond to the critical decisions involved in adaptive interventions. Adaptive interventions are interventions in which the type or dosage of the intervention is individualised on the basis of patient characteristics or clinical presentation and then are repeatedly adjusted over time in response to patient progress (132). Interventions can also be tailored at critical decision points according to response or other patient characteristics such as specific biomarker changes or comorbidity, and also patient preference (133). Using this approach, the study should have important implications for clinical practice, as the clinical situation often requires decisions about how to adapt the intervention based on patient progress or preference.

Internationally Unique Stigma-free Enhanced Primary Care Platforms

The Australian **"headspace"** system is a nationwide platform of care for young people, which has already enabled access to clinical and social care to over 100,000 young Australians aged 12 – 25 years (inclusive) with emerging mental ill health. The system is essentially a "one stop shop", universal access model for young people and families experiencing early stage mental and substance use disorders. Our research centre conceived, designed and implemented this reform from 2006-2009, and it has now expanded across the nation to 70 centres and will expand to 100 centres by 2016. Some early literature and data on the **headspace** model have been published (133-136). Orygen directly operates 4 of these platforms with an annual throughput of over 5000 young people. Unpublished research data gathered from these sites (n=800) indicate that 38% of the patients meet the UHR criteria. We recently reported on a large baseline cohort of patients (n=21,274) assessed through the full headspace system of care nationally (137).

Understanding engagement and ethical concerns: In studies among persons at-risk of psychosis, several ethical dilemmas have been identified (160). For those young people at risk of psychosis, both being identified as being at risk and receiving interventions can be explored further within the four ethical principles of a) beneficence, b) non-maleficence, c) respect for autonomy and d) justice. Overall, for risk identification and preventive interventions, the balance of benefits and risks should be balanced against principles of autonomy, with a broader consideration of principles of distributive justice (161). Despite the explosion of research information regarding risk prediction for psychosis, very little is

known about participants' perspectives regarding being at-risk. While several studies in psychosis prediction include participants who are at risk, there is little information on how much they understand about the nature of risk, and the uncertainties regarding risk for serious mental disorders. Their appreciation of the balance of risks and benefits is also poorly studied.

While such assessment would be important with respect to young people at risk, it may also be important to consider their parents, as the legal guardians. A previous qualitative study indicated that when young people were considered to be at risk of future mental disorders, parents expressed a preference for these young people to not know that they are at a greater risk (163). Thus, a first step would be to understand parents' understanding of young people's risk as well as the various ethical imperatives associated with risk identification. This could be followed up with a future study that explores similar issues among young people, incorporating parents' views.

4.2. Study Rationale

Given the background outlined above, we propose a carefully formulated program of translational research with two linked elements. The first element is designed to create new knowledge and skills for intervening in the early clinical stages of psychotic disorders. The heterogeneity of the UHR stage of illness means that individualised "adaptive" treatment policies are required and a SMART design indicated. Some patients will respond while others will not to the same intervention type, intensity or duration. Other reasons in support of adaptive and sequential approaches include the need for sample enrichment, changing responsiveness linked to stage of illness, presence of comorbidities and the high risk of relapse even when initial remission has been achieved (24).

The second element involves the processing of this emerging new evidence and experience and adapting and translating this to key parts of the US health care system. This will be the subject of a separate Human Research and Ethics Committee application in the US. Through a series of focus groups with key stakeholders, potential challenges and solutions to US implementation across public and private healthcare will be identified. Open-label use of the study interventions in a UHR outpatient clinic in the US will assess the feasibility and acceptability of the staged intervention model and study treatments.

This model of research is ambitious and, due to the target clinical phenotype and the potential cost-effective impact, could be truly ground-breaking. Collectively this group of investigators has the most extensive cumulative experience and track record in this research field, the most congruent research design, and we have assembled for the first time the necessary clinical infrastructure and methodology to enable this research program to be

successfully completed and its findings to be translated into US healthcare. The investigators believe that this Australian-US collaboration is ideally placed to make this much-needed and now achievable contribution to new knowledge, to evidence informed care in early intervention and to enhanced patient and family outcomes.

In summary, the proposed study's sequential multi-stage design is intended to produce evidence to guide a stepwise clinical approach to treatment of UHR patients and reduction of risk for psychosis and other deleterious clinical and/or functional outcomes. The study will also collect data on key biomarkers and other mechanisms potentially underpinning risk, which may ultimately be used to adapt and personalise treatment. Major recent service reforms and expansions in Australia have enabled our research centre to access much larger clinical samples through which we can make a crucial contribution to assembling an evidence base that, with the assistance of the US investigators, will be translatable to US health care, and other settings worldwide with the goal of reducing the enormous human, social and economic costs of schizophrenia and other psychotic disorders.

An examination of parents' perspectives and beliefs about their children being identified as at risk for psychosis, or other serious mental disorders would be enlightening within the context of this study. This information could be key to improving the process of obtaining consent in prediction research and disseminating clinical and research information regarding participants being at-risk. This could also lay the foundation for future research which could help clinicians and researchers discuss information regarding new interventions in at-risk participants and in turn help new interventions such as decision aids.

4.3. Study Aims

The main study aim is to test outcomes of UHR patients, primarily functional outcome, in response to a sequential intervention strategy consisting of Support and Problem Solving (SPS), Cognitive-Behavioural Case Management (CBCM), and antidepressant medication. A secondary aim is to test biological and psychological variables that moderate and mediate response to this sequential treatment strategy.

4.4. Hypotheses

Hypothesis 1: In Step 1 we hypothesize that 50% of (500) initial participants will respond to the open label SPS intervention.

Hypothesis 2a: In Step 2 (n≥200), we hypothesize that there will be a significantly better response (on the primary outcome of functioning) to Cognitive Behavioural Case Management (CBCM) than to SPS.

Secondary hypotheses for Step 2 are as follows:

In the CBCM group, baseline cognitive biases and vulnerabilities will predict response and outcome (**H2b**), and those with better outcome and responders will show a significantly greater change on these variables than those with worse outcome (**H2c**) and non-responders (**H2d**).
Hypothesis 3: In Step 3 (estimated n=80), antidepressants will result in a significantly better outcome and greater response than placebo. The fast-fail subgroup is a naturalistic phase and no specific hypothesis is advanced.
Hypothesis 4: For the relapse prevention arm of the study extending over a period from 6 weeks after the start through to the end of the first year of the study, depending on the point of entry, we hypothesize that those receiving SPS will show a significantly reduced rate of relapse compared to the monitoring only group.

4.5. Study Objectives

4.5.1. Primary Objective

- To test the effect of a sequential treatment approach consisting of SPS/SPS and SPS/CBCM on functioning levels of UHR patients 6 months post baseline (end of Step 2), as measured using the Global Functioning: Social and Role Scales (138).

4.5.2. Secondary Objectives

- To test the effect of a sequential treatment approach consisting of SPS, CBCM and antidepressant medication on functioning levels of UHR patients 12 months from baseline (end of Step 3), as measured using the Global Functioning: Social and Role Scales.
- To test effect of a sequential treatment approach consisting of SPS, CBCM and antidepressant medication on transition to psychotic disorder by 12 months and 24 months from baseline, as assessed using the CAARMS (142).
- To test the effect of a sequential treatment approach on UHR status (maintenance versus remission) 1.5 months, 6 months, 12 and 24 months from baseline.
- To test the effect of a sequential treatment approach in UHR patients on level of psychiatric symptomatology, including positive psychotic symptoms, negative psychotic symptoms and depressive symptoms 6 months, 12 months and 24 months from baseline.
- To test relapse rates (to UHR+ status) in the relapse prevention/responder arm of the trial (SPS v monitoring) during the first 12 months.

4.5.3. Exploratory Objectives

The study aims to also explore whether there is a relationship between treatment response and specific patient profiles. In the subset of responders to a specific intervention, such as

CBCM or antidepressants, the study will examine whether it is likely that the treatment was effective due to the proposed putative mechanisms (cognitive biases and neuroprotection).

We will measure psychological variables at baseline, entry and exit from steps two and three and inflammatory, neuroendocrine and oxidative stress-related biomarkers, as well as antibodies to infectious agents and complement proteins at baseline, entry and exit from step three to test whether these variables moderate or mediate response to treatment.

It is expected that responders to CBCM will be those with more prominent cognitive biases and vulnerabilities at baseline, and which would improve in response to CBCM. The study will also investigate whether response to SPS or CBCM in Step 2 is mediate/moderated by degree of therapeutic alliance (quality of therapeutic relationship) between the participant and clinician. The study will also investigate whether CBCM and SSRIs have any impact on inflammatory and/or neuroendocrine, antibodies to infectious agents, complement proteins and oxidative stress biomarkers. Based on the background material reviewed above, we will also examine whether neurocognition, CSF complement proteins and genetic variables predict outcome (persistent attenuated psychotic symptoms, transition to psychosis, poor functioning) and moderate/mediate response to treatment. Since schizophrenia/psychosis and other deleterious clinical and/or functional outcomes (e.g., persistent severe mood and anxiety disorders) have a biological basis, in addition to psychosocial contributing factors, thorough investigation of potential biomarkers, informed by recent major breakthrough scientific findings (127) using cutting edge technologies, is highly warranted in a study of the present scope and scale. In the parents' study, we will also aim to explore the parent's experience of their child being identified to be at-risk for a major mental illness and their appreciation of autonomy in young people's risk identification and interventions. We will also explore their understanding of the balance between beneficence, risks and autonomy in the different STEP interventions and their preferences for future interventions.

5. Study Design

6.1 Description

This is a sequential multiple assignment randomized trial (SMART). Clients who are classified as being non-responders at the end of a Step will graduate to the next stage of intervention, while responders are offered entry to a maintenance/relapse prevention arm in which modest psychosocial care is compared in a randomised design with monitoring alone throughout the first 12 months of the study. A "fast fail" feature is included in step 3 if the participant deteriorates or fails to show improvement. This consists of dose enhancement and entry to a personal choice/shared decision-making treatment option.

Research Assistants conducting the clinical assessments will be blind to treatment allocation (Steps 2: single-blind). Step 3 of the study incorporates a randomised, double-blind, placebo-controlled stage of the study. Both participants and the study team will be blind to treatment (SSRI/placebo). The full study design is represented in Figure 1.

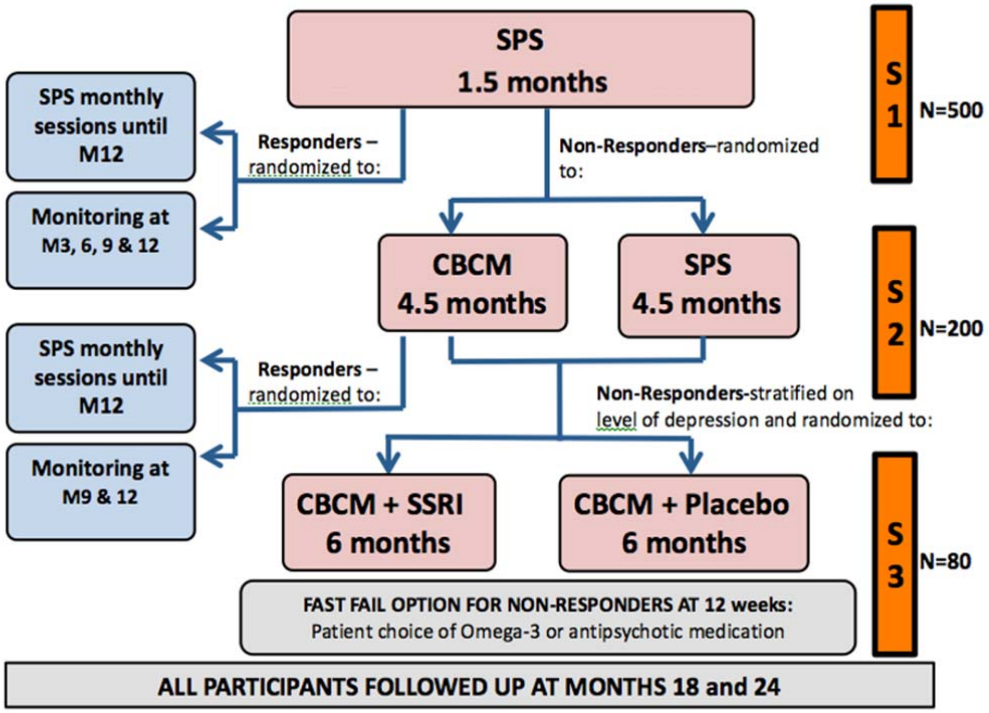

Figure 1. Study Design

Notes.

SPS = Support and Problem Solving.

CBCM = Cognitive Behavioural Case Management.

SSRI = Selective Serotonin Reuptake Inhibitor.

Rescue: At all stages, participants whose psychotic symptoms worsen to full threshold psychosis or who develop acute mania or sustained aggressive or suicidal risk will be discontinued from study treatment and treated according to clinician's choice, typically with antipsychotic or antidepressant medication.

Responders: Participants who no longer meet UHR criteria at the end of an intervention stage, as well as on at least one other assessment point, and an improvement of ≥ 5 points on the SOFAS or a SOFAS score of ≥ 70.

The design consists of 3 steps:

Step 1: Open-label Support & Problem Solving (SPS): "Open-label" SPS will be provided to all participants. Owing to the inherent difficulties associated with engaging this client group, there is no minimum number of sessions of SPS. Instead the SPS will be delivered as clinically indicated and practically possible within the 6-week period. We expect this initial

step will produce resolution of the current clinical phenotype in around 50% of participants, and thereby enrich the remaining sample for psychosis risk and functional impairment for randomisation in subsequent steps (57, 58). We also expect this to help retention of participants as drop outs have tended to occur early in previous UHR trials (93). Participants who respond to Step 1 (see below for definition of response) will be randomised to SPS monthly sessions v Monitoring at 3, 6, 9 and 12 months post entry to Step 1.

Monitoring will consist of the research assistant conducting a research assessment in addition to the clinician making contact with the participant, either by phone or in person, in order to monitor mental state and risk and to assess whether there has been any mental health deterioration which would indicate a need for change in clinical management (e.g., referral to a new service or need to re-engage with headspace or Orygen).

Step 2: CBCM vs SPS: Participants who do not respond (estimated at 50%) to Step 1 will be randomised to one of two treatment groups for 18 weeks (4.5 months): A more intensive psychosocial intervention (cognitive behavioural case management: CBCM) versus SPS. This design will test the potential benefit of a more intensive psychosocial intervention compared to extending the more basic intervention provided in Step 1 (SPS). It is not yet clear whether CBT, the primary component of CBCM, offers benefits over and above SPS. It is possible that non-responders to Step 1 (6 weeks of SPS) have simply not received the SPS intervention for long enough (i.e., the "dose" of SPS has not been sufficient to achieve symptom response and functional improvement). Extending SPS to a total of 6 months duration in Step 2 will test this possibility, as well as the potential benefit of introducing a more intensive psychotherapy in the form of CBCM. Research assistants conducting the research assessments will be blind to treatment allocation. This step will test an intervention that targets a key putative mechanism for psychosis vulnerability and risk: the psychological mechanism of dysfunctional cognitive biases.

Step 3 CBCM + SSRIs vs CBCM + Placebo: The estimated 50% who do not respond by the end of Step 2 (27, 56) will be randomised to antidepressant medication (fluoxetine: initial dose 20mg/day) or placebo, in addition to CBCM, for a further 6-month period. Randomisation will be stratified based on depression scores (MADRS total score <21 or ≥21). Previous UHR data at our service indicates that approximately half of recruited patients will score ≥21 on the MADRS. This stage will test whether antidepressant medication in addition to CBCM contributes to improving the outcome of those who have failed to respond to previous interventions. This stage of the study incorporates a randomised, double-blind approach with both the participants and study teams members being blinded to treatment allocation. Antidepressants have previously shown potential but not conclusive benefits for UHR patients. The dose of SSRI will be titrated up at 6 weeks from 20 mg to 40 mg daily (one to two capsules of matching placebo) if there is no response based on clinical judgment of the

treating doctor. It is appropriate to reserve antidepressants for this third step in view of their higher risk of adverse effects. They may be effective either immediately or in terms of risk reduction for psychosis or other non-psychotic syndromes via an effect on depression or anxiety or via a neuroprotective route (140). By stratifying by depression at this stage of randomisation, we will be able to determine the impact of antidepressant medication on outcome in this population in relation to intensity of depressive symptoms, which will have direct implications for clinical decision-making as well as shed light on the possible mechanisms underlying the benefit of antidepressant medication for this patient group (i.e., as neuroprotective or an indirect pathway via improvement of mood disturbance).

“Fast fail” in Step 3: Step 3 will incorporate a “fast fail” feature such that if patients deteriorate or have not responded after 12 weeks of this third step of the study, they will be offered the option of:

1. Continuing as randomised.
2. Increasing the dosage of antidepressant medication/placebo to 40mg if this has not already been done.
3. Continuing the antidepressant or placebo with the addition of Omega-3 fatty acids or low dose antipsychotic medication chosen via a shared decision making approach, supported by accessible information on the pros and cons of this decision.

Non-responders: All participants who do not meet the ‘response’ criteria below (and who have not yet made a transition to first episode psychosis or mania).

Responders: All responders during Steps 1 and 2 will be randomized at the end of each step to monthly SPS sessions or simple monitoring at 3 monthly intervals (Response to Step 1; 6, 9 and 12 months; Response to Step 2: 9 and 12 months) to assess whether such responses can be sustained. All relapsing participants at any stage and all non-responders at the 12 month point will be reassessed and offered “treatment as usual” via **headspace** and Orygen. In year 2 all participants will be clinically monitored and receive research follow up at 6-month intervals (18 and 24 months).

Rescue: Finally, a safety net or “rescue” strategy is built into the study design from Step 1 through to the end of Step 3 where participants whose psychotic symptoms worsen to the point of full threshold psychosis, or who develop acute mania, or sustained aggressive or suicidal risk as defined using the CAARMS are discontinued from study treatment and treated according to clinician’s choice, typically with antipsychotic or antidepressant medication, either as inpatients or with intensive community care and with full evidence-based psychosocial care (see Section 7.8).

Moderators and mediators of response: In addition to testing the efficacy of the treatments in Steps 2 and 3 we will be exploring as a secondary aim whether there is a relationship between response and specific forms of treatment. Hence in the responders to a specific intervention such as CBCM or antidepressants, we will address whether the treatment is likely to be working through the putative mechanism proposed. We will measure psychological variables, inflammatory and oxidative stress, antibodies to infectious agents, complement proteins and immunoglobins, and related biomarkers at baseline, entry and exit from steps two and three. We expect that responders to CBT will be those with more prominent cognitive biases and vulnerabilities at baseline, and which would improve in responders to CBT. We will also examine: i) whether CBCM has any impact on inflammatory and/or oxidative stress biomarkers, as well as antibodies to infectious agents, complement proteins and immunoglobins, ii) whether neurocognition and genetic variables predict outcome (persistent attenuated psychotic symptoms, transition to psychosis, poor functioning) and moderate/mediate response to treatment (e.g., whether COMT Val158Met polymorphism influences CBCM response).

Response: The definition of response is as follow:

- The Global Rating Scale or the Frequency score need to be less than 3 for all of the four positive symptoms (UTC, NBI, PA and DS) and
- There is an improvement of at least 5 points in SOFAS compared with baseline or the SOFAS score is at least 70.

The response status will be determined using the “current” ratings of CAARMS and will be determined at weeks 4, 6, 12, 24, 36, 52. These current ratings are based on the patients’ experience in ‘the last fortnight’ and are for the four positive symptoms only.

The definitions for a responder at the different time points are as follow:

- A responder at the end of Step 1 is one who shows a response at both weeks 4 and 6.
- A responder at the end of Step 2 is one who shows a response at both weeks 12 and 24.
- A responder at week 36 is one who shows a response at week 36 – this is for the fast fail option.

Key:
UTC = Unusual Thought Content subscale of the CAARMS
NBI = Non-Bizarre Ideas subscale of the CAARMS
PA = Perceptual Abnormalities subscale of the CAARMS
DS = Disorganised Speech subscale of the CAARMS
SOFAS = Social and Occupational Functioning Assessment Scale

Non-response: All participants who do not meet the ‘response’ criteria (and who have not yet made a transition to psychosis or mania).

All participants will be closely monitored for adverse events and concomitant medication use throughout the study. Vital sign measurements, and clinical laboratory evaluations will be performed at predefined time points throughout the study as shown in the Schedule of Assessments in Section 4.

Parents' sub study (EXPECT): We propose to conduct 25 interviews with parents of young people (12-18 yrs) who have already consented to STEP. After full informed consent (and assent from young people), the researchers will gather specific information from parents with semi-structured interview prompts Information about probability of risk is assessed using prompts from the semi-structured interview developed by Erickson and colleagues (2014) (162). Interviews will be conducted by two trained medical students under the supervision of Dr Ratheesh and Dr Bendall. The study is expected to be conducted between Feb and May 2019.

5.1. Study Setting

The sites for the SMART trial will include four (4) youth mental health services (**headspace** centres) funded through the Commonwealth Government of Australia. These centres, located in purpose-built premises in shopping and community precincts in the suburbs of Sunshine, Glenroy, Werribee and Craigieburn, in the North Western Melbourne metropolitan region, provide universal access under a Federally-funded model of enhanced primary care, to a broad array of mental health and welfare services for 12-25 year olds (inclusive) in a youth friendly, stigma-free "one stop shop" environment. These **headspace** recruitment sites are operated under contract to **headspace** nationally by Orygen of which the Principal Investigator Pat McGorry is Executive Director, and they have already been the platform for several successful clinical trials and cohort studies. From 2014, over 5000 young people will be seen at these 4 clinics per annum. The study will also recruit from the PACE clinic, a specialised UHR research clinic that has two decades of innovation, clinical and research experience with this group of patients and their families (22, 134, 142).

5.2. Study Endpoints

5.2.1. Primary Endpoint

The primary endpoint is 6 months post-baseline (end of Step 2). The study will test the effect of the sequential treatment approach of SPS and CBCM on functioning levels at this time point. Functioning will be measured using the Global Functioning: Social and Role Scales (135).

5.2.2. Secondary Endpoint(s)

- Functioning levels 12 months from baseline (end of Step 3), as measured using the Global Functioning: Social and Role Scales.
- Transition to psychotic disorder by 12 months and 24 months from baseline, as assessed using the CAARMS (142).
- UHR status (maintenance versus remission) 1.5 months, 6 months, 12 and 24 months into treatment as determined by the CAARMS.
- Psychiatric symptomatology, including positive psychotic symptoms, negative psychotic symptoms and depressive symptoms 1.5 months, 6 months, 12 months and 24 months into treatment assessed using the CAARMS, BPRS, SANS and MADRS.
- Relapse rates (i.e., relapse to UHR+ status) in the relapse prevention/responder arm of the trial (SPS v monitoring) during the first 12 months.

5.3. Study Duration

The controlled intervention component of the study is of 12 months duration. Participants will be followed up with research assessments up to 24 months post-baseline.

5.4. Study Risk Assessment

Participants in the active treatment arm of the study will have regular (ideally weekly-fortnightly) contact with their treating team (case managers and/or psychiatrist), during which risk will be monitored. Participants in the non-active treatment arm will either have monthly contact with their treating team or undergo 3-monthly monitoring. If participants are deemed to be at substantial risk to themselves or others (see definition in 9.8.2) they will be discontinued from the study and receive required clinical care. These participants will continue to be followed up with research assessments.

6. Participant Population

6.1. Description

Male and female participants aged between 12 and 25 (inclusive) who meet “ultra high risk” for psychosis criteria at entry to services as determined using the CAARMS.

6.2. Sample Size and Power

The study is powered on the primary aim of comparing six-month functional outcome between the two treatment groups in Step 2. Previous data collected at the PACE clinic indicates that approximately 50% of UHR participants remain UHR+ 6 weeks into treatment (i.e., will be classified as “non-responders” to Step 1 intervention). Assuming a 50% non-response rate in Step 1, an entry sample size of 500, a drop-out rate of 20% and hence a step

2 sample size of 200, we will be able to detect a fairly small effect size (0.18 in a parallel cell design) with a power of 80% and significance level of .05 by the end of Step 2. Approximately 5000 young people per annum will be attending **headspace** centres from 2014. Recent data indicates that approximately 38% of young people seen at **headspace** meet UHR criteria, which means that approximately 2000 patients per annum will be eligible for the study. A two-year recruitment period means that 500 participants would need to be recruited from a pool of 4000 patients (12.5%). This recruitment rate is highly realistic given that recruitment rates to our previous UHR intervention trials have been approximately 30% of eligible patients (143). It is standard for SMART trials to be powered on a single stage or aspect of a multi-staged design (127). Nevertheless, assuming 50% non-response rate in Step 2 and 20% drop-out rate will result in n=80 in Step 3 which will be sufficient to detect a clinically important medium effect size (0.29) for comparing the two interventions with a power of 80% and significance level of .05.

6.3. Participant Selection Record

Investigators are to keep a record of participants who were screened for the study but were not enrolled.

6.4. General Considerations for Enrolment

Only participants who meet all of the inclusion and none of the exclusion criteria will be eligible to participate in the study. Applications for individual protocol waivers will be considered on a case-by-case basis.

6.5. Inclusion Criteria

A participant will be considered eligible for inclusion in this study only if all of the following criteria apply:

1. Age 12 – 25 years (inclusive) at entry
2. Ability to speak adequate English (for assessment purposes)
3. Ability to provide informed consent. *Where participants are minors (i.e. have not reached the age of eighteen), consent will also be obtained from one of the participant's parents or legal guardian. Both the parent/legal guardian and participant will be required to sign a consent form in such a case. It will be the treating doctor's decision made in consultation with the investigator to determine whether a participant who is a mature minor has the capacity and competence to consent to the study.*
4. Meeting one or more UHR for psychosis groups (as defined in Table 1).

Young people seeking help via **headspace** centres are routinely screened using the HEADSS

assessment, and as clinically appropriate, an additional screening tool will be used by clinicians which will help them determine the UHR status of their clients. This tool is the Prodromal Questionnaire-16 (PQ-16)(144). Those who score 6 or more positive symptoms on the PQ-16 will be assessed using the CAARMS(142) and SOFAS(148) to determine UHR status. A cut-off score of 6 on the PQ-16 screening tool has previously been found to identify UHR+ cases with high sensitivity (87%) and specificity (87%) (144) and has also been found to identify a more enriched sample for psychosis risk (146). This will not apply at the PACE clinic as all clients have already been assessed as UHR + through clinician administered CAARMS (as part of standard clinical entry).

Table 1: Operationalised UHR intake criteria

---

1. Group 1: Vulnerability Group

Family history of psychosis in first degree relative OR Schizotypal Personality Disorder (as defined by DSM IV) in identified patient

AND

Drop in Functioning:

Recency: Change in functioning occurred within last year

Impact: SOFAS score at least 30% below previous level of functioning and sustained for at least one month.

OR

Sustained low functioning:

Recency: For the past 12 months or longer

Impact: SOFAS score of 50 or less.

2. Group 2: Attenuated Psychotic Symptoms Group

2a) Subthreshold intensity:

Intensity: Global Rating Scale Score of 3-5 on *Unusual Thought Content* subscale, 3-5 on *Non-Bizarre Ideas* subscale, 3-4 on *Perceptual Abnormalities* subscale and/or 4-5 on *Disorganised Speech* subscales of the CAARMS

Frequency: Frequency Scale Score of 3-6 on *Unusual Thought Content*, *Non-Bizarre Ideas, Perceptual Abnormalities* and/or *Disorganised Speech* subscales of the CAARMS

Duration: symptoms present for at least one week

<u>Recency:</u> symptoms present in past year

2b) Subthreshold frequency:

<u>Intensity:</u> Global Rating Scale Score of 6 on *Unusual Thought Content* subscale, 6 on *Non-Bizarre Ideas* subscale, 5-6 on *Perceptual Abnormalities* subscale and/or 6 on *Disorganised Speech* subscales of the CAARMS

<u>Frequency</u>: Frequency Scale Score of 3 on *Unusual Thought Content*, *Non-Bizarre Ideas,*, *Perceptual Abnormalities* and/or *Disorganised Speech* subscales of the CAARMS

<u>Recency:</u> symptoms present in past year

3. <u>Group 3</u>: BLIPS Group

<u>Intensity:</u> Global Rating Scale Score of 6 on *Unusual Thought Content* subscale, 6 on *Non-Bizarre Ideas* subscale, 5 or 6 on *Perceptual Abnormalities* subscale and/or 6 on *Disorganised Speech* subscales of the CAARMS

<u>Frequency</u>: Frequency Scale Score of 4-6 on *Unusual Thought Content*, *Non-Bizarre Ideas, Perceptual Abnormalities* and/or *Disorganised Speech* subscales

<u>Duration</u>: Symptoms present for less than one week and spontaneously remit on every occasion.

<u>Recency</u>: symptoms present in past year

6.6. Exclusion Criteria

Potential participants who meet any of the following criteria will not be eligible for participation in this study. Applications for individual protocol waivers will be considered on a case-by-case basis as detailed in Section 6.4.

Exclusion criteria consist of:

1. Past history of a psychotic episode of one week or longer, whether treated with antipsychotic medications or not.
2. Attenuated psychotic symptoms only present during acute intoxication.
3. Organic brain disease known to cause psychotic symptoms, e.g. temporal lobe epilepsy.
4. Any metabolic, endocrine or other physical illness, e.g. thyroid disease, with known neuropsychiatric consequences.
5. Diagnosis of a serious developmental disorder, e.g. Severe Autism Spectrum Disorder.
6. A documented history of developmental delay or intellectual disability.
7. Previous or current SCID diagnosis of bipolar disorder I.

Previous or current use of antipsychotic or antidepressant medication is not an exclusion criterion. In the case of current antipsychotic or antidepressant use, we will taper and cease this at entry to the study. This is consistent with clinical guidelines because antipsychotics are not indicated for the UHR population (147, 148) and antidepressants in young people are recommended only for those with severe depression, with psychosocial interventions recommended for those with mild-moderate depression (149). Most UHR cases who suffer from depression have mild-moderate rather than severe depression (143, 150). If they develop severe depression during the course of the study they are likely to meet the discontinuation criterion of risk to self and be commenced on antidepressant medication.

6.7. Participants Restrictions

In Steps 1 and 2 antidepressants will not be permitted. The use of antipsychotics or mood stabilisers is not permitted for the duration of the study (24 months).

6.8. Discontinuation and Withdrawal

6.8.1. Voluntary discontinuation or withdrawal

The term withdrawal, as it is used in this protocol, is defined as a participant and/or parent/guardian’s retraction of consent to participate in the study. All other events whereby a participant ceases participation in study treatment, whether continuing research follow up interviews or not, are categorised as discontinuation. Participants are free to discontinue or withdraw their participation in the study at any time, without prejudice to further treatment. Participants who discontinue or withdraw from the study should always be asked about the reason(s) for their withdrawal and about the presence of any adverse events. If possible, they should be seen and assessed by an Investigator and complete the End of Study Assessments as shown in Section 3. Adverse events should be followed up until resolution, the adverse event stabilises or the participant is lost to follow up.

6.8.2. Study Discontinuation Criteria

The study may be terminated at any time by the Sponsor or by a decision of the Data Safety Monitoring Board.

6.8.3. Participant Discontinuation Criteria

A participant will be considered ‘discontinued’ from the study in cases where the study treatment is ceased. If appropriate, participants should be invited to continue in the study assessments in accordance with the study schedule, even though they are no longer receiving study treatment.

A participant will be discontinued from the study intervention in the following situations:

1. Serious adverse events at Investigator discretion.

2. Pregnancy or lactation during Step 3 (which involves antidepressant medication and fast fail).

3. Severe non-compliance to protocol as judged by the Investigator.

4. At the Investigator's discretion.

After consultation between the treating doctor and the study investigators, participant will also be discontinued from the study in the case of:

5. Transition to psychosis or other deterioration requiring a change of therapy: Participants who are assessed as having transitioned to first episode psychosis or mania. Transition is operationally defined via the CAARMS, as in previous studies (142), as positive psychotic symptoms occurring every day for one week or longer. A transition to full threshold mania will also mean that the participant is stopped since mood stabilisers will be administered. Establishing whether a STEP participant has transitioned to first-episode psychosis (FEP) will be determined by the research team in collaboration with the clinical team (where necessary). When a participant is thought to have transitioned to psychosis, it is important that we determine this by conducting a post-transition assessment. This will enable us to confirm that the participant has definitely transitioned to FEP and it will also allow us to derive the appropriate psychotic diagnosis. As functioning is our main outcome measure, we will also administer the Cornblatt Social and Role Functioning measures.

Hence, the research assistant will conduct the following assessments as close to the transition date as possible:

- CAARMS positive symptoms
- Cornblatt Social and Role Functioning measures (GF S & R)
- SCID V - Mood module (the relevant sections to derive a psychotic mood diagnosis) and the Psychosis module

46. Participants who have sustained risky levels of aggression or suicidality associated with their psychotic symptoms (or otherwise) at one of the follow up assessments. Sustained levels of aggression is defined as a CAARMS 5.4 scale score of 6 sustained over the past 2 weeks and sustained levels of suicidality as a CAARMS 7.3 scale score of 6 sustained over the past 2 weeks.

These cases may be offered antipsychotic or antidepressant medication as inpatients or via intensive community care, as per early psychosis guidelines (147, 148). This constitutes rescue treatment.

6.9. Replacement of Participants

Participants who are withdrawn or discontinued from the study will not be replaced.

6.10. Incorrectly enrolled Participants

Incorrectly enrolled participants will be discussed by the Investigators on a case-by-case basis, and a written decision will be made as to whether they should remain on-study or be discontinued. Incorrectly enrolled participants will constitute a protocol violation and will be reported to the approving ethics committee.

6.11. Recruitment

The **headspace** centres, located in purpose-built premises in shopping and community precincts in the suburbs of Sunshine, Glenroy, Werribee and Craigieburn, in the North Western Melbourne metropolitan region, provide universal access under a Federally-funded model of enhanced primary care, to a broad array of mental health and welfare services for 12-25 year olds (inclusive) in a youth friendly, stigma-free “one stop shop” environment. These **headspace** recruitment sites are operated under contract to **headspace** nationally by Orygen of which the Principal Investigator Pat McGorry is Executive Director, and they have already been the platform for several successful clinical trials and cohort studies. From 2014, over 5000 young people will be seen at these 4 clinics per annum. Recent data indicates that approximately 38% of young people seen at **headspace** meet UHR criteria, which means that approximately 2000 patients per annum will be eligible for the study. A two-year recruitment period means that 500 participants would need to be recruited from a pool of 4000 patients (12.5%). This recruitment rate is highly realistic given that recruitment rates to our previous UHR intervention trials have been approximately 30% of eligible patients (143). It is standard for SMART trials to be powered on a single stage or aspect of a multi-staged design (131). The study will also recruit from the Orygen Youth Health Clinical Program’s PACE clinic, a specialised UHR research clinic that has two decades of innovation, clinical and research experience with this group of patients and their families (22, 141, 142). The PACE clinic provides clinical service to approximately 100 UHR cases/year.

Orygen has recently completed recruitment to an international multi-centre omega-3 fatty acid intervention study in the UHR population (‘Neurapro’) (25). Orygen successfully recruited 304 participants to this trial, currently in the follow up phase. Orygen are also currently recruiting to a large double-blind randomized clinical trial (n=300) examining the use of aspirin in the treatment of depression in young people. Both trials show a 30-40%

recruitment rate of eligible participants, which indicates the strong feasibility of recruiting the required sample size to the proposed study, particularly given the similar interventions used in this study and the extra clinical platforms from which to recruit (the additional **headspace** centres).

Potential Problems and Alternative Strategies: The study Sponsor, Orygen, does not anticipate difficulty recruiting the required sample size given the large pool of patients from which to recruit, as outlined above, and the high percentage (~40%) who meet UHR criteria. However, if there are difficulties with achieving target recruitment rates the option of recruiting through existing collaborations with other research-capable **headspace** centres is available. Orygen has successfully used a range of retention and adherence strategies in previous UHR intervention studies, which we will also implement in the proposed study. These include: outreach capacity for clinical and research contacts, youth-friendly clinic settings, flexibility of appointment times and locations, reminder systems for appointments and medications, and modest but adequate financial compensation for travel and for participants' time in attending research visits. The Sponsor also notes that even if the study does not yield statistically significant results on the primary outcomes it will produce a rich data set in which to examine baseline predictors of treatment response, effective dosages of the trial medications, contribute to understanding of mechanisms underlying illness evolution (e.g. if findings indicate inflammatory markers reduce with treatment but there is no corresponding symptom change), and inform the design of future trials and clinical treatment strategies in this population.

Recruitment of parents into the qualitative study:

This side-study will use the following steps to identify eligible parents.

a) RA will identify suitable young people based on the study tracking sheet and approach young people,

b) if the young person provides assent, this is documented in the trial file/clinical file,

c) then the recruiting medical student calls the parent using the contact number that the young person provides,

d) if the parent is interested, the student will then mail/email the consent form to the parents,

e) if the parents agree, they are invited to attend the Parkville clinical sites for full informed consent and to complete the interview,

f) students to feedback to RAs re: assessment, consent or declined consent so this can be tracked, and young people informed as necessary.

7. Visits and Assessments

The Schedule of Assessments is shown in Section 4 *Schedule of Assessments and Endpoint Measures*.

There are to be 11 outpatient visits throughout the study. Participants will be assessed at screening, baseline, 1 month (W 4), 1.5 months (W 6 start Step 2), 3 months (W 12), 6 months (W 24 end Step 2), week 25, 9 months (W 36), 12 months (W 52 end Step 3), 18 months (W 78) and at the End of Study Visit 24 months (W 104). If a visit must be split over two or more dates due to scheduling requirements or participant considerations all study assessments must be performed within the original visit window.

Screening and Baseline
Informed consent, inclusion/exclusion criteria review (baseline only) and demographics will be conducted during the screening visit. The medical and psychiatric history will be captured and a physical examination will be completed during medical review by the study doctor before the end of Step 1.

Psychopathology Scales
The full suite of psychopathology scales will be administered at baseline. An abridged suite of psychopathology scales will be administered at M1.5 (W 6), M6 (W 26), M9 (W 36), M12 (W 52), M18 (W 78) and M24 (W 104). The CAARMS will be administered at all visits with only the positive symptoms scale utilised at screening.

Functioning and Quality of Life
The SOFAS will be administered at screening, baseline, M1 (W4), M1.5 (W 6), M3 (W 12), M6 (W 24), M9 (W 36), M12 (W 52), M18 (W 74), and M24 (W 104).

AQoL-8D will be administered at baseline, M3 (W 12), M6 (W 24), M9 (W 36), M12 (W 52), M18 (W 74), and M24 (W 104).

Global Functioning will be administered at baseline, M6, M12 (W 52), M18 (W 78) and M24 (W 104).

Safety and Medical Review
Adverse events and concomitant medications review will be conducted at every visit with safety bloods (clinical bloods; haematology and biochemistry panels) conducted prior to Step 3 at week 25, and also at the end of Step 3 at week 52.

In order to assess possible deleterious effects associated with the psychotherapy, a self-report (“Effect of Psychological Therapy Scale”) developed by our research team will be

given to the client at months 12 and 24. This 2-item self-report questionnaire will be used to assess whether the client feels that they experienced any negative or positive effects from the psychological therapy.

Medical Review

All clients will undergo a medical review before the end of Step 1. Additionally, during the standard weekly clinical reviews held across all the sites, both clinicians and research assistants will present and discuss current clients with the study doctor. Based on this review process, the study doctor will exercise their clinical judgement to decide when a client requires urgent medical review and will liaise with site and study staff to see the client accordingly. After the M12 Intervention period participants will also have the opportunity to be reviewed by the STEP Study Doctor from M13 onwards, as needed. This is not included as a study assessment but rather an appointment available to the young person to assist in their ongoing clinical management and also their engagement in the STEP study, prior to their follow up interviews. This appointment is not compulsory but all participants will be informed of this opportunity at their M12 assessment and given the choice to schedule in this appointment for the following month. Future appointments after M13 with the Study Doctor may be booked in as required.

Other

- Neurocognition (optional) will be conducted at baseline, 6M (W24) and M12 (W 52).
- Research bloods will be collected at baseline, M6 (W 25) and M12 (W 52).
- The DNA sample (15ml of blood or a sample of saliva will be collected at baseline. CSF (optional) for research and genetic analyses will be conducted during Step 2 (approximately Week 18), and prior to Week 52. Prior to the lumbar puncture being performed, routine clinical safety bloods will be collected and reviewed to ensure the participant's blood clots normally. Participants will also be required to have had a brain Magnetic Resonance Imaging (MRI) scan in the 6-12 months prior to the first Lumbar Puncture to exclude an intracranial mass lesion, hydrocephalus, or any other structural contraindication to having a Lumbar Puncture performed.
- Hair cortisol samples will be collected at baseline, W12, W25 and W52.
- Therapeutic alliance scales (Working Alliance Inventory) will be completed by both the client and the clinician at M6 (Week 24) and M12 (Week 52). The purpose of including these scales is to investigate whether the effect of psychological treatment is moderated/mediated by the degree of therapeutic alliance (quality of relationship) between client and clinician and to measure changes in therapeutic alliance over time.
- If a participant is lost to follow-up or discontinued from the study treatment for a reason other than revoking their informed consent, they will also be followed up with assessments

conducted at all time-points where possible.

- If a client has transitioned to psychosis, a post-transition assessment will be conducted comprising the positive symptoms of the CAARMS to confirm transition status; the psychosis and mood sections of the SCID; and the Global Functioning Social & Role Scales.
- If a parent consents to the EXPECT sub-study, the parent will be interviewed with regards to their understanding of the magnitude of risk, and the ethical implications of risk identification in their young person.

7.1. Study Measurements and Procedures

Measures of risk factors for treatment response and non-response will consist of variables demonstrated or thought to influence treatment response and transition to psychotic disorder. These will include: demographics (age, gender, socioeconomic status, urbanicity, educational attainment); premorbid functioning; psychiatric symptoms and syndromes; cognitive biases; biological variables (inflammatory markers, oxidative stress measures, cell membrane lipids); neurocognitive variables (processing speed, verbal memory, social perception, working memory, fluid reasoning); dietary factors and family history of psychiatric disorder.

In addition, information on the following possible confounders will be collected: treatment adherence, number and content of SPS and CBCM sessions (via audiotaping), concomitant medication, other psychiatric treatments and self-reported substance use.

7.1.1. Clinical Laboratory Tests

Clinical laboratory tests of blood samples (assessed at W25, M12, and prior to lumbar puncture for those consenting to the CSF collection) will consist of: U&E, FBE, LFTs, TFTs, cholesterol and triglycerides; serum lipids (HDL and LDL). As appropriate, the clinical laboratory test for female participants will also include an hCG test.

7.1.2. Participant Self Reports

- Davos Assessment of Cognitive Biases Scale (DACOBS) (150): The DACOBS will used to assess cognitive biases. This will be administered at baseline, W6, W24, and M12.
- Childhood Trauma Questionnaire (CTQ)(152): This 25-item self-report questionnaire will be used to measure childhood maltreatment. This will be administered at baseline.

7.1.3. Rating Scales

Clinical scales:

- Comprehensive Assessment of At Risk Mental States (CAARMS) (142): The CAARMS will be used to assess UHR status, prodromal symptoms, and transition to psychotic

disorder. The CAARMS is a validated and widely used instrument in prodromal research.

- Social and Occupational Functioning Scale (SOFAS)(148): The SOFAS will be used to assess UHR status and functioning.
- SCID-5 (156): the SCID-5 will be used to assess DSM diagnoses.
- Brief Psychiatric Rating Scale (BPRS)(157): The BPRS will be used assess psychiatric symptomatology.
- Scale for the Assessment of Negative Symptoms (SANS)(158): The SANS will be used to assess negative psychotic symptoms.
- Montgomery Asberg Depression Rating Scale (MADRS)(151): The MADRS will be used to assess depressive symptoms.
- Global Functioning: Social and Role Scales (GF: S and GF: R): The GF: S and GF: R will be used to assess functioning.
- AQoL-8D (Mental Health): The AQoL-8D instrument will be used to assess quality of life.
- SCID-5: The SCID-5 will be used to assess psychiatric symptoms and syndromes.
- SCID-II PD: The SCID-II PD will be used to assess for symptoms of schizotypal and borderline personality disorders.
- Family History Index: The Family History Index will be used to assess family history of psychiatric disorder
- ASSIST (152): The ASSIST will be used to assess substance use.
- Short Questionnaire for the Assessment of Anomalous Self-Experience (SQUEASE(153)): This 11-item instrument will assess disturbance of basic self-experience.
- Side effects and adverse events will be recorded using the adverse event tracking log.
- Demographics and general medical history will be assessed at baseline.
- Working Alliance Inventory (Client and clinician self-report) will be administered at 6 months and 12 months.
- Psychological Therapy Side Effect Scale will be administered at 12 months and 24 months.

7.1.4. Neurocognitive Assessments

- The neurocognitive assessments are being conducted at three time points: Baseline, 6 M and 12 M. The follow up assessments at 6 M and 12 M are being included to enable us to assess the change over time i.e. difference or lack of difference between Baseline and follow up assessment.

- Bell-Lysaker Emotion Recognition Task (BLERT): Measuring ability to correctly identify emotional states including happiness, sadness, fear, disgust, surprise, anger, or no emotion.
- Brief Assessment of Cognition (BAC): Assessing verbal memory and learning, working memory, motor function, verbal fluency, speed of processing, and executive function.

7.1.5. Research Bloods

The biological variables to be assessed will include: inflammatory markers (i.e., cytokines including BDNF, TNFα, IL-1A, IL-1β IL-6, and IL-10), oxidative stress (i.e., thiobarbituric acidic reactive substances, nitric oxide activity, superoxide dismutase), and lipid metabolism (i.e., fluorometric assessment of PLA_2 activity; mass spectrometry of cell membrane fatty acids). Approximately 20-30 mL of blood must be collected at each time point in the morning before breakfast between 8 am and 10 am. The blood samples will be stored frozen locally for batched analysis.

Biomarker analysis capabilities are in place via established collaborations with the University of Jena (PLA_2), the University of Wollongong (lipids), the University of Queensland, the University of Adelaide (oxidative stress and inflammatory markers), Royal College of Surgeons, Ireland (proteomic and cytokine analyses), and the John Hopkins School of Medicine (antibodies to infectious agents). As technology in this area advances we may also analyse these samples collected for as yet unidentified analytes.

7.1.6. Genetic Sample (Blood or Saliva)

A whole blood sample (15 ml) or spit pot sample will be collected to prepare DNA for genomic analyses. The genetic analyses will be of genes for research interest only. The genes investigated will not be diagnostic and have no relevance for participants' current or future health, thus participants will not require any pre−test counseling.

Further specific procedural information related to collecting, processing & shipping the blood samples will be included in the STEP *Study Procedures and Pathology Manual.*

Samples of blood or saliva and any relevant clinical data (for example symptomatology or diagnosis) obtained for the purpose of this research project will be transferred to central

storage facilities, e.g. at The Broad Institute and at an appropriate local Australian research and analytical facility.

7.1.7. Vital Signs, Height and Weight/Physical Examination

Vital signs will be measured electronically and will consist of measurements for heart rate, respiratory rate and blood pressure.

Height will be measured by calibrated stadiometers and weight measured in kilograms using a calibrated electronic scale. The height and weight will be used to calculate the body mass index (BMI). In addition, the waist circumference will be measured (in centimetres) on these occasions.

7.1.8. Cerebrospinal Fluid Collection

CSF levels to be assessed include C1q, C2, C3, and C4 proteins by enzyme-linked immunosorbent assay (ELISA) is a plate-based assay technique designed for detecting and quantifying substances such as peptides, proteins, antibodies and hormones. ELISAs will be employed in STEP to measure CSF proteins of neuronal injury and neuronal dendritic spine function and neuronal structure as well as using metabolomics and proteomics to measure CSF small molecule metabolites and proteins. As technology in this area advances we may also analyses these samples collected for as yet unidentified analytes.

The CSF collection will be made via a lumbar puncture which will be performed by a neurologist in The Department of Medicine, The Royal Melbourne Hospital, Parkville. We have consulted extensively with our colleagues, Prof Terence O'Brien and Prof Dennis Velakoulis, who are experienced in this procedure. There will be less than 6 ml of CSF collected during the lumbar puncture. Routine pathology screening will be conducted on the CSF and the sample used for research purposes will be prepared in accordance with the STEP Study Procedures Manual. The genetic analysis will be for genes of research interest only. The genes investigated will not be diagnostic and have no relevance for participants' current or future health, thus participants will not require any pre−test counseling. Patients will be supported fully during this process, which in young people is technically a simpler, less onerous procedure. Patients will be able to rest for an appropriate period afterwards to reduce the risks of the most common adverse effect which is headache. If the latter does occur, it will be responded to rapidly with analgesia.

7.1.9. Hair Cortisol Sample

A hair sample will be collected and analysed for free cortisol concentrations as a marker of chronic stress. Cortisol analysed from human hair provides a mirror image of cumulative cortisol concentrations (Kirschbaum, C. et al. 2009, PNEC 34:32-37). Consequently, hair samples will be collected at baseline (week 0) and week 12, 25 and 52 along with other

biochemical assessments. The rationale for conducting cortisol assays is to monitor the effects of the stepwise interventions on HPA-axis activity, and to examine the relationship between HPA-activity and clinical outcomes.

Approximately 10mm^2 (the diameter of a pencil) of hair will be collected from the posterior vertex region, stored in aluminium foil and a zip-lock bag with the proximal end marked. The proximal 3cm of the sample analysed for cortisol concentrations with a commercially available high sensitivity ELISA kit (e.g. Salimetrics™, California, USA). Processing and analysis of the hair samples will be carried out at the Laboratory of Psychiatric Neuroscience at James Cook University, Townsville. To gather relevant covariates for hair steroid analysis (such as hair colour, hair treatments), a brief screening interview will be conducted.

7.1.10. Parents' assessment in the EXPECT sub-study

After obtaining consent, parents will be asked questions regarding themselves in general and then questions that will orient the parent to the young person's problems. After this, they will be asked about their experience of allowing their young person to be in a research study (consent process). They will then be asked about their understanding of the magnitude of their young person developing future mental disorders. If they indicate that they are aware of a higher level of risk, they will then be asked about their experience of coming to be aware of that risk (exploring benefits and risks of risk identification). Parents will then be asked about whether young people should be told about a higher risk, if that was the case (autonomy). The interviews will finish with further orienting questions about interventions that young people receive. After this, they will be asked to complete another short paper and pen survey regarding the types of interventions that they would believe are appropriate for their young person, were they to be at risk for future mental disorders. The assessment will conclude with parents being provided the scheduled reimbursements, clarifying any of their questions, and a short assessment of their level of distress and psychological needs. The interviewer will discuss referral options and other options for support if necessary. The parents will also be offered a follow-up phone call to discuss any concerns within 2-4 weeks of completion of the interview.

8. Study Intervention

8.1. Study Interventions: Doses, Administration and Treatment Regimen

Support and Problem Solving (SPS)

SPS consists of basic supportive counseling and problem solving strategies (e.g., problem solving difficulties with school, housing, interpersonal relationships, etc.) delivered within a positive psychology framework. This is a minimal-level treatment as usual intervention for this help-seeking group. Sessions will be ideally weekly to fortnightly and of 30-50 minutes duration. Formal psychotherapy is not included in this intervention. A recent RCT conducted at our UHR clinic in Melbourne indicated that this form of intervention may be as effective as CBT and low-dose antipsychotics in providing symptomatic and functional improvement, and reducing transition risk, at least over the short term (93, 94). Furthermore, this simple and brief type of approach has been proven safe, engaging and effective for the wider range of early diagnostic presentations in our primary care platform, **headspace** (159).

Cognitive-Behavioural Case Management (CBCM)

CBCM consists of CBT embedded within case management, as successfully developed and implemented in our recent multi-centre omega-3 fatty acid intervention study (‘Neurapro’, http://www.anzctr.org.au/)(25). Full intensity CBT is delivered consisting of modules addressing attenuated psychotic symptoms, stress management, negative symptoms/depression, and other comorbidity (anxiety disorders, etc.). There is preliminary evidence (see 6.1) that yoga and mindfulness may be of value in early psychosis patients and have therefore been incorporated into the current treatment package, delivered in a group setting. The treatment approach is modular and formulation-based (i.e., rather than delivering every module to all participants, modules will be selected based on patient need, as decided collaboratively between patient and treating team). These treatment components are provided within a case management framework that addresses practical issues such as educational and vocational rehabilitation, liaison with other services, accommodation needs, etc. This psychosocial intervention has been manualised based on the treatment model developed at the PACE Clinic in Melbourne and has also been used in several of our prior RCTs. The number of sessions delivered will be captured. Fidelity will be monitored by therapists rating their sessions on a therapy checklist and via independent rating of taped sessions. The standard will be to deliver CBCM sessions weekly, but frequency of sessions will be flexible depending on the participant’s needs. Sessions will ideally be weekly to fortnightly and of 30-50 minutes duration.

Medications

Step 3. Antidepressants (SSRIs) in Step 3: Oral capsules of fluoxetine 20mg or matched placebo daily for 6 months.

Participants will commence on 1 capsule of fluoxetine 20 mg or 1 capsule of the placebo pill, to be taken in the morning. The medication can be increased to fluoxetine 40 mg daily (or 2 placebo capsules) if there has been a poor clinical response after the first 6 weeks of Step 3.

At the end of Step 3 (Visit 9, Week 52), the antidepressant medication will be tapered as per standard clinical guidelines (164).

<u>Fast-fail Options</u>

At 12 weeks into Step 3 (Visit 8, Week 38), there will be three “fast-fail” options offered to the participant via a shared decision making process. Participants may continue the antidepressant or placebo with the addition of Omega-3 fatty acids or antipsychotic medication as follows (this decision will be made in consultation with the participant and their treating team):

1. Omega-3 fatty acid: 1.4-1.5g/day of marine fish oil containing eicosapentaoic/docosahexaenoic acid (EPA/DHA) in 4x 350mg or 6x 250mg capsules. Half of the capsules should be taken in the morning before breakfast and half in the evening before dinner.

2. Antipsychotic medication: **Quetiapine 50-400mg/day**. Extended-release quetiapine tablets will be prescribed as follows, taking into account treatment response and any possible side effects: -

	**Quetiapine Dosage**	**Timeframe allowed for next dose increase**
**Starting Dose**	50mg	Within 1 week from commencement
**Dose Schedule**	100mg	No earlier than 2 weeks from commencement of 100mg dose
	150mg	No earlier than 2 weeks from commencement of 150mg dose
	200mg	No earlier than 2 weeks from commencement of 200mg dose
**Target Dose**	300mg	No earlier than 4 weeks from commencement of 300mg dose
**Maximum Dose**	400mg	No increase allowed

3. Antipsychotic medication: **Aripiprazole 5-30mg/day**. Aripiprazole tablets will be prescribed as follows, taking into account treatment response and any possible side effects: -

	**Aripiprazole Dosage**	**Timeframe allowed for next dose increase**
**Starting Dose**	5mg	Within 1 week from commencement
**Dose Schedule**	10mg	No earlier than 2 weeks from commencement of

		10mg dose
	15mg	No earlier than 2 weeks from commencement of 15mg dose
**Target Dose**	20mg	No earlier than 4 weeks from commencement of 20mg dose
**Maximum Dose**	30mg	No increase allowed

8.2. Intervention Management

SPS and CBCM will be administered as per routine clinical practice at all sites. All sessions will be recorded in the site source documentation as per GCP and local regulatory requirements.

For medications, fluoxetine or matched placebo will be supplied as capsules in sealed HDPE bottles to the study site after receipt of required documents in accordance with all applicable regulatory requirements and Orygen procedures. Investigational product (IP) labels will be in accordance with all applicable regulatory procedures including an expiry date. Individual presentations will be labelled with the Investigator name, IP name, strength, dosage form, batch number, expiry date, storage conditions and protocol number.

Only participants who have reached randomisation into Step 3 will receive study drug, in accordance with all applicable regulatory requirements. Only authorised site staff as delegated by the PI may supply IP. Study drug supplies will be stored securely under the appropriate conditions in accordance with Australian Laws and clinical trial guidelines. The fluoxetine/ placebo capsules and the Omega-3 study medication must be stored below 25°C. The quetiapine and aripiprazole tablets must be stored below 30°C. All IP supplies must be stored in a secure area with access limited to authorised staff

Authorised and appropriately delegated study personnel will dispense the IP according to the randomisation schedule upon receipt of a valid prescription from the PI or PI- delegated medical doctor of the study team. Orygen Standard Operating Procedures (SOPs) and the study-specific Pharmacy Manual will be followed for the receipt, handling and accountability of the IP.

8.2.1. Accountability of medication supplies

Fluoxetine 20mg/placebo capsules, omega-3 fatty acid capsules, quetiapine tablets, and aripiprazole tablets are all IP in the STEP study. All IP supplied is for use only in this clinical study and should not be used for any other purpose.

The Principal Investigator is responsible for IP accountability, reconciliation and record maintenance. In accordance with all applicable regulatory requirements, the Investigator or delegated site staff must maintain IP accountability records throughout the course of the

study. These persons will document the amount of IP received from Orygen or the Sponsor contracted Clinical Research Organisation (or storage facility), the amount supplied and/or administered to and returned by participants.

An IP Dispensing Log must be kept current and will contain the following information:

- The identification of the participant to whom the drug was dispensed;
- The date(s) and quantity of the IP dispensed to the participant.

The inventory must be available for inspection by the Sponsor-designated unblinded study monitor during the study. IP supplies including participant returns will be collected at the end of the study by the study monitor, returned by the Investigator or designee to Orygen or authorised for destruction by Orygen. Where space at the Pharmacy for IP returns is limited, an unblinded monitor visit can be scheduled to verify and reconcile returns prior to issuing written destruction orders where applicable. When requested in writing by Orygen, unused drug supplies may be destroyed by the Investigator or delegate provided such disposition does not expose humans to risks from the drug. Records shall be maintained by the Investigator of any such alternate disposition of the IP. These records must show the identification and quantity of each unit disposed of, the method of destruction (taking into account the requirements of local law), and the person who disposed of the IP. Where IP is destroyed on-site, a record of destruction shall be issued. A copy of such records must be submitted to Orygen.

8.3. Concomitant Medication and Therapies

All psychiatric treatments administered to 24 months will be measured and recorded. If any concomitant medication is taken from the time of participant informed consent, the medication name, dose, frequency of dosing and reason for use will be recorded on the concomitant medication page in the source documentation. For adverse events or serious adverse events, where medication has been provided for management of the event, concomitant medication log entries along with Medical History Items will be documented as attributed to that AE or SAE number.

8.3.1. Permitted Medication and Therapies

Medications for medical conditions will be permitted throughout the trial. Brief use (lasting for less than one week) of sedative-hypnotics (benzodiazepine and benzodiazepine-like medications) will be permitted as clinically indicated (e.g., for insomnia or short-term anxiety). Where possible, reasons for this decision should be documented in the participant's source notes, the medical file or other specific essential documentation pertaining to study safety. During the follow-up period (12-24 months), which is

uncontrolled, participants can be prescribed medication as clinically indicated. All medications will be recorded.

8.3.2. Prohibited Medication and Therapies

In Steps 1 and 2 antidepressants will not be permitted. If a participant's depressive symptoms are so severe that it is felt by the treating team in consultation with the PI or their study team delegate that antidepressants cannot be safely withheld, the participant will be discontinued at this stage since the suicidal risk is almost certainly going to lead to exclusion anyway. The use of antipsychotics or mood stabilisers is not permitted.

8.3.3. Procedures for Monitoring Compliance with the Intervention

All psychological/behavioural and medicinal study treatments will only be used as directed in the protocol.

Fluoxetine/ placebo: During Step 3 participants will be requested to return all used and unused IP to the investigator or research assistants at their next visit, including any packaging where possible. Participants who have entered the Fast Fail option will also be asked to return all used and unused IP and bottles. The investigator or research assistant will count and record the number of tablets/capsules and bottles returned in the source notes. All participant returns are to be returned to the dispensing clinical trials pharmacy for accountability purposes. Records of overall dispensing and returns will be calculated and documented by the pharmacist involved in the study. Where possible, this data will be used to calculate compliance with medication for study analysis purposes.

Compliance with SSRI medication will also be monitored with a combination of regular text messages from the research assistant to the participant to gauge their compliance, and completing a medication compliance checklist every fortnight.

SPS and CBCM: Sessions will be audiotaped and rated by blinded raters who do not have direct contact with participants. This is to ensure fidelity to these psychological interventions and prevent accidental unblinding of assessments for Step 2 (single-blind stage of study). In addition, fidelity will be monitored by therapists rating their own sessions on an established checklist of therapeutic interventions. Any additional psychosocial interventions delivered will also be documented.

8.4. Method of Assigning Participants to Treatment Groups and Randomisation

Participant eligibility will be established before randomisation at Step 2 and 3. The randomisation schedule will be generated by a statistician independent of the study team. Computer generated random numbers will be used to determine the randomisation schedules for Step 2 and 3. A participant identification (ID) number will be allocated to each

participant who provides informed consent, so that participants can be identified without making assumptions about their subsequent eligibility for the study. If a participant fails screening and is not randomised, or discontinues from the trial, the participant ID number will not be reused.

8.5. Blinding

In Step 2, the research assistant will be blind to treatment allocation (single-blind). In the double-blind Step 3 (fluoxetine/placebo), the participant, treating clinicians, and research personnel (including research assistants, investigators, the study statistician, and project manager) will all remain blinded to IP treatment allocation. An unblinded monitor will review the Pharmacy files in accordance with Sponsor responsibilities.

The IP blind will be maintained by ensuring that the packaging, appearance, colour and taste of fluoxetine and matched placebo capsules are identical. A randomisation number will be allocated at the commencement of Step 2 and a second randomisation number will be allocated at the commencement of Step 3. The treating doctors will not be allowed access to the randomisation schedule until the end of the intervention periods of Step 2 and 3 (other than in case of management of safety emergencies). While participants and treating doctors will be unblinded at the conclusion of the treatment period (end of Step 3, see below), other research personnel who will continue the research assessments will remain blinded throughout the follow-up period where possible.

<u>Treatment allocation concealment</u>

The process of allocation will occur via a predetermined randomisation schedule, and research personnel will have no way of predicting which group the participant will be allocated to.

<u>Methods for unblinding</u>

Individual treatment codes that indicate the treatment randomisation for each participant will be made available to the coordinating principal investigator and pharmacists in case of emergency. The treatment code will not be broken throughout the treatment period except in medical emergencies when the appropriate management of the participant necessitates knowledge of the treatment randomisation.

Where necessary, unblinding of the participant's treatment allocation will be done through accessing the on-line electronic Case Report Form (eCRF). Investigators and the project managers will be allocated unique logins which allow them to access the treatment code when necessary. A series of questions will need to be answered before the treatment allocation is revealed.

Code Break Procedures

There will be 24-hour, 7 days a week emergency access to the treatment allocations for the PI or qualified delegate in the case the randomisation code for an individual participant needs to be broken.

Prior to breaking the code, the PI or qualified delegate should endeavour to contact the Sponsor's monitor at Orygen and study Project Manager where possible. All details of the code-break must be documented as outlined in the study-specific Pharmacy Manual. If the blind has been broken, the Investigator must document the date and the reason the blind was broken in the participants' source documents and eCRF. They will also complete an unblinding log in order to track code breaks accordingly. In instances of unblinding, the number of persons who become aware of the unblinded information should be kept to a minimum.

Unblinding can take place under three circumstances:

1. **At the end of the intervention period (Step 3) or after early discontinuation**:

If the participant wishes to continue on the SSRI/ placebo, the participant and treating doctors can be unblinded in order for appropriate clinical decisions to be made about the participants' ongoing clinical management. It is important that the treating doctor is aware of whether the participant has been on placebo or fluoxetine before further medication is prescribed. Where practicable, it is important that the Research assistants complete the Month 12 follow-up assessment prior to this unblinding. Any decisions about unblinding will be made by the treating doctor in consultation with the PI or delegate and fully documented in the participant's source notes.

Research assistants, who will undertake all outcome assessments, will remain blind throughout the trial, and where possible, during the follow-up period. Treating doctors will be reminded not to discuss the treatment allocation in front of research assistants during the follow-up period.

2. **In case of an emergency**:

Unblinding under emergency circumstances must only be done after consultation with the PI or a suitable medically-qualified delegate. An authorisation from the PI to unblind a participant must always be documented in the participant's medical file and once unblinded, all corresponding pages on the eCRF for that participant will be clearly marked with details relating to the unblinding.

3. **As per DSMC decision.**

There may be instances where following a Data Safety Monitoring Committee meeting, a decision may be made to unblind certain participants, place the study on hold or terminate

the study. Where a decision to unblind has been majority voted, the PI will unblind in accordance with the DSMCs voting decision.

8.5.1. Distribution of Wallet Cards for Step 3 (medication stage)

As participants enter Step 3 of the study, they will be given a wallet card to keep as an important reference point for study contacts.

The reason for introducing it here is twofold:

The wallet card will refer to the participant being on medication (either placebo or fluoxetine,) and this doesn't happen until they are randomised at Step 3.

Issuing the wallet card will provide an opportunity to educate the participant about what needs to be done in an emergency situation. At this point, we should aim to encourage the participants to let the treating doctor know they are in a clinical trial if they happen to present in an emergency situation. We will also suggest that the participant takes a photo of their wallet card so they have this on their phone and also encourage them to put the study team numbers into their phone.

The wallet card will contain the following information:

Participant name;

Screening ID;

Information stating the name of the trial, the centre it's being conducted at and the fact that the participant is on medication;

Names and numbers of people who need to be contacted in an emergency.

9. Safety Measures

The definitions of AEs and SAEs are given below.

9.1. Definitions:

Adverse Event (AE):

> Any untoward medical occurrence in a participant administered an intervention that does not necessarily have a causal relationship with this treatment. An AE can therefore be any unfavourable and unintended sign (including an abnormal laboratory finding), symptom, or disease temporally associated with the use of an intervention, whether or not related to the intervention.

9.2. Serious Adverse Event (SAE)

An SAE is any untoward medical occurrence that at any administration level:

1. Results in death;
2. Is life-threatening;

   Life-threatening in the definition of serious refers to an event in which the participant was at risk of death at the time of the event, it does not refer to an event which hypothetically might have caused death if it were more severe.

3. Requires inpatient hospitalization or prolongation of existing hospitalization;
4. Results in persistent or significant disability/incapacity;
5. Is a congenital anomaly/birth defect;
6. Is an important medical event that although not immediately life-threatening or result in death or hospitalisation, based upon appropriate medical and scientific judgment, may jeopardize the participant and/or require intervention to prevent one of the above- listed outcomes.

Outpatient treatment in an emergency room is not in itself an SAE, although the reasons for it may be (e.g., bronchospasm, laryngeal oedema).

Hospital admissions and/or surgical operations planned before or during a study are not considered AEs if the illness or disease existed before the participant was enrolled in the study, provided that it did not deteriorate in an unexpected way during the study.

Pregnancy is not in itself considered an SAE however, should a pregnancy be reported on study, the participant will be asked to provide some information on the Sponsor’s pregnancy reporting form such that the outcome of the pregnancy can be monitored (criterion 5 above). If an AE/SAE arises as a result of the pregnancy, this will be recorded on the Sponsor AE/SAE form in the standard manner.

Mental health illness worsening or progression should be recorded as an adverse event (e.g. exacerbation of illness, increase in severity of depressive symptoms). While this is an expected event, an AE form will be completed. If the disease progression satisfies one of the SAE criteria described above, it will be recorded as an SAE in the standard manner (e.g., transition that results in hospitalisation is an SAE).

9.3. Unexpected Adverse Reactions

Adverse reactions are all noxious and unintended responses to an intervention related at any dose or frequency. A reaction with a nature, trending or severity that is not consistent with the available product information, Investigator’s Brochure or prior use history.

The phrase “responses to an intervention” means that a causal relationship between the intervention and an adverse event is at least a reasonable possibility, i.e. the relationship cannot be ruled out.

9.4. AE and SAE Assessment

9.4.1. Causality

The causality of AEs/SAEs (i.e., their relationship to intervention or treatment) will be assessed by a study doctor at the study site. Assessing causality requires considering whether there was a reasonable possibility that the event may have been caused by the intervention. For medical AEs, a medically-qualified PI delegate will conduct the causality assessment.

Ambiguous cases will be considered as having a possibility of a causal relationship unless further evidence becomes available to refute this.

9.4.2. Severity

The Investigator or suitably qualified delegate will make an assessment of intensity for each AE and SAE reported during the study. The assessment will be based on the Investigator’s clinical judgement. The intensity of each AE/SAE recorded should be assigned to one of the following categories:

Mild: An event that is easily tolerated by the participant, causing minimal discomfort and not interfering with everyday activities or is considered mild by diagnosis.

Moderate: An event that is sufficiently discomforting to interfere with normal everyday activities or is considered moderate by diagnosis.

Severe: An event that prevents normal everyday activities or is considered severe by diagnosis.

An AE that is assessed as severe should not be confused with an SAE. Severity is a category utilised for rating the intensity of an event, and both AEs and SAEs can be assessed as severe. An event is defined as ‘serious’ when it meets one of the pre-defined outcomes as described in Section 9.2.

9.5. Recording of Adverse Events and Serious Adverse Events

Adverse event and SAE data may be derived from a number of sources such as clinical results/reports, medical file reviews, and spontaneous reporting via non-leading questioning of the participant. Adverse events might be generated by asking the participant an open-ended question such has “How have you been feeling since your last visit?” Using such open-ended questions will avoid leading the participant and exacerbating the adverse event data collected. Adverse events that have been initially reported and re-reported will be

determined by the PI or suitably-qualified delegate as continuing or the initial AE resolved. Adverse events that increase in severity will be recorded as new AEs.

Adverse event recording will be concise using standard, acceptable medical terms. Whenever practical, the events (AEs or SAEs) recorded should not be a procedure or a clinical measurement, (e.g. vital signs) but should reflect the diagnosis based on the abnormal measurement.

Adverse events occurring after screening but prior to baseline will be recorded as medical history, unless possibly related to a study procedure, in which case they will be reported as an AE. AEs will be captured and reported until the AE resolves, stabilises or the participant is lost to follow-up.

The AE description, start and stop dates, intensity, causality and outcome will be recorded, as well as any actions taken to manage the AE including the administration of concomitant medication.

Adverse events and SAEs are to be recorded on the Sponsor approved form or other suitable site documentation provided the minimum regulatory reporting items are met.

9.6. Expedited and Prompt Reporting of SAEs

Any AE that is serious (Section 9.2) and occurs during the course of the study, irrespective of the treatment received by the participant, must be reported to Orygen within 24 hours of the Investigator or designee becoming aware of the SAE regardless of the relationship to the IP. As much information as possible, as is available at the time of recording, is to be provided on the Sponsor SAE form or site equivalent. The Investigator should always provide an assessment of causality at the time of the initial report. A follow-up report will be completed when outstanding information becomes available, when there is a significant change in the event or when the event resolves.

Orygen is responsible for reporting any Suspected Unexpected Serious Adverse Reactions (SUSAR) to the regulatory authorities in accordance with regulatory requirements however may seek assistance from the site for the collection of data and reporting. SAEs and SUSARs will be reported by the Investigator’s team to the governing Human Research Ethics Committee (HREC) in accordance with HREC requirements.

9.7. Handling in Cases of Unresolved AEs and SAEs at Completion or Withdrawal

All IP-related AEs and all SAEs must be followed up until resolution unless, in the Investigator’s opinion, the condition is unlikely to resolve. All other AEs and SAEs must be followed up until resolution, stabilisation or until the participant has been appropriately referred for follow-up by another medical care provider.

9.8. Pregnancy

If a participant falls pregnant during Step 3, she will discontinue from medicinal treatment. Pregnancy in itself is not regarded as an SAE. However, the outcome of all pregnancies (spontaneous miscarriage, elective termination, normal birth or congenital abnormality) will be followed up. All reports of congenital abnormalities and other birth defects will be recorded as SAEs. Spontaneous miscarriages will be reported as SAEs, though uncomplicated elective abortions will not.

9.9. Procedures in Case of Medical Emergency

The coordinating/Principal Investigator (or delegate) is responsible for ensuring that procedures and expertise are available to handle medical emergencies during the study. A medical emergency usually constitutes an SAE and will be reported in the standard manner.

9.10. Procedures in Case of Overdose

An intentional overdose of medication with either intent to cause harm or suicide will be recorded as an AE unless it satisfies one of the SAE criteria listed in Section 10.1.2. For the purpose of the study the nature of the intent will be determined by the treating doctor, or the coordinating/Principal Investigator or their delegate. The treating doctor or coordinating/Principal Investigator will carefully record their reasons for determining whether or not an event is to be classified as an intentional overdose.

9.11. Managing participant distress in the EXPECT sub study

If parents are distressed by the interview, additional support will be offered by Orygen psychiatrists. Interviews will only be at times when one of the clinically trained investigators on the study are available. Young people’s information will not be known to the researchers and hence researchers will not provide prognostic information or confirm/disconfirm parents’ beliefs about their young person. This information will be made clear during the consent process. If the parents remain distressed, they will be referred to their local general practitioner (GP). They will need to have an identified GP to participate in the study, as specified in the PICF.

10. Study Oversight

10.1. Data Safety and Monitoring Committee

A DSMC will be established for review of all available safety data at various points throughout the study as outlined in the study-specific DSMC Charter. The main purpose of the DSMC will be to protect the safety and interests of the participants included in the study. Stopping rules based on safety are discussed in Section 7.8.5. These will be used as

guidelines only for individual participants and will not be the only basis on which to recommend stopping the trial. Minutes will be kept of all DSMC meetings.

11. Ethical Considerations

11.1. Review by an Ethics Committee

Prior to the commencement of the study, the protocol and any amendment(s), Participant Information and Consent Form, other information provided to participants (including advertising) and product information will be submitted to the appropriate Institutional Human Research Ethics Committee (HREC).

The approval/favourable opinion should refer to the study by title, protocol number and version and dates of documentation reviewed and approved. A copy of the signed and dated letter of approval (on institutional letterhead) will be provided to the site and Orygen prior to study commencement. A list of the voting HREC members and their institutional affiliations may be requested before study initiation.

During the course of the study, the Principal Investigator (or delegate) is required to submit to the HREC the following: amendments to the protocol, suspected unexpected serious adverse events (SUSARs) as per the committee's requirements, site-specific updates as agreed to by the Investigator and respective HREC, and any additional information as required (e.g., SUSARs reported by other investigative sites, amendments to the Investigator Brochure/product information and administrative changes to the protocol). The PI must submit progress reports to the HREC according to local regulations and guidelines.

Protocol amendments that may impact on participant safety or the validity of the study will be agreed upon by the PI and Sponsor and submitted to the reviewing HREC for approval prior to implementation.

At conclusion of the study, the Investigator is required to inform the HREC in writing that the study has ended and no further activities regarding this protocol will be conducted at the site.

11.2. Ethical Conduct of the Trial

The Investigator will ensure that the study is performed in accordance with ethical principles that have their origin in the Declaration of Helsinki and are consistent with Good Clinical Practice (GCP) as adopted in Australia, The NHMRC National Statement on Ethical Conduct in Human Research and the NHMRC Australian Code for the Conduct of Research.

11.3. Investigator Responsibilities

The study cannot commence until the PI has received favourable opinion/approval from the reviewing HREC as described in Section 11.1. Prior to study start, the investigator is required to sign a protocol signature page confirming his/her agreement to conduct the study in accordance with these documents and all of the instructions and procedures found in this protocol and to give access to all relevant data and records to Orygen monitors and auditors, HREC and regulatory authorities as required. If an inspection of the clinical site is requested by any regulator, the investigator must inform Orygen immediately that this request has been received.

11.4. Financing and Insurance

The Sponsor, Orygen will have full and current clinical trials insurance under the governance of the Victorian Managed Insurance Authority. Appropriate indemnification of the study site(s) will be in accordance with institutional HREC requirements.

This study is financed by an NIH Cooperative Agreement.

11.5. Participant Information and Consent

Prior to participation in the research study, each participant will undergo a complete consenting interview with the delegated study team members and provide written (signed and personally dated) consent on the relevant HREC approved form. The contents and process of obtaining informed consent will be in accordance with all applicable regulatory requirements including those required for the consenting of vulnerable participants and those under the age of 18.

All eligible participants will have the study explained by the PI, Co-Investigator or suitably-qualified delegate. They will receive a full explanation, in lay terms of the aims of the study, the discomfort, risks and benefits in taking part for the duration of the study as well as procedures for compensation in case of injury during the research project. It will be explained that the study is for research purposes and may provide no clear benefit to the individual, and it will be pointed out that they are free to discontinue or withdraw from the study at any time without prejudice or affecting their routine clinical care. Each participant will acknowledge receipt of this information by giving written informed consent for participation in the study. The participant will be given a full copy (all pages) of the PICF including signatory page to retain.

11.6. Participant Data Protection

The PICF will explain that study data will be safely stored in computer databases as well as in paper form. The maintenance of confidentiality will be in accordance with national data and

privacy legislation. Participants in this database will be identified by a unique participant identification number and their initials. The PICF will also explain that for data verification purposes, authorized representatives of Orygen, regulatory authorities, HRECs or sites may require direct access to parts of the hospital or practice records relevant to the study, including medical history.

12. Study Management

12.1. Monitoring and Auditing

Study monitoring will be performed in accordance with applicable regulations, ICH-GCP, and Orygen Standard Operating Procedures (SOPs).

Before the start of the study, an Orygen monitor will contact a representative of the study team to conduct a site feasibility assessment to ensure facilities are adequate and discuss responsibilities with the site staff with regards to following the protocol and regulatory and ethical requirements. During the course of the study, the Orygen monitor will regularly contact and visit the site to monitor study progress, confirm protocol, regulatory and ethical adherence, confirm data accuracy and provide information and site support. The PI agrees to allow the monitor direct access to all relevant documents and to allocate their time and the time of their staff to the monitor to discuss findings and any relevant issues.

Site staff will be provided with monitor and project manager contact details in the event they have queries or require assistance.

12.2. Audits and Inspections

An audit is a systematic and independent examination of study-related activities and documents to determine whether the approved study-related activities were conducted, and the data were recorded, analysed and accurately reported according to the protocol, SOPs, GCP and the applicable regulatory requirement(s).

Authorised representatives of Orygen, a regulatory authority, or the HREC/Research Governance Office may visit the site to perform audits or inspections. The Investigator should contact Orygen immediately if they are contacted by a regulator about an inspection at their site. If an audit or inspection occurs, the PI and institution agree to allow the auditor/inspector direct access to all relevant documents and allocate their time and the time of their staff to the auditor/inspector to discuss findings and any relevant issues.

12.3. Training of Staff

As per GCP, each individual involved in the conduct of a study should be qualified by education, training and experience to perform his or her respective task(s).

The PI will maintain a record of all individuals involved in the study. The PI will ensure that appropriate training relevant to the study is given to staff, and that they will receive any new information of relevance to the performance of this study.

Site Initiation Visit training will be provided by Orygen's monitor and all training records retained for audit and tracking purposes.

12.4. Changes to the Protocol

Study procedures will not be changed without the mutual agreement of the PI and Orygen.

If it is necessary for the study protocol to be amended, the amendment or a new version of the study protocol must be submitted and approved by the relevant HREC before implementation unless the safety of participants is at risk. Local requirements must be followed.

If a protocol amendment requires a change to the PICF, approval of the revised PICF by Orygen and by the HREC is required before the revised form can be used to consent new potential participants. For existing participants, they will be asked to consent to the revised and approved PICF prior to taking part in changes to study conduct.

12.5. Protocol Compliance

The study shall be conducted as described in the approved protocol. Any significant deviation must be documented in the source documentation and in the eCRF where applicable. If a deviation or change to a protocol is implemented to eliminate an immediate hazard(s) prior to obtaining HREC approval/favourable opinion, as soon as possible the deviation or change will be submitted to the HREC for review and approval.

The PI must comply with all the terms, conditions and obligations of the Clinical Trial Research Agreement. In the event of any inconsistency between this protocol and the study agreement, the study agreement shall prevail.

12.6. Study Timetable and Termination

The planned start date for this study in Quarter 1 of 2016. The proposed completion date is in Quarter 1 2020. Orygen reserves the right to terminate the study at any stage for any reason including funding considerations.

12.7. Data handling and record retention

All study essential documentation in accordance with GCP and Australian regulatory requirements will be retained for a minimum period of 15 years (following completion of the study) or in accordance with the approving HRECs policy (whichever is the longest).

Should the Investigator wish to assign the study data and/or documentation to another party or move to another location, Orygen must be notified. In addition, the Investigator

should notify Orygen prior to destruction of any study documentation, regardless of the timeframe lapsed.

The Sponsor should notify the Investigator/Institution in writing when study-related records are no longer required to be kept.

13. Data Management

13.1. Documentation

A screening log of all potential participants will be maintained. This will include potential participants who were considered but later deemed ineligible due to meeting one of the exclusion criteria or due to investigator discretion. The reasons for exclusion will also be recorded against their ineligibility status. All participants who are randomised will receive a randomisation number.

Source data will be constituted as documents where the trial data are first recorded. This will include the hard-copy questionnaires and measures, and raw data such as tapes of CBT sessions, laboratory results (if applicable), and hospital records. These data will be retained in a secure location at each centre with access only to delegated study team members. Electronic case report forms (eCRF) will also be used for documentation and reporting data to Orygen. For the measures in which the results are entered directly into the eCRF, the eCRF becomes the source documentation (e.g. DACOBS, CTQ).

Research assistants or other suitably qualified delegate will be responsible for entering data into the eCRF. All protocol-related and other relevant data will be documented in the participant’s medical file/history. This includes information that the participant participated in the study, date of study entry and completion or discontinuation, visit dates, administration of study medication and serious adverse events.

13.2. Database management

Internet-based database applications will be used at Orygen, with relevant data being entered online. The database applications will be modelled on previous studies that have adopted the eCRF data collection method. Data collected in the eCRF will be entered via a secure website. Access to the eCRF will be restricted to study personnel and the level of access will be set to maintain the privacy and confidentiality of participant information. The eCRF will be managed by staff at Orygen, who will also be responsible for data checking and verification. After monitoring and data management queries are resolved and complete, the clinical trial database will be locked and the eCRF signed off by the PI for each participant. All data will be exported into the appropriate software to enable statistical analysis on secure Orygen servers. All study team members will need to apply for access to the eCRF through the STEP Project Manager or coordinating investigator. These processes will be recorded and

kept with the study essential documentation.

13.3. Quality Control and assurance

As the trial sponsor, Orygen will oversee and govern all regulatory aspects of the study across all sites. Monitoring of source data and the eCRF across sites will be performed by a designated monitor on a regular basis. Any inconsistencies, errors, protocol deviations and protocol violations will be identified by the monitor and reported back to the applicable site for prompt resolution.

Precise records will be kept in order to form an audit trail of all study-related activities and an audit trail of all changes made to data in the eCRF will also be maintained in accordance with routine Quality Assurance practices. In addition to onsite monitoring, review of the study data will be performed offsite on an ongoing basis throughout the study. Any monitoring or data management queries that arise from these reviews will be sent to the site in on-line Monitoring or Data Clarification Queries. An unblinded monitor will also attend any location where IP is stored and dispensed. Monitors will review site records to ensure compliance with the study protocol and GCP. All IP destructions must be authorised by the Sponsor Orygen prior to being sent for destruction or returned to Orygen.

Data quality will be ensured by performing data entry checks for consistency between raw data, the eCRF, and the data entry database. These checks will be performed during data entry so that discrepancies can be resolved immediately. This will be performed remotely through electronic means and in-person at each site.

Each site will maintain a record of all personnel involved in the study as delegated by the PI. In consultation with the central coordinating centre, Orygen, each trial centre will ensure that appropriate training is provided to study personnel, and that any new information of relevance to the performance of this study is forwarded to the staff involved. All training will be documented and form part of the study essential documents.

14. Statistical Methods

14.1. Methodology

The primary analysis is to compare the two Step 2 treatments on the 6-month Global Functioning: Social and Role Scale score (130) using analysis of covariance with the baseline Global Functioning: Social and Role Scale score as the covariate. The treatment options in Steps 2 and 3 correspond to 4 treatment regimes for the non-responders. Comparing fluoxetine and placebo from Step 3 is equivalent to collapsing these regimes into two groups. A secondary analysis will be to compare these two groups using ANCOVA in terms of 12-month Global Functioning: Social and Role Scale score. Comparing the different treatment regimes will also be considered. Survival analysis will also be used for comparison

of transition rates. Other secondary outcome measures at the various time points will be examined using appropriate techniques such as ANCOVA, chi-square tests and multi-level modelling. Potential confounders and predictors will also be taken into account. If the amount of missing values is non-trivial, then imputation techniques such as multiple imputation will be considered.

Data analysis in the EXPECT sub study: Participants' demographic characteristics and beliefs about the degree of risk will be described quantitatively. Their beliefs regarding children's autonomy in regards to being identified as at-risk, as well as regarding interventions will be examined using thematic analysis. Their responses with regards to their experience of their son/daughter being identified as being at-risk will be examined using interpretative phenomenological analysis.

14.2. Analysis Populations

14.2.1. Efficacy

Intention to Treat (ITT) analysis will be conducted.

14.2.2. Safety Analysis

The safety population will consist of all participants who were randomised and received at least one SPS session in Step 1 and had at least one post-baseline assessment.

Statistical testing will be performed on safety data as described in the Statistical Analysis Plan (SAP).

Adverse events will be coded using MedDRA where possible and summarised by system organ classes (SOCs) and preferred term (PT).

A summary of the number and percentage of participants with the following AEs will be displayed by treatment groups:

- All AEs
- Drug-related AEs
- Serious AEs
- AEs leading to permanent discontinuation of study drug

14.3. Interim Analysis

No interim analyses are planned.

15. Publication and Use of Study Findings

After approval by the coordinating/Principal Investigator, sub-investigators and statisticians, results of the study will be published in peer-reviewed scientific journals and presented at scientific conferences. The final results will be published after termination of the study. Where participants have asked to see the results of the study, these results will be provided to them in due course. The order of authors will be at the discretion of the coordinating/principal investigator. Factors that the coordinating/Principal Investigator may take into consideration are the following: participation in organising the study, participation in meetings and the ongoing development of the study, manuscript production and general involvement in the study. More details pertaining to the publication policy can be found in the Clinical Trial Agreement.

References

1. Zeidler J, Slawik L, Fleischmann J, Greiner W. The costs of schizophrenia and predictors of hospitalisation from the statutory health insurance perspective. Health economics review. 2012;2(1):9.
2. Insel TR. Rethinking schizophrenia. Nature. 2010;468(7321):187-93.
3. McGorry PD, Killackey E, Yung A. Early intervention in psychosis: concepts, evidence and future directions. World Psychiatry. 2008;7(3):148-56.
4. McGorry PD, Nelson B, Goldstone S, Yung AR. Clinical staging: A gateway to better health and social outcomes for psychotic and mood disorders. Canadian Journal of Psychiatry. 2010;55(8):486-97.
5. McGorry PD, Edwards J, Mihalopoulos C, Harrigan S, Jackson H. EPPIC - An evolving system of early detection and optimal management. Schizophrenia Bulletin. 1996;22:305-26.
6. Loebel AD, Lieberman JA, Alvir JM, Mayerhoff DI, Geisler SH, Szymanski SR. Duration of psychosis and outcome in first-episode schizophrenia. Am J Psychiatry. 1992;149(9):1183-8.
7. Norman RM, Malla AK. Duration of untreated psychosis: a critical examination of the concept and its importance. Psychol Med. 2001;31(3):381-400.
8. Large M, Babidge N, Andrews D, Storey P, Nielssen O. Major self-mutilation in the first episode of psychosis. Schizophr Bull. 2009;35(5):1012-21.
9. Nielssen O, Large M. Rates of homicide during the first episode of psychosis and after treatment: a systematic review and meta-analysis. Schizophr Bull. 2010;36(4):702-12.
10. Wyatt RJ, Henter I. Rationale for the study of early intervention. Schizophr Res. 2001;51(1):69-76.
11. McGorry PD, & Singh, B.S. Schizophrenia: risk and possibility. In: Raphael B, & Burrows, G.D., editor. Handbook of Preventive Psychiatry. Amsterdam, The Netherlands: Elsevier; 1995. p. 492-514.
12. Yung AR, McGorry PD. The prodromal phase of first-episode psychosis: Past and current conceptualizations. Schizophrenia Bulletin. 1996;22(2):353-70.
13. Köhn D, Niedersteberg A, Wieneke A, Bechdolf A, Pukrop R, Ruhrmann S, et al. Frühverlauf schizophrener Ersterkrankungen mit langer Dauer der unbehandelten Erkrankung – eine vergleichende Studie. Fortschritte der Neurologie, Psychiatrie. 2004;72:88-92.

14. Häfner H, an der Heiden W. The course of schizophrenia in the light of modern follow-up studies: the ABC and WHO studies. Eur Arch Psychiatry Clin Neurosci. 1999;249 Suppl 4:14-26.

15. Haefner J, Loeffler W, Maurer K, Hambrecht M, an der Heiden W. Depression, negative symptoms, social stagnation and social decline in the early course of schizophrenia. Acta Psychiatrica Scandinavica. 1999;100(2):105-18.

16. Huber G, Gross G, Schuttler R, Linz M. Longitudinal studies of schizophrenic patients. Schizophrenia Bulletin. 1980;6:592-605.

17. Yung AR, McGorry PD. The initial prodrome in psychosis: Descriptive and qualitative aspects. Australian and New Zealand Journal of Psychiatry. 1996;30(5):587-99.

18. Sullivan HS. The onset of schizophrenia. American Journal of Psychiatry. 1927;6:105-34.

19. Meares A. The diagnosis of prepsychotic schizophrenia. Lancet. 1959; i:55-9.

20. Häfner H. Prodrome, onset and early course of schizophrenia. Murray, Robin M (Ed); Jones, Peter B (Ed); et al (2003) The epidemiology of schizophrenia. New York, NY, US: Cambridge University Press; 2003. p. 124-47.

21. Agerbo E, Byrne M, Eaton WW, Mortensen PB. Schizophrenia, marital status, and employment: A forty-year study. Schizophrenia Research. 2003;60(1, Supplement 1):32.

22. Yung AR, McGorry PD, McFarlane CA, Jackson HJ, Patton GC, Rakkar A. Monitoring and care of young people at incipient risk of psychosis. Schizophr Bull. 1996;22(2):283-303.

23. Cornblatt B, Lencz T, Obuchowski M. The schizophrenia prodrome: treatment and high-risk perspectives. Schizophr Res. 2002;54(1-2):177-86.

24. Fusar-Poli P, Borgwardt S, Bechdolf A, Addington J, Riecher-Rossler A, Schultze-Lutter F, et al. The psychosis high-risk state: a comprehensive state-of-the-art review. JAMA Psychiatry. 2013;70(1):107-20.

25. McGorry PD, Nelson B, Amminger GP, Bechdolf A, Francey SM, Berger G, et al. Intervention in individuals at ultra-high risk for psychosis: a review and future directions. J Clin Psychiatry. 2009;70(9):1206-12.

26. Fusar-Poli P, Bonoldi I, Yung AR, Borgwardt S, Kempton MJ, Valmaggia L, et al. Predicting Psychosis: Meta-analysis of Transition Outcomes in Individuals at High Clinical Risk. Archives of general psychiatry. 2012;69(3):220-9.

27. Nelson B, Yuen HP, Wood SJ, Lin A, Spiliotacopoulos D, Bruxner A, et al. Long-term Follow-up of a Group at Ultra High Risk ("Prodromal") for Psychosis: The PACE 400 Study. JAMA Psychiatry. 2013;70(8):793-802.

28. Addington J, Cadenhead KS, Cannon TD, Cornblatt B, McGlashan TH, Perkins DO, et al. North American Prodrome Longitudinal Study: a collaborative multisite approach to prodromal schizophrenia research. Schizophr Bull. 2007;33(3):665-72.

29. Seidman LJ, Giuliano AJ, Meyer EC, Addington J, Cadenhead KS, Cannon TD, et al. Neuropsychology of the prodrome to psychosis in the NAPLS consortium: relationship to family history and conversion to psychosis. Archives of general psychiatry. 2010;67(6):578-88.

30. Cannon TD, Cadenhead K, Cornblatt B, Woods SW, Addington J, Walker E, et al. Prediction of psychosis in youth at high clinical risk: a multisite longitudinal study in North America. Arch Gen Psychiatry. 2008;65(1):28-37.

31. McGorry PD, Yung AR, Phillips LJ, Yuen HP, Francey S, Cosgrave EM, et al. Randomized controlled trial of interventions designed to reduce the risk of progression to first-episode psychosis in a clinical sample with subthreshold symptoms. Archives of General Psychiatry. 2002;59(10):921-8.

32. van der Gaag M, Smit F, Bechdolf A, French P, Linszen DH, Yung AR, et al. Preventing a first episode of psychosis: meta-analysis of randomized controlled prevention trials of 12 month and longer-term follow-ups. Schizophr Res. 2013;149(1-3):56-62.

33. Hutton P, Taylor PJ. Cognitive behavioural therapy for psychosis prevention: a systematic review and meta-analysis. Psychol Med. 2013:1-20.

34. Bechdolf A, Wagner M, Ruhrmann S, Harrigan S, Putzfeld V, Pukrop R, et al. Preventing progression to first-episode psychosis in early initial prodromal states. Br J Psychiatry. 2012;200(1):22-9.

35. Nordentoft M, Thorup A, Petersen L, Ohlenschlaeger J, Melau M, Christensen TO, et al. Transition rates from schizotypal disorder to psychotic disorder for first-contact patients included in the OPUS trial. A randomized clinical trial of integrated treatment and standard treatment. Schizophr Res. 2006;83(1):29-40.

36. McGlashan TH, Zipursky RB, Perkins D, Addington J, Miller T, Woods SW, et al. Randomized, double-blind trial of olanzapine versus placebo in patients prodromally symptomatic for psychosis.[see comment]. American Journal of Psychiatry. 2006;163(5):790-9.

37. Amminger GP, Schafer MR, Papageorgiou K, Klier CM, Cotton SM, Harrigan SM, et al. Long-chain omega-3 fatty acids for indicated prevention of psychotic disorders: a randomized, placebo-controlled trial. Arch Gen Psychiatry. 2010;67(2):146-54.

38. Cornblatt BA, Lencz T, Smith CW, Olsen R, Auther AM, Nakayama E, et al. Can antidepressants be used to treat the schizophrenia prodrome? Results of a prospective, naturalistic treatment study of adolescents. J Clin Psychiatry. 2007;68(4):546-57.

39. Fusar-Poli P, Valmaggia L, McGuire P. Can antidepressants prevent psychosis? Lancet. 2007;370(9601):1746-8.

40. Fusar-Poli P, Frascarelli M, Valmaggia L, Byrne M, Stahl D, Rocchetti M, et al. Antidepressant, antipsychotic and psychological interventions in subjects at high clinical risk for psychosis: OASIS 6-year naturalistic study. Psychol Med. 2015;45(6):1327-39.

41. Cantor-Graae E. The contribution of social factors to the development of schizophrenia: a review of recent findings. Can J Psychiatry. 2007;52(5):277-86.

42. Berton O, McClung CA, Dileone RJ, Krishnan V, Renthal W, Russo SJ, et al. Essential role of BDNF in the mesolimbic dopamine pathway in social defeat stress. Science. 2006;311(5762):864-8.

43. Yung AR, Buckby JA, Cosgrave EM, Killackey EJ, Baker K, Cotton SM, et al. Association between psychotic experiences and depression in a clinical sample over 6 months. Schizophrenia Research. 2007;91(1-3):246-53.

44. Garner B, Pariante CM, Wood SJ, Velakoulis D, Phillips L, Soulsby B, et al. Pituitary Volume Predicts Future Transition to Psychosis in Individuals at Ultra-High Risk of Developing Psychosis. Biological Psychiatry. 2005;58(5):417-23.

45. Cornblatt BA, Lencz T, Smith CW, Correll CU, Auther AM, Nakayama E. The schizophrenia prodrome revisited: a neurodevelopmental perspective. Schizophr Bull. 2003;29(4):633-51.

46. Chen G, Hasanat KA, Bebchuk JM, Moore GJ, Glitz D, Manji HK. Regulation of signal transduction pathways and gene expression by mood stabilizers and antidepressants. Psychosomatic Medicine. 1999;61(5):599-617.

47. Michael-Titus AT, Bains S, Jeetle J, Whelpton R. Imipramine and phenelzine decrease glutamate overflow in the prefrontal cortex--a possible mechanism of neuroprotection in major depression? Neuroscience. 2000;100(4):681-4.

48. Sanchez V, Camarero J, Esteban B, Peter MJ, Green AR, Colado MI. The mechanisms involved in the long-lasting neuroprotective effect of fluoxetine against MDMA ('ecstasy')-induced degeneration of 5-HT nerve endings in rat brain. British journal of pharmacology. 2001;134(1):46-57.

49. Phillips LJ, McGorry PD, Yuen HP, Ward J, Donovan K, Kelly D, et al. Medium term follow-up of a randomized controlled trial of interventions for young people at ultra high risk of psychosis. Schizophr Res. 2007;96(1-3):25-33.

50. Morrison AP, French P, Parker S, Roberts M, Stevens H, Bentall RP, et al. Three-Year Follow-up of a Randomized Controlled Trial of Cognitive Therapy for the Prevention of Psychosis in People at Ultrahigh Risk. Schizophr Bull. 2007;33(3):682-7.

51. Norman RM, Windell D, Manchanda R, Harricharan R, Northcott S. Social support and functional outcomes in an early intervention program. Schizophr Res. 2012;140(1-3):37-40.

52. Nordentoft M, Jeppesen P, Petersen A, Thorup A, Ohlenschlaeger J, Krarup T, et al. The OPUS trial: a randomised multi-centre trial of integrated versus standard treatment for 547 first-episode psychotic patients. Schizophrenia Research. 2004;70:31.

53. Bertelsen M, Jeppesen P, Petersen L, Thorup A, Ohlenschlaeger J, le Quach P, et al. Five-year follow-up of a randomized multicenter trial of intensive early intervention vs standard treatment for patients with a first episode of psychotic illness: the OPUS trial. Arch Gen Psychiatry. 2008;65(7):762-71.

54. Craig TKJ, Garety PA, Power P, Rahaman N, Colbert S, Fornells-Ambrojo M, et al. The Lambeth Early Onset (LEO) Team: randomised controlled trial of the effectiveness of specialised care for early psychosis. British Medical Journal. 2004;329(Nov):1067.

55. Garety PA, Craig TK, Dunn G, Fornells-Ambrojo M, Colbert S, Rahaman N, et al. Specialised care for early psychosis: symptoms, social functioning and patient satisfaction: randomised controlled trial. Br J Psychiatry. 2006;188:37-45.

56. Addington J, Cornblatt BA, Cadenhead KS, Cannon TD, McGlashan TH, Perkins DO, et al. At clinical high risk for psychosis: outcome for nonconverters. The American journal of psychiatry. 2011;168(8):800-5.

57. Fusar-Poli P, Van Os J. Lost in transition: setting the psychosis threshold in prodromal research. Acta Psychiatr Scand. 2013;127(3):248-52.

58. Simon AE, Borgwardt S, Riecher-Rossler A, Velthorst E, de Haan L, Fusar-Poli P. Moving beyond transition outcomes: meta-analysis of remission rates in individuals at high clinical risk for psychosis. Psychiatry Res. 2013;209(3):266-72.

59. Yung AR, Nelson B, Yuen HP, Spiliotacopoulos D, Lin A, Simmons M, et al. Long term outcome in an ultra high risk (“prodromal”) group. Schizophrenia Bulletin. 2011;37(1):22-3.

60. Alvarez-Jimenez M, Gleeson JF, Henry LP, Harrigan SM, Harris MG, Killackey E, et al. Road to full recovery: longitudinal relationship between symptomatic remission and psychosocial recovery in first-episode psychosis over 7.5 years. Psychol Med. 2012;42(3):595-606.

61. Yung AR, Yuen HP, Berger G, Francey S, Hung TC, Nelson B, et al. Declining transition rate in ultra high risk (prodromal) services: dilution or reduction of risk? Schizophr Bull. 2007;33(3):673-81.

62. Tabak AG, Herder C, Rathmann W, Brunner EJ, Kivimaki M. Prediabetes: a high-risk state for diabetes development. Lancet. 2012;379(9833):2279-90.

63. Stone NJ, Robinson J, Lichtenstein AH, Merz CN, Blum CB, Eckel RH, et al. 2013 ACC/AHA Guideline on the Treatment of Blood Cholesterol to Reduce Atherosclerotic

Cardiovascular Risk in Adults: A Report of the American College of Cardiology/American Heart Association Task Force on Practice Guidelines. Circulation. 2013.

64. McGorry PD. Risk syndromes, clinical staging and DSM V: new diagnostic infrastructure for early intervention in psychiatry. Schizophr Res. 2010;120(1-3):49-53.

65. Cuthbert BN, Insel TR. Toward new approaches to psychotic disorders: the NIMH Research Domain Criteria project. Schizophr Bull. 2010;36(6):1061-2.

66. Cuthbert BN, Insel TR. Toward the future of psychiatric diagnosis: the seven pillars of RDoC. BMC medicine. 2013;11:126.

67. Insel T, Cuthbert B, Garvey M, Heinssen R, Pine DS, Quinn K, et al. Research domain criteria (RDoC): toward a new classification framework for research on mental disorders. Am J Psychiatry. 2010;167(7):748-51.

68. McGorry PD, Hickie IB, Yung AR, Pantelis C, Jackson HJ. Clinical staging of psychiatric disorders: A heuristic framework for choosing earlier, safer and more effective interventions. Australian and New Zealand Journal of Psychiatry Vol 40(8) Aug 2006, 616-622. 2006.

69. McGorry PD. Issues for DSM-V: clinical staging: a heuristic pathway to valid nosology and safer, more effective treatment in psychiatry. Am J Psychiatry. 2007;164(6):859-60.

70. McGorry P, van Os J. Redeeming diagnosis in psychiatry: timing versus specificity. Lancet. 2013;381(9863):343-5.

71. McGorry PD. Early clinical phenotypes, clinical staging, and strategic biomarker research: building blocks for personalized psychiatry. Biol Psychiatry. 2013;74(6):394-5.

72. Warner R. The prevention of schizophrenia: what interventions are safe and effective? Schizophr Bull. 2001;27(4):551-62.

73. Howes OD, Kapur S. The dopamine hypothesis of schizophrenia: version III--the final common pathway. Schizophr Bull. 2009;35(3):549-62.

74. Kaur T, Cadenhead KS. Treatment implications of the schizophrenia prodrome. Current topics in behavioral neurosciences. 2010;4:97-121.

75. Flatow J, Buckley P, Miller BJ. Meta-analysis of oxidative stress in schizophrenia. Biol Psychiatry. 2013;74(6):400-9.

76. Fineberg AM, Ellman LM. Inflammatory cytokines and neurological and neurocognitive alterations in the course of schizophrenia. Biol Psychiatry. 2013;73(10):951-66.

77. Miller BJ, Buckley P, Seabolt W, Mellor A, Kirkpatrick B. Meta-analysis of cytokine alterations in schizophrenia: clinical status and antipsychotic effects. Biol Psychiatry. 2011;70(7):663-71.

78. Kirkpatrick B, Miller BJ. Inflammation and schizophrenia. Schizophr Bull. 2013;39(6):1174-9.

79. Bitanihirwe BK, Woo TU. Oxidative stress in schizophrenia: an integrated approach. Neurosci Biobehav Rev. 2011;35(3):878-93.

80. Berk M, Kapczinski F, Andreazza AC, Dean OM, Giorlando F, Maes M, et al. Pathways underlying neuroprogression in bipolar disorder: focus on inflammation, oxidative stress and neurotrophic factors. Neurosci Biobehav Rev. 2011;35(3):804-17.

81. Dodd S, Maes M, Anderson G, Dean OM, Moylan S, Berk M. Putative neuroprotective agents in neuropsychiatric disorders. Prog Neuropsychopharmacol Biol Psychiatry. 2013;42:135-45.

82. Garety PA, Kuipers E, Fowler D, Freeman D, Bebbington PE. A cognitive model of the positive symptoms of psychosis. Psychol Med. 2001;31(2):189-95.

83. Morrison AP. The interpretation of intrusions in psychosis: An integrative cognitive approach to hallucinations and delusions. Behavioural and Cognitive Psychotherapy. 2001;29:257- 76.

84. Garety PA, Freeman D. Cognitive approaches to delusions: a critical review of theories and evidence. British Journal of Clinical Psychology. 1999;38:113-54.

85. French P. Model-driven psychological intervention to prevent onset of psychosis. Acta Psychiatrica Scandinavica Supplementum. 2002;106(413):18.

86. Phillips LJ, Francey SM. Changing PACE: Psychological interventions in the prepsychotic phase. In: Gleeson JFM, McGorry PD, editors. Psychological Interventions in Early Psychosis: A Treatment Handbook. Chichester: John Wiley & Sons; 2004. p. 23-39.

87. Morrison AP, editor. A casebook of cognitive therapy for psychosis. New York: Brunner-Routledge; 2002.

88. Lauber C, Nordt C, Falcato L, Rossler W. Lay recommendations on how to treat mental disorders. Soc Psychiatry Psychiatr Epidemiol. 2001;36(11):553-6.

89. Angermeyer MC, Matschinger H. Public attitude towards psychiatric treatment. Acta Psychiatr Scand. 1996;94(5):326-36.

90. Morrison AP, French P, Walford L, Lewis SW, Kilcommons A, Green J, et al. Cognitive therapy for the prevention of psychosis in people at ultra-high risk: Randomised controlled trial. British Journal of Psychiatry. 2004;185(4):291-7.

91. Bechdolf A, Wagner M, Veith V, Ruhrmann S, Pukrop R, Brockhaus-Dumke A, et al. Randomized controlled multicentre trial of cognitive behaviour therapy in the early initial prodromal state: effects on social adjustment post treatment. Early Intervention in Psychiatry. 2007;1(1):71-8.

92. van der Gaag M, Nieman DH, Rietdijk J, Dragt S, Ising HK, Klaassen RM, et al. Cognitive behavioral therapy for subjects at ultrahigh risk for developing psychosis: a randomized controlled clinical trial. Schizophr Bull. 2012;38(6):1180-8.

93. McGorry PD, Nelson B, Phillips LJ, Yuen HP, Francey SM, Thampi A, et al. Randomized controlled trial of interventions for young people at ultra-high risk of psychosis: twelve-month outcome. J Clin Psychiatry. 2013;74(4):349-56.

94. Yung AR, Phillips LJ, Nelson B, Francey SM, PanYuen H, Simmons MB, et al. Randomized controlled trial of interventions for young people at ultra high risk for psychosis: 6-month analysis. The Journal of clinical psychiatry. 2011;72(4):430-40.

95. Chen EYH, Lin J, Lee EHM, Chang WC, Chan SKW, Hui CLM. Yoga exercise for cognitive impairment in psychotic disorders. Schizophrenia Research. 2014;153, Supplement 1(0):S26.

96. Khoury B, Lecomte T, Gaudiano BA, Paquin K. Mindfulness interventions for psychosis: a meta-analysis. Schizophr Res. 2013;150(1):176-84.

97. Mesholam-Gately RI, Giuliano AJ, Goff D, Faraone SV, Seidman LJ. Neurocognition in first-episode schizophrenia: a meta-analytic review. Neuropsychology. 2009;23(3):315-36.

98. Cannon M, Caspi A, Moffitt TE, Harrington H, Taylor A, Murray RM, et al. Evidence for early-childhood, pan-developmental impairment specific to schizophreniform disorder: results from a longitudinal birth cohort. Archives of General Psychiatry. 2002;59(5):449-56.

99. Davidson M, Reichenberg A, Rabinowitz J, Weiser M, Kaplan Z, Mark M. Behavioral and intellectual markers for schizophrenia in apparently healthy male adolescents. The American Journal of Psychiatry. 1999;156(9):1328-35.

100. Brewer WJ, Wood SJ, Phillips LJ, Francey SM, Pantelis C, Yung AR, et al. Generalized and specific cognitive performance in clinical high-risk cohorts: a review highlighting potential vulnerability markers for psychosis. Schizophrenia Bulletin. 2006;32(3):538-55.

101. Eastvold AD, Heaton RK, Cadenhead KS. Neurocognitive deficits in the (putative) prodrome and first episode of psychosis. Schizophrenia research. 2007;93(1-3):266-77.

102. Pukrop R, Ruhrmann S, Schultze-Lutter F, Bechdolf A, Brockhaus-Dumke A, Klosterkötter J. Neurocognitive indicators for a conversion to psychosis: comparison of patients in a potentially initial prodromal state who did or did not convert to a psychosis. Schizophrenia Research. 2007;92(1-3):116-25.

103. Woodberry KA, Seidman LJ, Giuliano AJ, Verdi MB, Cook WL, McFarlane WR. Neuropsychological profiles in individuals at clinical high risk for psychosis: relationship to psychosis and intelligence. Schizophrenia research. 2010;123(2-3):188-98.

104. Brewer W, Francey S, Wood S, Jackson H, Pantelis C, Phillips L, et al. Memory impairments identified in people at ultra-high risk for psychosis who later develop first-episode psychosis. The American Journal of Psychiatry 2005;162(1):71-8.

105. Lencz T, Smith CW, McLaughlin D, Auther A, Nakayama E, Hovey L, et al. Generalized and specific neurocognitive deficits in prodromal schizophrenia. Biological Psychiatry. 2006;59(9):863-71.

106. Kim HS, Shin NY, Jang JH, Kim E, Shim G, Park HY, et al. Social cognition and neurocognition as predictors of conversion to psychosis in individuals at ultra high risk. Schizophrenia Research. 2011;doi:10.1016/j.schres.2011.04.023.

107. Becker HE, Nieman DH, Dingemans PM, van de Fliert JR, De Haan L, Linszen DH. Verbal fluency as a possible predictor for psychosis. European Psychiatry. 2010;25(2):105-10.

108. Riecher-Rössler A, Pflueger MO, Aston J, Borgwardt SJ, Brewer WJ, Gschwandtner U, et al. Efficacy of using cognitive status in predicting psychosis: a 7-year follow-up. Biological psychiatry. 2009;66(11):1023-30.

109. Mesholam-Gately RI, Giuliano AJ, Goff KP, Faraone SV, Seidman LJ. Neurocognition in first-episode schizophrenia: a meta-analytic review. Neuropsychology. 2009;23(3):315-36.

110. Pantelis C, Velakoulis D, McGorry PD, Wood SJ, Suckling J, Phillips LJ, et al. Neuroanatomical abnormalities before and after onset of psychosis: a cross-sectional and longitudinal MRI study. The Lancet. 2003;361:281-8.

111. Borgwardt SJ, McGuire PK, Aston J, Gschwandtner U, Pflüger MO, Stieglitz R, et al. Reductions in frontal, temporal and parietal volume associated with the onset of psychosis. Schizophrenia Research. 2008;106:108-14.

112. Job DE, Whalley H, Johnstone EC, Lawrie SM. Grey matter changes over time in high risk subjects developing schizophrenia. . Neuroimage;. 2005;25:1023-30.

113. Sun D, Phillips L, Velakoulis D, Yung A, McGorry PD, Wood SJ, et al. Progressive brain structural changes mapped as psychosis develops in 'at risk' individuals. Schizophr Res. 2009;108:85-92.

114. Takahashi T, Wood SJ, Yung AR, Soulsby B, McGorry PD, Suzuki M, et al. Progressive gray matter reduction of the superior temporal gyrus during transition to psychosis. Arch Gen Psychiatry. 2009;66(4):366-76.

115. Takahashi T, Wood SJ, Yung AR, Phillips L, Soulsby B, McGorry PD, et al. Insular cortex gray matter changes in individuals at ultra-high-risk of developing psychosis. Schizophr Res. 2009;111:94–102.

116. Walterfang M, McGuire PK, Yung A, Phillips L, Velakoulis D, Wood SJ, et al. White matter volume changes in people who develop psychosis. British Journal of Psychiatry. 2008;193(3):210-5.

117. Garey, L. J. *et al.* Reduced dendritic spine density on cerebral cortical pyramidal neurons in schizophrenia. *J. Neurol. Neurosurg. Psychiatry* **65,** 446–453 (1998).

118. Glantz, L. A. & Lewis, D. A. Decreased dendritic spine density on prefrontal cortical pyramidal neurons in schizophrenia. Arch. Gen. Psychiatry 57, 65–73 (2000).

119. Glausier, J. R. & Lewis, D. A. Dendritic spine pathology in schizophrenia. Neuroscience 251, 90–107 (2013).

120. O'Donovan MC, Craddock N, Norton N, Williams H, Peirce T, Moskvina V, et al. Identification of loci associated with schizophrenia by genome-wide association and follow-up. Nat Genet. 2008;40(9):1053-5.

121. Rare chromosomal deletions and duplications increase risk of schizophrenia. Nature. 2008;455(7210):237-41.

122. Schizophrenia Working Group of te Psychiatric Genomics Consortium. Biological insighs from 108 schizophrenia-assocaited genetic loci. Nature 511. 421-427 (2014)

123. Shi, J et al, Common variants on chromosome 6p22.1 are associated with schizophrenia. Nature. 2009 Aug 6;460(7256):753-7

124. Stefannson, H et al. Common varians conferring risk of schizophrenia. Nature 460, 744-747 (2009).

125. International Schizophrenia Consortium et al. Common polygenic variation contribues to risk of schizophrenia and bipolar disorder. Nature 460, 748-752 (2009),

126. Schizophrenia psychiatric Genome-Wide Association Study Consortium. Genome-wide association study identifies five new schizphreniai loci. Nature Genet. 43, 969-976 (2011).).

127. Sekar A, Bialas AR, de Rivera H, Davis A, Hammond TR, Kamitaki N*, et al*. Schizophrenia risk from complex variation of complement component 4. Nature. 2016 Feb 11;530 (7589):177-83.

128. Tandon R, Keshavan MS, Nasrallah HA. Schizophrenia, "just the facts" what we know in 2008. 2. Epidemiology and etiology. Schizophr Res. 2008;102(1-3):1-18..2 EU-GEI. Schizophrenia aetiology: do gene-environment interactions hold the key? Schizophrenia Research. 2008;102(1):21-6.3Sham P. Statistics in Human Genetics. London: Arnold; 1998.

129. EU-GEI. Schizophrenia aetiology: do gene-environment interactions hold the key? Schizophrenia Research. 2008;102(1):21-6.

130. Sham P. Statistics in Human Genetics. London: Arnold; 1998

131. Lei H, Nahum-Shani I, Lynch K, Oslin D, Murphy SA. A "SMART" design for building individualized treatment sequences. Annu Rev Clin Psychol. 2012;8:21-48

132. Lavori PW, Rush AJ, Wisniewski SR, Alpert J, Fava M, Kupfer DJ, et al. Strengthening clinical effectiveness trials: equipoise-stratified randomization. Biol Psychiatry. 2001;50(10):792-801.

133. McGorry P, Bates T, Birchwood M. Designing youth mental health services for the 21st century: examples from Australia, Ireland and the UK. The British Journal of Psychiatry. 2013;202(s54):s30-s5.

134. McGorry PD. The specialist youth mental health model: strengthening the weakest link in the public mental health system. Med J Aust. 2007;187(7 Suppl):S53-6.

135. McGorry PD, Tanti C, Stokes R, Hickie IB, Carnell K, Littlefield LK, et al. headspace: Australia's National Youth Mental Health Foundation - where young minds come first. Medical Journal of Australia. 2007;187(7):S68.

136. Scott EM, Hermens DF, Glozier N, Naismith SL, Guastella AJ, Hickie IB. Targeted primary care-based mental health services for young Australians. Med J Aust. 2012;196:136-40.

137. Rickwood D, Telford N, Parker A, Tanti C, P.D. M. headspace - Australian innovation in youth mental health care: who are the clients and why are they presenting to headspace centres? . MJA in press;epub 13 January 2014.

138. Cornblatt BA, Auther AM, Niendam T, Smith CW, Zinberg J, Bearden CE, et al. Preliminary findings for two new measures of social and role functioning in the prodromal phase of schizophrenia. Schizophr Bull. 2007;33(3):688-702.

139. Yung AR, Yuen HP, McGorry PD, Phillips LJ, Kelly D, Dell'Olio M, et al. Mapping the onset of psychosis: the Comprehensive Assessment of At-Risk Mental States. The Australian and New Zealand journal of psychiatry. 2005;39(11-12):964-71

140.Goldman H, Skodol A, Lave T. Revising Axis V for DSM-IV: A review of measures of social functioning. American Journal of Psychiatry. 1992;149:1148-56.

141. Ising HK, Veling W, Loewy RL, Rietveld MW, Rietdijk J, Dragt S, et al. The Validity of the 16-Item Version of the Prodromal Questionnair16e (PQ-16) to Screen for Ultra High Risk of Developing Psychosis in the General Help-Seeking Population. Schizophrenia bulletin. 2012.

142. Phillips LJM, Leicester SBM, O'Dwyer LEB, Francey SMM, Koutsogiannis JMF, Abdel-Baki AMF, et al. The PACE Clinic: Identification and Management of Young People at "Ultra" High Risk of Psychosis. Journal of Psychiatric Practice September. 2002;8(5):255-69.

143. Yung AR, Nelson B. Young people at ultra high risk for psychosis: a research update. Early Interv Psychiatry. 2011;5 Suppl 1:52-7.

144. Phillips LJ, Nelson B, Yuen HP, Francey SM, Simmons M, Stanford C, et al. Randomized controlled trial of interventions for young people at ultra-high risk of psychosis: study design and baseline characteristics. The Australian and New Zealand journal of psychiatry. 2009;43(9):818-29.

145. Rietdijk J, Klaassen R, Ising H, Dragt S, Nieman DH, van de Kamp J, et al. Detection of people at risk of developing a first psychosis: comparison of two recruitment strategies. Acta psychiatrica Scandinavica. 2012.

146. Lim CM, Kim SW, Park JY, Kim C, Yoon SH, Lee JK. Fluoxetine affords robust neuroprotection in the postischemic brain via its anti-inflammatory effect. Journal of neuroscience research. 2009;87(4):1037-45.

147. van der Gaag M, Schutz C, Ten Napel A, Landa Y, Delespaul P, Bak M, et al. Development of the Davos assessment of cognitive biases scale (DACOBS). Schizophr Res. 2013;144(1-3):63-71

148. Bernstein DP, Ahluvalia T, Pogge D, Handelsman L. Validity of the Childhood Trauma Questionnaire in an adolescent psychiatric population. Journal of the American Academy of Child & Adolescent Psychiatry. 1997;36:340-8

149. Overall J, Gorham D. The Brief Psychiatric Rating Scale. Psychological Reports. 1962;10:799-812.

150 Andreasen NC. The scale for the assessment of negative symptoms (SANS). Iowa City: The University of Iowa; 1983 1983.

151. Montgomery SA, Asberg M. A new depression scale designed to be sensitive to change. Br J Psychiatry. 1979;134:382-9.

152. Group WAW. The Alcohol, Smoking and Substance Involvement Screening Test (ASSIST): development, reliability and feasibility. Addiction. 2002;97(9):1183-94.

153. Moller P, Baklund, L., Rosse, R., Skjelstad, D.V. Anomalous self-experience as an add-on perspective to the UHR criteria for psychosis in late childhood and adolescence: development of a two-perspective screening instrument. Early Intervention in Psychiatry. 2014;8(suppl 1):132

154. First MB, Spitzer RL, Gibbon M, Williams JBW. Structured Clinical Interview for DSM-IV Axis I Disorders - Patient edition (SCID-I/P, Version 2.0). New York: Bimetrics Research Department; 1996.

155. Fusar-Poli P, Nelson B, Valmaggia L, Yung AR, McGuire PK. Comorbid depressive and anxiety disorders in 509 individuals with an at-risk mental state: impact on psychopathology and transition to psychosis. Schizophr Bull. 2014;40(1):120-31.

156. Group EPGW. Australian Clinical Guidelines for Early Psychosis. 2nd ed. Melbourne: Orygen Youth Health; 2010.

157. Group IEPAW. International clinical practice guidelines for early psychosis. British Journal of Psychiatry. 2005;187(suuplement 48):s120-s4

158. initiative btnd. Clinical practice guidelines: Depression in adolescents and young adults. Melbourne2010.

159. Parker AG, Hetrick SE, Jorm AF, Yung AR, McGorry PD, Mackinnon A, et al. The effectiveness of simple psychological and exercise interventions for high prevalence mental health problems in young people: a factorial randomised controlled trial. Trials. 2011;12:76.

160. McGorry PD, Yung A, Phillips L. Ethics and Early Intervention in Psychosis: Keeping up the pace and staying in step. Schizophr Res. 2001;51(1):17-29.

161. Ratheesh A, Cotton SM, Davey CG, Adams S, Bechdolf A, Macneil C, Berk M, McGorry PD. Ethical considerations in preventive interventions for bipolar disorder. Erly Interv Psyichiatry. 2017;11(2):104-112.

162. Erickson, JA, et.al. Genetic Testing of Children for Predisposition to Mood Disorders: Anticipating the Clinical Issues. J Genet Counsel (2014) 23:566–577, DOI 10.1007/s10897-014-9710-y

163. Davison, J. and J. Scott (2017). "Should we intervene at stage 0? A qualitative study of attitudes of asymptomatic youth at increased risk of developing bipolar disorders and parents with established disease." Early Interv Psychiatry. https://doi.org/10.1111/eip.12421
