# Supplementary material for: A Sequential Adaptive Intervention Strategy Targeting Remission and Functional Recovery in Young People at Ultrahigh Risk of Psychosis: The Staged Treatment in Early Psychosis (STEP) Sequential Multiple Assignment Randomized Trial
Source: JAMA Psychiatry. 2023 Jun 28;80(9):875–85. doi: 10.1001/jamapsychiatry.2023.1947 (PMC10308298; doi:10.1001/jamapsychiatry.2023.1947)
Supplement: Supplement 2. — eMethods eTable 1. Reasons for treatment discontinuation (%) eTable 2. Pearson correlation between baseline DACOBS total and 6-month outcomes and between change in DACOBS total and change in outcomes (Step 1 non-remitters) eTable 3. 12-month outcomes of the fast-fail group eTable 4. General linear model analysis comparing SPS and monitoring (Step 1 remitters) at 6 and 12 months with data handled using multiple imputation eTable 5. General linear model analysis comparing SPS and monitoring (Step 2 remitters) at 12 months with data handled using multiple imputation eTable 6. Effect sizes (Cohen’s d) at 6 and 12 months across the entire sample eTable 7. Complete-case and per-protocol analysis comparing CBCM and SPS at 6 months eTable 8. Complete-case and per-protocol analysis comparing CBCM+fluoxetine and CBCM+placebo at 12 months eTable 9. Complete-case and per-protocol analysis of remission rates eTable 10. Complete-case and per-protocol analysis comparing relapse rates between SPS and monitoring (Step 1 remitters) at 6 and 12 months eTable 11. Complete-case and per-protocol analysis comparing relapse rates between SPS and monitoring (Step 2 remitters) at 12 months using logistic regression eTable 12. Complete-case and per-protocol analysis comparing SPS and monitoring (Step 1 remitters) at 6 months eTable 13. Complete-case and per-protocol analysis comparing SPS and monitoring (Step 1 remitters) at 12 months eTable 14. Complete-case and per-protocol analysis comparing SPS and monitoring (Step 2 remitters) at 12 months eTable 15. Kaplan-Meier estimated transition rates (complete-case and per-protocol analysis) eTable 16. Treatment adherence rates eTable 17. Adverse events eFigure 1. Changes in mean symptom and functioning scores from baseline to the end of Step 2 eFigure 2. Changes in mean CAARMS severity scores from baseline to the end of Step 2 (panel A) and Step 3 (panel B) eFigure 3. Changes in mean CAARMS frequency scores from baseline to the end of Step 2 (pane [file jamapsychiatry-e231947-s002.pdf]

Supplemental Online Content

McGorry PD, Mei C, Amminger GP, et al. A sequential adaptive intervention strategy targeting remission and functional recovery in young people at ultrahigh risk of psychosis: the Staged Treatment in Early Psychosis (STEP) sequential multiple assignment study. *JAMA Psychiatry*. Published online June 28, 2023. doi:10.1001/jamapsychiatry.2023.1947

eMethods
eTable 1. Reasons for treatment discontinuation (%)
eTable 2. Pearson correlation between baseline DACOBS total and 6-month outcomes and between change in DACOBS total and change in outcomes (Step 1 non-remitters)
eTable 3. 12-month outcomes of the fast-fail group
eTable 4. General linear model analysis comparing SPS and monitoring (Step 1 remitters) at 6 and 12 months with data handled using multiple imputation
eTable 5. General linear model analysis comparing SPS and monitoring (Step 2 remitters) at 12 months with data handled using multiple imputation
eTable 6. Effect sizes (Cohen's d) at 6 and 12 months across the entire sample
eTable 7. Complete-case and per-protocol analysis comparing CBCM and SPS at 6 months
eTable 8. Complete-case and per-protocol analysis comparing CBCM+fluoxetine and CBCM+placebo at 12 months
eTable 9. Complete-case and per-protocol analysis of remission rates
eTable 10. Complete-case and per-protocol analysis comparing relapse rates between SPS and monitoring (Step 1 remitters) at 6 and 12 months
eTable 11. Complete-case and per-protocol analysis comparing relapse rates between SPS and monitoring (Step 2 remitters) at 12 months using logistic regression
eTable 12. Complete-case and per-protocol analysis comparing SPS and monitoring (Step 1 remitters) at 6 months
eTable 13. Complete-case and per-protocol analysis comparing SPS and monitoring (Step 1 remitters) at 12 months
eTable 14. Complete-case and per-protocol analysis comparing SPS and monitoring (Step 2 remitters) at 12 months
eTable 15. Kaplan-Meier estimated transition rates (complete-case and per-protocol analysis)
eTable 16. Treatment adherence rates
eTable 17. Adverse events
eFigure 1. Changes in mean symptom and functioning scores from baseline to the end of Step 2
eFigure 2. Changes in mean CAARMS severity scores from baseline to the end of Step 2 (panel A) and Step 3 (panel B)
eFigure 3. Changes in mean CAARMS frequency scores from baseline to the end of Step 2 (panel A) and Step 3 (panel B)

This supplemental material has been provided by the authors to give readers additional information about their work.

© 2023 McGorry PD et al. *JAMA Psychiatry.*

eMethods 1. SPS and CBCM treatments

CBCM and SPS were both manualized therapies based on treatment models developed at our specialist UHR clinic, the PACE clinic, and used in several of our previous RCTs.[1-3] Training in SPS and CBCM was provided by the study team to clinical study staff (frontline primary care psychologists and other allied health staff) via these written manuals and in-person group training sessions. 6-monthly ‘top up’ training sessions were provided throughout the duration of the study and group therapy supervision was held with each recruiting clinic every 2 weeks. Two dedicated clinical psychologists (BN, JR) were also available on an as-needs basis for treatment-related issues that emerged during the intervention period.

[1] McGorry PD, Nelson B, Markulev C, et al. Effect of ω-3 polyunsaturated fatty acids in young people at ultrahigh risk for psychotic disorders: the NEURAPRO randomized clinical trial. JAMA Psychiatry. 2017;74(1):19-27.

[2] McGorry PD, Nelson B, Phillips LJ, et al. Randomized controlled trial of interventions for young people at ultra-high risk of psychosis: twelve-month outcome. J Clin Psychiatry. 2013;74(4):349-56.

[3] Hartmann JA, McGorry PD, Schmidt SJ, et al. Opening the black box of cognitive-behavioural case management in clients with ultra-high risk for psychosis. Psychother Psychosom. 2017;86(5):292-299.

© 2023 McGorry PD et al. *JAMA Psychiatry.*

eMethods 2. Information regarding the application of multiple imputation in this study

Multiple imputation is an approach in which imputation of the missing data are carried out a number of times to create a number of complete data sets. These data sets are then analyzed separately and the results from these data sets are then combined in a statistically appropriate manner.

To apply multiple imputation, the usual assumption is that the missing data are missing at random (MAR). To investigate the validity of this assumption, we examined the reasons for the missing data at 6 months, which was the time-point for the study's primary objective. 36.3% of the cases had missing 6-month data, of which 9.7% were due to known reasons which did not appear to cause bias in the analysis (moved out of area, withdrew due to time commitment, no longer wanting support and did not want to take medication). 2.0% were due to transition to psychosis or deterioration of mental state. These missing data could be 'explained' or predicted in the imputation process using baseline data as well as the non-missing data of other transitioned cases. The remaining 24.6% withdrew due to unknown reasons. These cases were fairly evenly distributed among the 4 treatment regimes at Step 2, i.e., before 6 months (25.0%, 23.3%, 28.9% and 21.2%, respectively for the 4 regimes) and there was no reason to believe that these cases would cause bias in the analysis. Based on the above reasoning, our judgement was that it was reasonable to assume that the missing data were MAR, i.e., the likelihood of observing a value is independent of the value itself, given the data that one has observed. There were missing data at other time-points apart from 6 months. We made the pragmatic assumption that MAR also applied to these missing data.

We applied multiple imputation by using the R package mice[4] and miceadds[5] which conducts multivariate imputation by chained equations. The predictors used in the imputation included age, gender, child trauma score and the longitudinal measures of functioning, general psychopathology, negative symptoms, positive symptoms, depression and quality of life. The longitudinal structure of the data was taken into account in the imputation. The number of imputations used was 100.

An additional point to note is that treatment allocations in this study were dependent on remission status. For many of the drop-outs, their remission status after a particular step could not be determined because the data relevant to the remission criteria were included in the missing data. After data imputation, the missing remission status became determinable. In other words, the missing remission status were also imputed in the process. In turn, treatment allocations for the drop-outs could then proceed in accordance with the imputed remission status in the intention-to-treat analysis.

[4] van Buuren S & Groothuis-Oudshoorn K. mice: Multivariate Imputation by Chained Equations in R. Journal of Statistical Software. 2011 45(3):1-67. https://www.jstatsoft.org/v45/i03/.

[5] Robitzsch A & Grund S. (2022). miceadds: Some Additional Multiple Imputation Functions, Especially for 'mice'. R package version 3.12-26. https://CRAN.R-project.org/package=miceadds

© 2023 McGorry PD et al. *JAMA Psychiatry.*

eTable 1. Reasons for treatment discontinuation (%)

	Step 1	Step 2		Step 3	
	n=61	Non-remitter arm, n=116	Remitter arm, n=18	Non-remitter arm, n=42	Remitter arm, n=11
**Lost to follow-up**	49.2	25.9	50.0	11.9	18.2
**Deterioration in symptoms; more targeted treatment required; transition to psychosis**	16.4	11.2	16.7	16.7	0
**Did not want to start medication in Step 3**	0	27.6	0	26.2	0
**Commenced medication prior to Step 3**	11.5	6.9	5.6	0	0
**Elected to cease study medication**	0	0	0	19.0	0
**Moved out of area**	3.3	5.2	0	2.4	9.1
**No longer wanting support**	6.6	7.8	0	0	27.3
**Withdrew due to time commitment**	4.9	3.4	0	2.4	0
**Elected to pursue different therapy**	0	3.4	11.1	2.4	36.4
**Other**	3.3	4.3	0	2.4	0
**No reason given**	4.9	4.3	16.7	16.7	9.1

© 2023 McGorry PD et al. *JAMA Psychiatry.*

eTable 2. Pearson correlation between baseline DACOBS total and 6-month outcomes and between change in DACOBS total and change in outcomes (Step 1 non-remitters)

	Baseline DACOBS total and 6-month outcome				Change in DACOBS total and in outcomes[1]			
	n	Correlation	95% CI	P value	n	Correlation	95% CI	P value
GF: Social	180	-0.12	-0.26–0.03	0.120	174	-0.10	-0.24–0.05	0.202
GF: Role	180	-0.13	-0.27–0.01	0.076	174	-0.08	-0.23–0.07	0.296
AQoL total	172	-0.27	-0.40–-0.12	<0.001	170	-0.38	-0.50–-0.25	<0.001
BPRS total	176	0.15	0.01–0.29	0.043	169	0.31	0.17–0.44	<0.001
SANS total	176	0.26	0.11–0.39	0.001	169	0.14	-0.01–0.28	0.073
MADRS total	180	0.14	-0.01–0.28	0.064	171	0.26	0.12–0.40	<0.001
SOFAS	181	-0.18	-0.32–-0.03	0.016	174	-0.10	-0.25–0.05	0.176

[1] Change = 6-month score minus baseline score.

AQoL, Assessment of Quality of Life; BPRS, Brief Psychiatric Rating Scale; GF: Global Functioning; MADRS, Montgomery-Åsberg Depression Rating Scale; SANS, Scale for the Assessment of Negative Symptoms; SOFAS, Social and Occupational Functioning Assessment Scale.

© 2023 McGorry PD et al. *JAMA Psychiatry.*

eTable 3. 12-month outcomes of the fast-fail group

	n	Mean (SD)
**GF: Social**	24	6.4 (1.6)
**GF: Role**	25	6.8 (1.6)
**AQoL total**	24	61.6 (13.8)
**BPRS total**	25	42.4 (9.8)
**SANS total**	25	15.6 (12.2)
**MADRS total**	25	20.8 (11.4)
**SOFAS**	25	62.8 (13.1)

AQoL, Assessment of Quality of Life; BPRS, Brief Psychiatric Rating Scale; GF: Global Functioning; MADRS, Montgomery-Åsberg Depression Rating Scale; SANS, Scale for the Assessment of Negative Symptoms; SOFAS, Social and Occupational Functioning Assessment Scale.

© 2023 McGorry PD et al. *JAMA Psychiatry.*

eTable 4. General linear model analysis comparing SPS and monitoring (Step 1 remitters) at 6 and 12 months with missing data handled using multiple imputation

		Baseline	Month 6 (mean n[1]: 15 SPS; 14 monitoring)			Month 12 (mean n[1]: 14 SPS; 15 monitoring)		
		Mean (SE)	Mean (SE)	Effect size	P value	Mean (SE)	Effect size	P value
GF: Social	Monitoring	7.0 (0.26)	7.4 (0.37)	-0.82	0.09	6.6 (0.71)	-0.26	0.61
	SPS	6.6 (0.37)	6.2 (0.48)			6.2 (0.72)		
GF: Role	Monitoring	6.8 (0.43)	7.0 (0.49)	-0.05	0.91	6.6 (0.80)	0.12	0.80
	SPS	6.8 (0.42)	6.9 (0.46)			6.9 (0.68)		
AQoL total	Monitoring	64.5 (2.91)	70.6 (2.96)	-0.28	0.50	70.6 (4.29)	-0.11	0.80
	SPS	68.2 (3.45)	69.5 (4.62)			71.3 (5.03)		
BPRS total	Monitoring	37.8 (1.89)	35.2 (2.03)	0.09	0.83	36.8 (3.01)	0.05	0.89
	SPS	38.5 (2.14)	36.1 (2.58)			37.7 (3.29)		
SANS total	Monitoring	13.8 (2.42)	12.5 (3.28)	0.05	0.89	14.2 (4.04)	-0.06	0.91
	SPS	16.8 (3.10)	15.8 (4.66)			15.2 (3.57)		
MADRS total	Monitoring	14.9 (2.82)	10.1 (2.07)	0.48	0.25	13.1 (3.64)	0.10	0.82
	SPS	16.8 (2.95)	15.1 (3.42)			14.9 (4.00)		
SOFAS	Monitoring	61.0 (3.36)	70.5 (2.79)	-0.91	0.03	63.8 (6.42)	-0.19	0.64
	SPS	57.3 (2.76)	59.5 (3.77)			60.8 (4.45)		
CAARMS, Severity								
UTC	Monitoring	1.1 (0.38)	0.5 (0.27)	0.38	0.40	1.1 (0.56)	-0.13	0.80
	SPS	1.4 (0.52)	0.9 (0.45)			0.9 (0.57)		
NBI	Monitoring	1.5 (0.43)	0.8 (0.39)	0.11	0.80	0.9 (0.53)	0.35	0.44
	SPS	1.7 (0.45)	0.9 (0.45)			1.6 (0.61)		
PA	Monitoring	2.5 (0.46)	0.9 (0.46)	0.36	0.38	1.9 (0.56)	-0.24	0.58
	SPS	2.4 (0.44)	1.6 (0.51)			1.4 (0.59)		
DS	Monitoring	1.7 (0.38)	1.0 (0.31)	0.14	0.72	1.0 (0.44)	0.42	0.36
	SPS	1.5 (0.38)	1.2 (0.46)			1.6 (0.58)		
CAARMS, Frequency								

© 2023 McGorry PD et al. *JAMA Psychiatry.*

**UTC**	**Monitoring**	1.3 (0.44)	0.7 (0.35)	0.17	0.70	1.0 (0.52)	0.06	0.90
	**SPS**	1.0 (0.38)	0.9 (0.42)			1.1 (0.60)		
**NBI**	**Monitoring**	1.8 (0.50)	1.2 (0.56)	-0.06	0.89	1.3 (0.64)	0.18	0.70
	**SPS**	2.2 (0.58)	1.2 (0.50)			1.8 (0.62)		
**PA**	**Monitoring**	2.2 (0.38)	0.7 (0.36)	0.68	0.10	1.6 (0.55)	-0.25	0.57
	**SPS**	2.5 (0.49)	1.7 (0.59)			1.1 (0.54)		
**DS**	**Monitoring**	1.8 (0.46)	1.7 (0.44)	0.10	0.79	1.5 (0.60)	0.26	0.57
	**SPS**	1.9 (0.55)	1.9 (0.66)			2.0 (0.73)		

AQoL, Assessment of Quality of Life; BPRS, Brief Psychiatric Rating Scale; CAARMS, Comprehensive Assessment of At-Risk Mental States; DS, Disorganized speech; GF: Global Functioning; MADRS, Montgomery-Åsberg Depression Rating Scale; NBI, Non-bizarre ideas; PA, Perceptual abnormalities; SANS, Scale for the Assessment of Negative Symptoms; SPS, support and problem solving; SOFAS, Social and Occupational Functioning Assessment Scale; UTC, Unusual thought content.
P value comparing monitoring and SPS with baseline score as a covariate.
[1] The mean sample sizes for each group over the multiple imputations.

© 2023 McGorry PD et al. *JAMA Psychiatry.*

eTable 5. General linear model analysis comparing SPS and monitoring (Step 2 remitters) at 12 months with data handled using multiple imputation

		Baseline	Month 12 (mean n[3]: 15 SPS; 17 monitoring)			
		Mean (SE)	Mean (SE)	Effect size	P value[1]	P value[2]
GF: Social	Monitoring	7.0 (0.29)	7.3 (0.56)	-0.41	0.45	0.95
	SPS	6.4 (0.33)	6.4 (0.63)			
GF: Role	Monitoring	6.5 (0.48)	7.0 (0.59)	-0.06	0.89	0.37
	SPS	6.6 (0.43)	7.0 (0.69)			
AQoL total	Monitoring	56.8 (2.68)	62.5 (3.32)	0.04	0.94	0.73
	SPS	59.3 (2.95)	64.8 (3.77)			
BPRS total	Monitoring	40.2 (1.76)	37.9 (2.73)	<0.01	0.998	0.66
	SPS	40.3 (1.66)	38.1 (2.86)			
SANS total	Monitoring	14.1 (2.73)	10.7 (3.31)	0.21	0.63	0.43
	SPS	16.0 (2.88)	13.4 (3.62)			
MADRS total	Monitoring	20.9 (2.23)	16.1 (2.61)	0.28	0.53	0.59
	SPS	19.5 (2.25)	18.1 (3.28)			
SOFAS	Monitoring	61.6 (3.23)	69.0 (3.35)	-0.29	0.51	0.38
	SPS	58.0 (3.12)	65.2 (3.51)			
CAARMS, Severity						
UTC	Monitoring	1.8 (0.46)	0.7 (0.43)	0.23	0.646	0.489
	SPS	2.3 (0.43)	1.1 (0.48)			
NBI	Monitoring	2.8 (0.39)	1.5 (0.51)	0.06	0.896	0.816
	SPS	2.5 (0.45)	1.5 (0.53)			
PA	Monitoring	2.3 (0.51)	1.7 (0.59)	0.21	0.640	0.774
	SPS	3.2 (0.37)	2.1 (0.60)			
DS	Monitoring	1.3 (0.30)	1.1 (0.41)	0.18	0.687	0.747
	SPS	1.5 (0.33)	1.4 (0.44)			

© 2023 McGorry PD et al. *JAMA Psychiatry.*

**CAARMS, Frequency**						
**UTC**	**Monitoring**	1.9 (0.47)	1.0 (0.49)	0.11	0.807	0.797
	**SPS**	2.2 (0.40)	1.1 (0.56)			
**NBI**	**Monitoring**	3.1 (0.43)	2.2 (0.60)	-0.18	0.696	0.758
	**SPS**	2.4 (0.43)	1.8 (0.54)			
**PA**	**Monitoring**	1.7 (0.40)	1.3 (0.46)	0.12	0.802	0.934
	**SPS**	2.9 (0.42)	1.4 (0.49)			
**DS**	**Monitoring**	2.2 (0.52)	2.0 (0.61)	0.05	0.913	0.641
	**SPS**	2.3 (0.53)	2.0 (0.62)			

AQoL, Assessment of Quality of Life; BPRS, Brief Psychiatric Rating Scale; CAARMS, Comprehensive Assessment of At-Risk Mental States; DS, Disorganized speech; GF: Global Functioning; MADRS, Montgomery-Åsberg Depression Rating Scale; NBI, Non-bizarre ideas; PA, Perceptual abnormalities; SANS, Scale for the Assessment of Negative Symptoms; SPS, support and problem solving; SOFAS, Social and Occupational Functioning Assessment Scale; UTC, Unusual thought content.

[1] Comparing monitoring and SPS with baseline score as a covariate.

[2] Interaction between the factors SPS vs. cognitive-behavioral case management (CBCM) and monitoring vs. SPS with baseline score as a covariate.

[3] The mean sample sizes for each group over the multiple imputations.

© 2023 McGorry PD et al. *JAMA Psychiatry.*

eTable 6. Effect sizes (Cohen's d) at 6 and 12 months across the entire sample

	6 months	12 months
**Global Functioning: Social**	0.02	0.04
**Global Functioning: Role**	0.14	0.10
**BPRS total**	0.28	0.27
**SANS total**	0.11	0.16
**MADRS total**	0.37	0.38
**SOFAS**	0.29	0.25
**AQoL total**	0.43	0.57

AQoL, Assessment of Quality of Life; BPRS, Brief Psychiatric Rating Scale; MADRS, Montgomery-Åsberg Depression Rating Scale; SANS, Scale for the Assessment of Negative Symptoms; SOFAS, Social and Occupational Functioning Assessment Scale.

© 2023 McGorry PD et al. *JAMA Psychiatry.*

eTable 7. Complete-case and per-protocol analysis comparing CBCM and SPS at 6 months

		Complete-case analysis					Per-protocol analysis				
			Baseline	Month 6				Baseline	Month 6		
		n	Mean (SE)	Mean (SE)	Effect size	P value	n	Mean (SE)	Mean (SE)	Effect size	P value
**GF-S**	**SPS**	97	6.6 (0.13)	6.8 (0.12)	-0.21	0.140	33	6.6 (0.20)	6.7 (0.16)	-0.14	0.504
	**CBCM**	95	6.5 (0.11)	6.5 (0.13)			63	6.5 (0.15)	6.5 (0.17)		
**GF-R**	**SPS**	97	6.1 (0.17)	6.6 (0.15)	-0.14	0.334	33	6.1 (0.29)	6.4 (0.29)	-0.01	0.973
	**CBCM**	95	6.5 (0.15)	6.6 (0.18)			63	6.5 (0.18)	6.7 (0.22)		
**AQoL total**	**SPS**	86	53.7 (1.29)	58.3 (1.26)	0.05	0.757	30	51.7 (2.29)	56.4 (2.42)	0.03	0.882
	**CBCM**	84	53.0 (1.26)	58.2 (1.46)			57	51.8 (1.52)	56.7 (1.78)		
**BPRS total**	**SPS**	95	45.7 (0.86)	41.6 (0.85)	0.04	0.782	31	46.1 (1.31)	42.4 (1.45)	-0.05	0.831
	**CBCM**	91	45.0 (0.86)	41.6 (0.97)			59	44.8 (1.06)	41.2 (1.15)		
**SANS total**	**SPS**	96	18.6 (1.14)	16.0 (1.09)	0.22	0.140	33	19.9 (1.85)	16.9 (1.96)	0.28	0.196
	**CBCM**	91	19.2 (1.24)	18.5 (1.37)			60	18.3 (1.51)	18.6 (1.79)		
**MADRS total**	**SPS**	96	23.3 (0.96)	19.3 (0.99)	-0.10	0.494	32	25.5 (1.64)	20.7 (1.93)	-0.13	0.553
	**CBCM**	92	24.4 (0.94)	18.9 (1.12)			61	24.6 (1.10)	19.0 (1.33)		
**SOFAS**	**SPS**	98	57.1 (1.22)	62.1 (1.17)	-0.14	0.343	33	57.4 (2.12)	60.5 (1.92)	0.04	0.854
	**CBCM**	95	57.4 (1.13)	60.8 (1.30)			63	57.7 (1.47)	61.0 (1.61)		
**DACOBS total**	**SPS**	89	169.8 (3.2)	162.6 (3.2)	-0.12	0.424					
	**CBCM**	85	169.0 (3.2)	159.0 (3.9)							
**CAARMS, Severity**											
**UTC**	**SPS**	98	3.0 (0.16)	1.9 (0.20)	-0.08	0.584	33	3.2 (0.31)	2.4 (0.36)	-0.27	0.226
	**CBCM**	93	2.6 (0.19)	1.6 (0.20)			63	2.6 (0.23)	1.6 (0.25)		
**NBI**	**SPS**	98	3.3 (0.16)	2.5 (0.18)	-0.14	0.353	33	3.2 (0.31)	2.9 (0.30)	-0.34	0.120
	**CBCM**	93	3.0 (0.16)	2.2 (0.18)			63	3.0 (0.19)	2.3 (0.23)		
**PA**	**SPS**	98	3.2 (0.16)	2.8 (0.17)	-0.07	0.630	33	2.8 (0.35)	2.8 (0.32)	-0.13	0.544
	**CBCM**	93	3.3 (0.14)	2.7 (0.19)			63	3.2 (0.18)	2.8 (0.22)		
**DS**	**SPS**	98	1.7 (0.13)	1.5 (0.13)	0.19	0.185	33	1.5 (0.21)	1.4 (0.21)	0.23	0.290

© 2023 McGorry PD et al. *JAMA Psychiatry.*

	**CBCM**	93	1.7 (0.12)	1.7 (0.12)			63	1.8 (0.14)	1.7 (0.15)		
**CAARMS, Frequency**											
**UTC**	**SPS**	97	2.8 (0.16)	1.7 (0.17)	-0.02	0.905	32	3.0 (0.31)	2.2 (0.31)	-0.28	0.213
	**CBCM**	93	2.3 (0.18)	1.5 (0.20)			63	2.4 (0.23)	1.4 (0.23)		
**NBI**	**SPS**	97	3.4 (0.17)	2.7 (0.18)	-0.06	0.706	32	3.3 (0.31)	3.2 (0.31)	-0.26	0.230
	**CBCM**	92	3.0 (0.16)	2.5 (0.20)			63	3.1 (0.18)	2.7 (0.25)		
**PA**	**SPS**	97	2.4 (0.14)	2.5 (0.17)	-0.14	0.360	33	2.1 (0.29)	2.5 (0.31)	-0.18	0.425
	**CBCM**	92	2.9 (0.14)	2.4 (0.19)			62	2.7 (0.17)	2.4 (0.22)		
**DS**	**SPS**	98	2.7 (0.20)	2.6 (0.21)	-0.01	0.930	33	2.5 (0.35)	2.5 (0.39)	-0.04	0.866
	**CBCM**	93	2.6 (0.19)	2.5 (0.19)			63	2.7 (0.21)	2.6 (0.23)		

AQoL, Assessment of Quality of Life; BPRS, Brief Psychiatric Rating Scale; CAARMS, Comprehensive Assessment of At-Risk Mental States; CBCM, cognitive-behavioral case management; DACOBS, Davos Assessment of Cognitive Biases Scale; DS, Disorganized speech; GF: Global Functioning; MADRS, Montgomery-Åsberg Depression Rating Scale; NBI, Non-bizarre ideas; PA, Perceptual abnormalities; SANS, Scale for the Assessment of Negative Symptoms; SPS, support and problem solving; SOFAS, Social and Occupational Functioning Assessment Scale
P value comparing SPS and CBCM using general linear model analysis with baseline score as a covariate; UTC, Unusual thought content.

© 2023 McGorry PD et al. *JAMA Psychiatry.*

eTable 8. Complete-case and per-protocol analysis comparing CBCM+fluoxetine and CBCM+placebo at 12 months

		Complete-case analysis						Per-protocol analysis					
			Baseline	Month 12					Baseline	Month 12			
		n	Mean (SE)	Mean (SE)	Effect size	P value[1]	P value[2]	n	Mean (SE)	Mean (SE)	Effect size	P value[1]	P value[2]
**GF: Social**	**Placebo**	48	6.4 (0.20)	6.8 (0.18)	-0.24	0.253	0.621	9	5.9 (0.31)	6.3 (0.53)	-0.28	0.581	0.373
	**Fluoxetine**	43	6.6 (0.19)	6.6 (0.21)				12	6.8 (0.35)	6.8 (0.51)			
**GF: Role**	**Placebo**	48	6.3 (0.23)	6.9 (0.24)	-0.12	0.568	0.953	9	5.9 (0.39)	6.8 (0.64)	-0.36	0.474	0.166
	**Fluoxetine**	44	6.6 (0.25)	6.8 (0.26)				12	6.8 (0.41)	6.9 (0.50)			
**AQoL total**	**Placebo**	41	53.3 (1.55)	64.7 (1.87)	-0.29	0.206	0.984	8	59.2 (3.22)	66.0 (4.37)	-0.07	0.897	0.611
	**Fluoxetine**	39	52.4 (2.05)	61.3 (2.05)				11	53.3 (4.49)	63.3 (3.99)			
**BPRS total**	**Placebo**	47	46.3 (1.21)	37.6 (1.32)	0.42	0.051	0.583	9	40.8 (1.88)	35.3 (3.03)	0.34	0.501	0.993
	**Fluoxetine**	43	46.5 (1.36)	41.6 (1.66)				11	44.5 (2.57)	40.0 (3.59)			
**SANS total**	**Placebo**	48	20.1 (1.69)	12.9 (1.50)	0.32	0.127	0.893	9	20.2 (4.15)	9.9 (2.97)	0.42	0.389	0.931
	**Fluoxetine**	44	19.8 (1.84)	15.8 (1.90)				12	15.3 (3.47)	11.4 (2.77)			
**MADRS total**	**Placebo**	48	25.0 (1.35)	15.4 (1.41)	0.34	0.112	0.936	9	19.9 (3.31)	13.2 (3.77)	0.38	0.433	0.438
	**Fluoxetine**	42	25.3 (1.59)	19.0 (1.88)				11	20.8 (3.34)	18.5 (3.90)			
**SOFAS**	**Placebo**	48	56.8 (1.63)	63.9 (1.92)	-0.15	0.491	0.538	9	52.3 (4.18)	62.6 (4.61)	0.10	0.835	0.484
	**Fluoxetine**	44	57.4 (1.86)	62.6 (1.83)				12	58.4 (4.04)	65.9 (4.27)			
**CAARMS, Severity**													
**UTC**	**Placebo**	48	2.7 (0.26)	1.0 (0.24)	0.40	0.058	0.912	9	1.9 (0.56)	0.8 (0.52)	0.06	0.910	0.845
	**Fluoxetine**	44	2.7 (0.25)	1.7 (0.31)				12	2.6 (0.53)	0.8 (0.51)			
**NBI**	**Placebo**	48	3.1 (0.23)	1.3 (0.24)	0.44	0.038	0.020	9	2.7 (0.67)	0.8 (0.36)	1.13	0.027	0.956
	**Fluoxetine**	44	3.1 (0.26)	2.1 (0.31)				12	3.3 (0.57)	2.6 (0.60)			
**PA**	**Placebo**	48	3.2 (0.18)	2.5 (0.25)	0.18	0.402	0.155	9	2.7 (0.58)	2.6 (0.56)	-0.11	0.825	0.161
	**Fluoxetine**	44	3.2 (0.25)	2.8 (0.27)				12	2.3 (0.61)	2.3 (0.54)			
**DS**	**Placebo**	48	1.5 (0.18)	1.3 (0.18)	-0.05	0.819	0.841	9	1.1 (0.39)	1.6 (0.34)	-0.30	0.566	0.807
	**Fluoxetine**	44	1.5 (0.19)	1.3 (0.20)				12	1.8 (0.31)	1.4 (0.34)			
**CAARMS, Frequency**													

© 2023 McGorry PD et al. *JAMA Psychiatry.*

**UTC**	**Placebo**	48	2.4 (0.24)	1.2 (0.26)	0.12	0.571	0.320	9	1.7 (0.47)	0.8 (0.57)	0.09	0.855	0.570
	**Fluoxetine**	43	2.7 (0.25)	1.4 (0.26)				11	2.5 (0.56)	0.8 (0.55)			
**NBI**	**Placebo**	46	3.0 (0.24)	1.6 (0.30)	0.27	0.216	0.228	9	2.1 (0.54)	1.8 (0.74)	0.18	0.727	0.520
	**Fluoxetine**	44	3.3 (0.27)	2.2 (0.32)				12	3.7 (0.60)	2.6 (0.65)			
**PA**	**Placebo**	47	2.7 (0.17)	2.0 (0.22)	0.27	0.204	0.745	8	1.9 (0.48)	2.0 (0.57)	0.05	0.923	0.861
	**Fluoxetine**	43	2.6 (0.25)	2.4 (0.28)				12	1.9 (0.54)	2.0 (0.62)			
**DS**	**Placebo**	48	2.5 (0.28)	2.2 (0.30)	-0.09	0.672	0.953	9	1.9 (0.70)	2.9 (0.66)	-0.44	0.386	0.863
	**Fluoxetine**	44	2.1 (0.28)	2.0 (0.31)				12	3.1 (0.48)	2.4 (0.60)			

AQoL, Assessment of Quality of Life; BPRS, Brief Psychiatric Rating Scale; CAARMS, Comprehensive Assessment of At-Risk Mental States; DS, Disorganized speech; GF: Global Functioning; MADRS, Montgomery-Åsberg Depression Rating Scale; NBI, Non-bizarre ideas; PA, Perceptual abnormalities; SANS, Scale for the Assessment of Negative Symptoms; SOFAS, Social and Occupational Functioning Assessment Scale; UTC, Unusual thought content.
[1] Comparing placebo and fluoxetine using general linear model analysis with baseline score as a covariate.
[2] Interaction between Step 2 treatment (SPS/CBCM) and Step 3 treatment (placebo/fluoxetine) using general linear model analysis with baseline score as a covariate.

© 2023 McGorry PD et al. *JAMA Psychiatry.*

eTable 9. Complete-case and per-protocol analysis of remission rates

		Complete-case			Per-protocol		
		n	% remission[1]	P value	n	% remission[1]	P value
**Step 1**		285	9.5		135	6.7	
**Step 2**	**SPS**	97	14.4	0.878	33	6.1	0.510
	**CBCM**	95	12.6		63	12.7	
**Step 3**	**Placebo**	47	19.1	0.896	8	12.5	0.910
	**Fluoxetine**	44	15.9		12	25.0	

CBCM, cognitive-behavioral case management; SPS, support and problem solving.
P value calculated using the chi-square test.
[1] Refers to sustained remission (i.e., remission criteria were met at weeks 4 *and* 6 for Step 1, 12 *and* 24 for Step 2, and 36 *and* 52 for Step 3).

© 2023 McGorry PD et al. *JAMA Psychiatry.*

eTable 10. Complete-case and per-protocol analysis comparing relapse rates between SPS and monitoring (Step 1 remitters) at 6 and 12 months

		Complete-case analysis			Per-protocol analysis		
		**n**	**% relapsed**	**P value**	**n**	**% relapsed**	**P value**
**6 months**	**Monitoring**	13	7.7	0.023	7	0	0.070
	**SPS**	11	54.5		6	50.0	
**12 months**	**Monitoring**	7	42.9	>0.999	5	20.0	0.524
	**SPS**	8	50.0		5	60.0	

SPS, support and problem solving.
P value calculated using the Fisher's exact test.

© 2023 McGorry PD et al. *JAMA Psychiatry.*

eTable 11. Complete-case and per-protocol analysis comparing relapse rates between SPS and monitoring (Step 2 remitters) at 12 months using logistic regression

	Complete-case analysis				Per-protocol analysis			
	**n**	**% relapsed**	**P value**	**Interaction P value[1]**	**n**	**% relapsed**	**P value**	**Interaction P value[1]**
**Monitoring**	10	40.0	0.797	0.429	8	25.0	0.867	0.879
**SPS**	9	33.3			7	28.6		

SPS, support and problem solving.

[1] Interaction between the factors SPS vs CBCM and monitoring vs SPS.

© 2023 McGorry PD et al. *JAMA Psychiatry.*

eTable 12. Complete-case and per-protocol analysis comparing SPS and monitoring (Step 1 remitters) at 6 months

		Complete-case analysis					Per-protocol analysis				
			Baseline	Month 6				Baseline	Month 6		
		n	Mean (SE)	Mean (SE)	Effect size	P value	n	Mean (SE)	Mean (SE)	Effect size	P value
**GF: Social**	**Monitoring**	12	7.1 (0.26)	7.5 (0.23)	-1.23	0.009	6	7.2 (0.48)	7.5 (0.34)	-1.71	0.017
	**SPS**	11	6.6 (0.36)	6.1 (0.39)			6	6.8 (0.40)	5.8 (0.40)		
**GF: Role**	**Monitoring**	12	6.7 (0.40)	7.0 (0.48)	-0.08	0.844	6	6.8 (0.48)	7.0 (0.52)	-0.06	0.916
	**SPS**	11	6.8 (0.44)	7.0 (0.36)			6	6.7 (0.49)	6.8 (0.48)		
**AQoL total**	**Monitoring**	11	66.8 (2.45)	72.7 (2.68)	-0.18	0.693	6	69.7 (3.19)	72.2 (2.79)	-0.04	0.945
	**SPS**	10	69.9 (4.24)	72.3 (5.67)			6	67.0 (5.52)	68.7 (8.91)		
**BPRS total**	**Monitoring**	12	37.0 (1.58)	34.0 (1.74)	0.04	0.919	6	34.3 (2.08)	33.8 (3.10)	-0.26	0.690
	**SPS**	11	38.5 (2.48)	35.0 (2.69)			6	40.5 (3.47)	37.2 (4.09)		
**SANS total**	**Monitoring**	12	11.4 (1.39)	11.3 (3.55)	-0.15	0.735	6	9.3 (1.67)	10.0 (4.40)	-0.27	0.719
	**SPS**	11	17.7 (3.55)	16.6 (5.44)			6	20.8 (4.50)	17.7 (5.52)		
**MADRS total**	**Monitoring**	12	14.4 (2.56)	8.7 (1.40)	0.55	0.201	6	12.8 (3.75)	10.7 (2.40)	0.30	0.631
	**SPS**	11	16.0 (3.52)	14.1 (3.81)			6	18.0 (5.54)	16.2 (5.80)		
**SOFAS**	**Monitoring**	13	61.9 (3.37)	71.7 (2.46)	-1.06	0.019	7	59.1 (4.96)	70.7 (3.93)	-1.26	0.048
	**SPS**	11	57.7 (3.19)	59.0 (4.11)			6	57.5 (2.95)	55.7 (5.13)		
**CAARMS, Severity**											
**UTC**	**Monitoring**	13	1.1 (0.40)	0.3 (0.13)	0.40	0.338	7	0.7 (0.57)	0.3 (0.18)	0.12	0.849
	**SPS**	11	1.5 (0.62)	0.7 (0.41)			6	2.0 (0.93)	0.7 (0.67)		
**NBI**	**Monitoring**	13	1.4 (0.45)	0.5 (0.29)	0.14	0.732	7	0.4 (0.43)	0.0 (0.00)	0.52	0.448
	**SPS**	11	1.6 (0.51)	0.7 (0.43)			6	2.2 (0.75)	0.7 (0.49)		
**PA**	**Monitoring**	13	2.5 (0.50)	0.6 (0.35)	0.58	0.171	7	2.9 (0.74)	1.1 (0.59)	0.50	0.404
	**SPS**	11	2.4 (0.49)	1.5 (0.56)			6	2.0 (0.68)	2.0 (0.89)		
**DS**	**Monitoring**	13	1.5 (0.37)	1.0 (0.25)	-0.08	0.855	7	1.0 (0.49)	1.1 (0.34)	-0.40	0.501
	**SPS**	11	1.4 (0.43)	0.9 (0.41)			6	1.5 (0.50)	0.7 (0.42)		
**CAARMS, Frequency**											

© 2023 McGorry PD et al. *JAMA Psychiatry.*

**UTC**	**Monitoring**	13	1.2 (0.46)	0.5 (0.27)	0.17	0.689	7	0.6 (0.43)	0.4 (0.30)	-0.51	0.405
	**SPS**	11	1.0 (0.43)	0.7 (0.38)			6	1.5 (0.67)	0.3 (0.33)		
**NBI**	**Monitoring**	13	1.6 (0.53)	0.9 (0.52)	-0.13	0.754	7	0.4 (0.43)	0.0 (0.00)	1.01	0.157
	**SPS**	11	2.0 (0.67)	0.7 (0.38)			6	2.3 (0.84)	0.8 (0.54)		
**PA**	**Monitoring**	13	2.2 (0.39)	0.5 (0.24)	0.86	0.049	7	1.9 (0.55)	0.9 (0.40)	0.70	0.239
	**SPS**	11	2.4 (0.53)	1.8 (0.67)			6	2.3 (0.84)	2.2 (0.98)		
**DS**	**Monitoring**	13	1.7 (0.46)	1.6 (0.38)	0.00	0.993	7	0.9 (0.40)	1.9 (0.51)	-0.36	0.563
	**SPS**	11	2.0 (0.66)	1.6 (0.70)			6	2.0 (0.73)	1.3 (0.88)		

AQoL, Assessment of Quality of Life; BPRS, Brief Psychiatric Rating Scale; CAARMS, Comprehensive Assessment of At-Risk Mental States; DS, Disorganized speech; GF: Global Functioning; MADRS, Montgomery-Åsberg Depression Rating Scale; NBI, Non-bizarre ideas; PA, Perceptual abnormalities; SANS, Scale for the Assessment of Negative Symptoms; SOFAS, Social and Occupational Functioning Assessment Scale; SPS, support and problem solving; UTC, Unusual thought content.
P value: comparing monitoring and SPS using general linear model analysis with baseline score as a covariate.

© 2023 McGorry PD et al. *JAMA Psychiatry.*

eTable 13. Complete-case and per-protocol analysis comparing SPS and monitoring (Step 1 remitters) at 12 months

		Complete-case analysis					Per-protocol analysis				
			Baseline	Month 12				Baseline	Month 12		
		n	Mean (SE)	Mean (SE)	Effect size	P value	n	Mean (SE)	Mean (SE)	Effect size	P value
GF: Social	Monitoring	7	6.9 (0.40)	6.9 (0.40)	-0.21	0.705	5	7.0 (0.55)	7.2 (0.49	-0.78	0.266
	SPS	8	6.3 (0.41)	6.6 (0.42)			5	6.6(0.40)	6.6 (0.51		
GF: Role	Monitoring	7	6.1 (0.51)	7.4 (0.37)	-1.03	0.082	5	6.6 (0.51)	7.6 (0.40	-0.76	0.270
	SPS	8	7.0 (0.53)	7.1 (0.40)			5	6.8(0.58)	7.0 (0.55		
AQoL total	Monitoring	6	69.6 (3.18)	78.4 (3.98)	-0.28	0.612	5	69.5 (3.90)	76.7 (4.44	-0.15	0.824
	SPS	8	71.8 (5.15)	75.7 (6.74)			5	66.4 (6.71)	72.2 (9.59		
BPRS total	Monitoring	7	35.0 (1.85)	32.6 (2.25)	0.12	0.830	5	34.0(2.51)	33.2 (3.14	0.49	0.550
	SPS	8	39.4 (3.09)	35.5 (3.64)			5	42.6 (3.39)	39.0 (4.88		
SANS total	Monitoring	7	11.7 (1.44)	9.3 (3.25)	-0.03	0.957	5	10.4 (1.57)	9.6 (4.63	0.22	0.836
	SPS	8	20.8 (4.14)	15.3 (4.17)			5	24.2 (3.65)	16.6 (4.06		
MADRS total	Monitoring	7	13.0 (4.04)	7.9 (3.01)	0.33	0.546	5	12.8 (4.59)	9.4 (3.92	0.46	0.523
	SPS	8	18.0 (4.42)	12.6 (4.92)			5	21.0 (5.70)	16.4 (7.22		
SOFAS	Monitoring	8	55.9 (3.41)	60.5 (10.43)	0.13	0.801	5	51.8 (1.98)	75.0 (6.89	-0.87	0.248
	SPS	8	57.5 (3.78)	61.8 (5.32)			5	57.0 (3.56)	57.8 (6.55		
CAARMS, Severity											
UTC	Monitoring	8	0.9 (0.52)	0.5 (0.50)	-0.74	0.171	5	0.8 (0.80)	0.8 (0.80)	-1.31	0.098
	SPS	8	1.5 (0.76)	0.0 (0.00)			5	2.4 (1.03)	0.0 (0.00)		
NBI	Monitoring	8	0.8 (0.49)	0.3 (0.25)	0.89	0.121	5	0.0 (0.00)	0.0 (0.00)	2.55	0.038
	SPS	8	1.9 (0.61)	1.5 (0.76)			5	2.6 (0.75)	1.6 (1.03)		
PA	Monitoring	8	2.9 (0.69)	1.8 (0.56)	-0.17	0.740	5	3.2 (0.80)	1.8 (0.80)	-0.25	0.728
	SPS	8	2.0 (0.63)	1.4 (0.71)			5	1.8 (0.80)	1.6 (1.03)		
DS	Monitoring	8	1.4 (0.53)	0.6 (0.32)	0.14	0.779	5	1.0 (0.63)	0.6 (0.40)	-0.24	0.736
	SPS	8	1.6 (0.53)	0.9 (0.52)			5	1.8 (0.49)	0.4 (0.40)		
CAARMS, Frequency											

© 2023 McGorry PD et al. *JAMA Psychiatry.*

**UTC**	**Monitoring**	8	0.9 (0.44)	0.3 (0.25)	-0.62	0.242	5	0.6 (0.60)	0.4 (0.40)	-1.37	0.088
	**SPS**	8	1.1 (0.55)	0.0 (0.00)			5	1.8 (0.73)	0.0 (0.00)		
**NBI**	**Monitoring**	8	1.0 (0.68)	0.6 (0.63)	0.16	0.771	5	0.0 (0.00)	0.0 (0.00)	1.25	0.235
	**SPS**	8	2.5 (0.85)	1.3 (0.65)			5	2.8 (0.86)	1.6 (0.98)		
**PA**	**Monitoring**	7	1.9 (0.51)	1.4 (0.69)	-0.16	0.758	4	1.8 (0.63)	0.8 (0.48)	0.40	0.571
	**SPS**	8	1.9 (0.64)	1.1 (0.64)			5	2.0 (0.95)	1.4 (0.98)		
**DS**	**Monitoring**	8	1.0 (0.38)	0.9 (0.44)	-0.04	0.941	5	0.8 (0.49)	1.0 (0.63)	-0.43	0.582
	**SPS**	8	2.3 (0.80)	1.5 (0.80)			5	2.4 (0.75)	1.0 (1.00)		

AQoL, Assessment of Quality of Life; BPRS, Brief Psychiatric Rating Scale; CAARMS, Comprehensive Assessment of At-Risk Mental States; DS, Disorganized speech; GF: Global Functioning; MADRS, Montgomery-Åsberg Depression Rating Scale; NBI, Non-bizarre ideas; PA, Perceptual abnormalities; SANS, Scale for the Assessment of Negative Symptoms; SOFAS, Social and Occupational Functioning Assessment Scale; SPS, support and problem solving; UTC, Unusual thought content.
P value comparing monitoring and SPS using general linear model analysis with baseline score as a covariate.

© 2023 McGorry PD et al. *JAMA Psychiatry.*

eTable 14. Complete-case and per-protocol analysis comparing SPS and monitoring (Step 2 remitters) at 12 months

		Complete-case analysis							Per-protocol analysis						
			Baseline	Month 12						Baseline	Month 12				
		n	Mean (SE)	Mean (SE)	Effect size	P value[1]	P value[2]		n	Mean (SE)	Mean (SE)	Effect size	P value[1]	P value[2]
**GF: Social**	**Monitoring**	10	7.2 (0.33)	7.4 (0.22)	-0.64	0.207	0.309		8	6.9 (0.30)	7.4 (0.26)	-0.57	0.300	0.399
	**SPS**	9	6.7 (0.24)	6.7 (0.37)					7	6.7 (0.29)	6.7 (0.47)			
**GF: Role**	**Monitoring**	10	6.9 (0.69)	7.1 (0.35)	0.14	0.772	0.335		8	7.0 (0.87)	7.4 (0.32)	0.19	0.722	0.957
	**SPS**	9	7.1 (0.35)	7.4 (0.60)					7	7.0 (0.31)	7.7 (0.68)			
**AQoL total**	**Monitoring**	8	60.8 (2.09)	65.2 (3.05)	-0.31	0.548	0.636		7	60.3 (2.34)	63.9 (3.21)	-0.05	0.927	0.552
	**SPS**	9	63.2 (2.93)	65.5 (4.16)					7	61.8 (2.93)	65.6 (5.05)			
**BPRS total**	**Monitoring**	10	40.5 (2.04)	36.5 (2.66)	0.17	0.718	0.559		8	40.5 (2.58)	34.8 (2.56)	0.67	0.226	0.771
	**SPS**	9	39.4 (1.68)	37.2 (3.41)					7	38.9 (1.39)	38.0 (4.41)			
**SANS total**	**Monitoring**	10	12.0 (3.58)	8.2 (3.10)	0.25	0.598	0.305		8	12.3 (4.47)	4.8 (1.35)	0.79	0.159	0.920
	**SPS**	9	12.0 (2.91)	9.9 (3.22)					7	12.9 (3.31)	11.0 (4.05)			
**MADRS total**	**Monitoring**	10	19.4 (2.46)	14.0 (1.81)	0.57	0.246	0.459		8	19.1 (2.94)	13.3 (2.21)	1.06	0.072	0.949
	**SPS**	9	16.7 (2.30)	17.6 (4.09)					7	16.0 (2.54)	19.7 (4.94)			
**SOFAS**	**Monitoring**	10	62.8 (3.39)	71.3 (2.29)	-0.55	0.255	0.696		8	63.6 (3.52)	72.9 (2.39)	-0.54	0.322	0.691
	**SPS**	9	62.1 (2.24)	66.2 (4.11)					7	63.9 (2.36)	69.3 (4.61)			
**CAARMS, Severity**														
**UTC**	**Monitoring**	10	1.3 (0.50)	0.3 (0.21)	0.52	0.323	0.486		8	1.1 (0.58)	0.3 (0.25)	0.33	0.601	0.976
	**SPS**	9	2.6 (0.48)	1.1 (0.59)					7	2.9 (0.55)	0.9 (0.60)			
**NBI**	**Monitoring**	10	2.5 (0.54)	1.8 (0.59)	-0.04	0.934	0.803		8	2.3 (0.65)	1.5 (0.68)	-0.26	0.633	0.611
	**SPS**	9	2.7 (0.47)	1.8 (0.66)					7	2.7 (0.61)	1.3 (0.68)			
**PA**	**Monitoring**	10	2.9 (0.59)	1.7 (0.68)	0.16	0.740	0.908		8	2.6 (0.68)	1.5 (0.71)	0.37	0.514	0.993
	**SPS**	9	3.4 (0.18)	2.1 (0.72)					7	3.4 (0.20)	2.4 (0.87)			
**DS**	**Monitoring**	10	1.5 (0.34)	0.8 (0.36)	0.39	0.424	0.431		8	1.5 (0.33)	0.8 (0.41)	0.39	0.468	0.600
	**SPS**	9	1.8 (0.40)	1.3 (0.50)					7	1.6 (0.48)	1.3 (0.64)			
**CAARMS, Frequency**														
**UTC**	**Monitoring**	10	1.6 (0.60)	0.4 (0.27)	0.41	0.407	0.328		8	1.1 (0.58)	0.3 (0.25)	0.58	0.321	0.191

© 2023 McGorry PD et al. *JAMA Psychiatry.*

	**SPS**	9	2.3 (0.41)	1.0 (0.67)				7	2.3 (0.52)	1.1 (0.86)			
**NBI**	**Monitoring**	10	2.9 (0.59)	2.1 (0.62)	-0.41	0.411	0.968	8	2.6 (0.68)	1.9 (0.77)	-0.59	0.306	0.773
	**SPS**	8	2.5 (0.50)	1.4 (0.50)				6	2.3 (0.67)	0.8 (0.40)			
**PA**	**Monitoring**	10	2.3 (0.47)	1.2 (0.42)	0.11	0.825	0.642	8	1.9 (0.44)	1.1 (0.44)	-0.19	0.767	0.779
	**SPS**	9	3.4 (0.44)	1.3 (0.55)				7	3.4 (0.48)	1.4 (0.69)			
**DS**	**Monitoring**	10	2.5 (0.65)	1.5 (0.67)	0.16	0.740	0.412	8	2.9 (0.74)	1.3 (0.68)	0.10	0.857	0.186
	**SPS**	9	2.8 (0.64)	1.9 (0.66)				7	2.6 (0.81)	1.4 (0.75)			

AQoL, Assessment of Quality of Life; BPRS, Brief Psychiatric Rating Scale; CAARMS, Comprehensive Assessment of At-Risk Mental States; DS, Disorganized speech; GF: Global Functioning; MADRS, Montgomery-Åsberg Depression Rating Scale; NBI, Non-bizarre ideas; PA, Perceptual abnormalities; SANS, Scale for the Assessment of Negative Symptoms; SOFAS, Social and Occupational Functioning Assessment Scale; SPS, support and problem solving; UTC, Unusual thought content.

[1] Comparing monitoring and SPS using general linear model analysis with baseline score as a covariate.

[2] Interaction between the factors SPS vs CBCM and M vs SPS using general linear model analysis with baseline score as a covariate.

© 2023 McGorry PD et al. *JAMA Psychiatry.*

eTable 15. Kaplan-Meier estimated transition rates (complete-case and per-protocol analysis)

		Complete-case analysis					Per-protocol analysis				
		Cases (n)	Transitions (n)	Estimated transition rate (%)[1]	95% CI	P value	Cases (n)	Transitions (n)	Estimated transition rate (%)[1]	95% CI	P value
**Step 1 non-remitters**	**SPS**	127	2	1.6	0–3.9	0.141	33	0	0	NA	0.323
	**CBCM**	127	6	5.0	1.0–8.9		68	2	2.9	0–6.9	
**Step 1 remitters**	**Monitoring**	14	0	0	NA	0.137	7	0	0	NA	0.273
	**SPS**	13	2	0	NA		6	1	0	NA	
**Step 2 non-remitters**	**Placebo**	57	2	1.8	0–5.3	0.191	9	0	0	NA	0.365
	**Fluoxetine**	56	5	4.0	0–9.2		12	1	0	NA	
**Step 2 remitters**	**Monitoring**	14	0	0	NA	NA	12	0	0	NA	NA
	**SPS**	11	0	0	NA		7	0	0	NA	

CBCM, cognitive-behavioral case management; SPS, support and problem solving.
[1] 6-month transition rates for Step 1 non-remitters; 12-month rates for all other steps.
P value comparing the survival curves of the treatments concerned using log-rank test.

© 2023 McGorry PD et al. *JAMA Psychiatry.*

eTable 16. Treatment adherence rates

		n	% adherent	P value
**Step 1**	**SPS**	342	41.2	
Non-remitter treatment arm				
**Step 2**	**SPS**	127	26.0	<0.001
	**CBCM**	127	53.5	
**Step 3**	**CBCM**	113	46.9	
**Step 3**	**Fluoxetine**	56	26.8	>0.99
	**Placebo**	57	26.3	
Remitter treatment arm				
**Step 2**	**Monitoring**	14	50.0	>0.99
	**SPS**	13	46.2	
**Step 3**	**Monitoring**	14	85.7	0.35
	**SPS**	11	63.6	

SPS, support and problem solving.
P value calculated using the Fisher's exact test.

© 2023 McGorry PD et al. *JAMA Psychiatry.*

eTable 17. Adverse events

	Adverse events (n)					Serious adverse events (n)		
	None known	Mild	Mod	Severe	P value	None known	Yes	P value
***Step 2 (non-remitters from Step 1)*[1]**								
**SPS**	125	0	1	1	>0.99	126	1	>0.99
**CBCM**	125	1	0	1		126	1	
***Step 3 (remitters from Step 2)*[2]**								
**Monitoring (SPS)[4]**	9	0	0	0	0.64	9	0	-
**SPS (SPS)**	5	0	0	0		5	0	
**Monitoring (CBCM)**	5	0	0	0		5	0	
**SPS (CBCM)**	5	1	0	0		6	0	
***Step 3 (non-remitters from Step 2)*[3]**								
**Fluoxetine (SPS)**	26	2	0	1	0.94	28	1	0.61
**Placebo (SPS)**	24	2	1	0		27	0	
**Fluoxetine (CBCM)**	24	2	0	1		26	1	
**Placebo (CBCM)**	28	1	1	0		30	0	

Adverse and serious adverse events recorded: [1] Step 2 (non-remitters from Step 1): headache and sleep disturbance (n=1), erectile dysfunction (n=1), and overdose with suicidal intent (n=2). For those who entered Step 2 as remitters from Step 1, no known adverse events occurred. [2] Step 3 (remitters from Step 2): headache (n=1). [3] Step 3 (non-remitters from Step 2): headache (n=3; 2 of these were serious adverse events that required hospital admission), sleep disturbance (n=1), nausea (n=4), headache and nausea (n=1), nausea, fatigue and perceptual abnormality (n=1), and diarrhea (n=1).

[4] The treatment listed in brackets denotes the one received in the prior step: (SPS) = support and problem solving received in Step 2; (CBCM) = cognitive-behavioral case management received in Step 2.

P value calculated using the Fisher's exact test.

© 2023 McGorry PD et al. *JAMA Psychiatry.*

eFigure 1. Changes in mean symptom and functioning scores from baseline to the end of Step 2. Circles represent individual participant data.

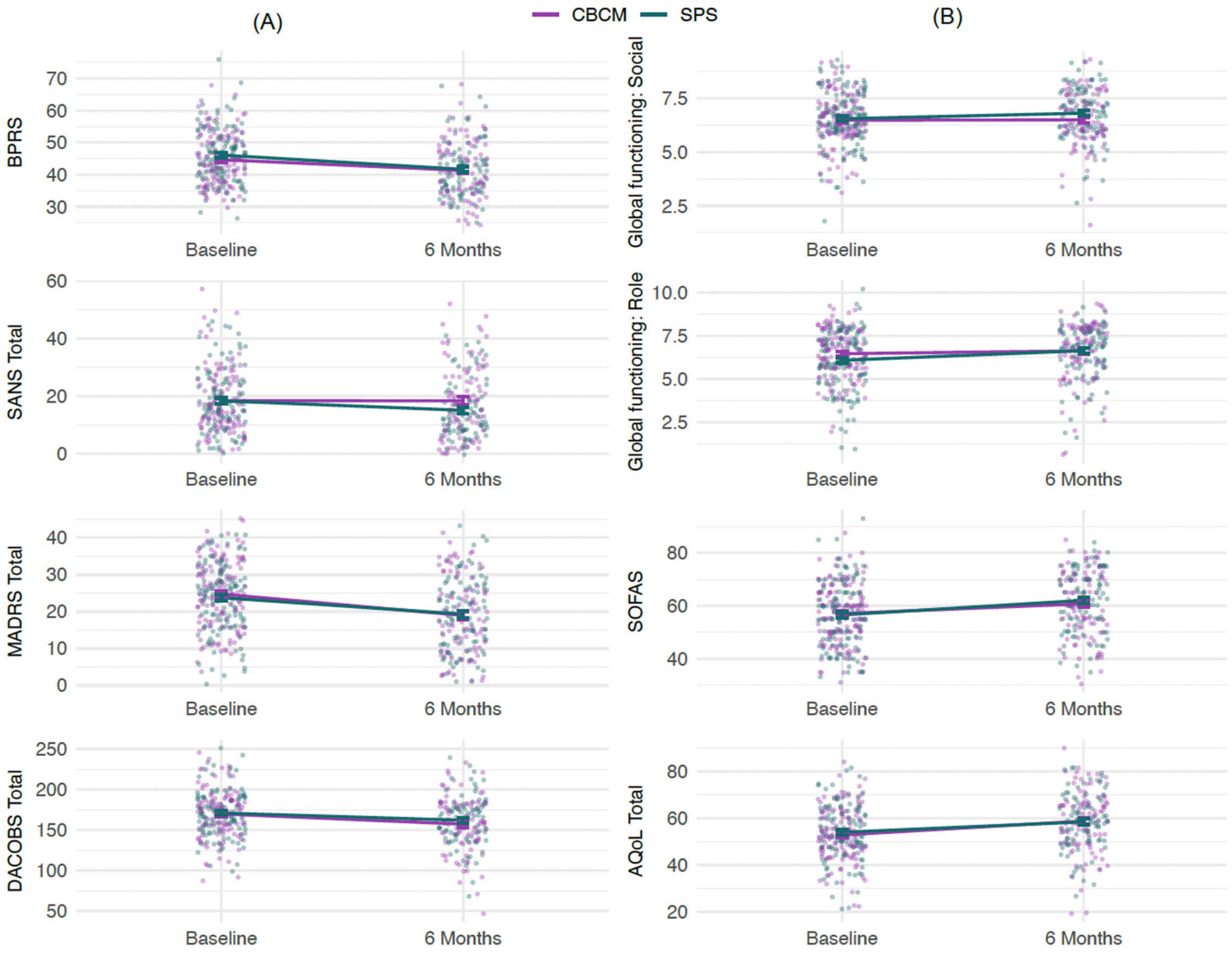

© 2023 McGorry PD et al. *JAMA Psychiatry.*

eFigure 2. Changes in mean CAARMS severity scores from baseline to the end of Step 2 (panel A) and Step 3 (panel B). Circles represent individual participant data. Fluoxetine/placebo commenced at 6 months (start of Step 3).

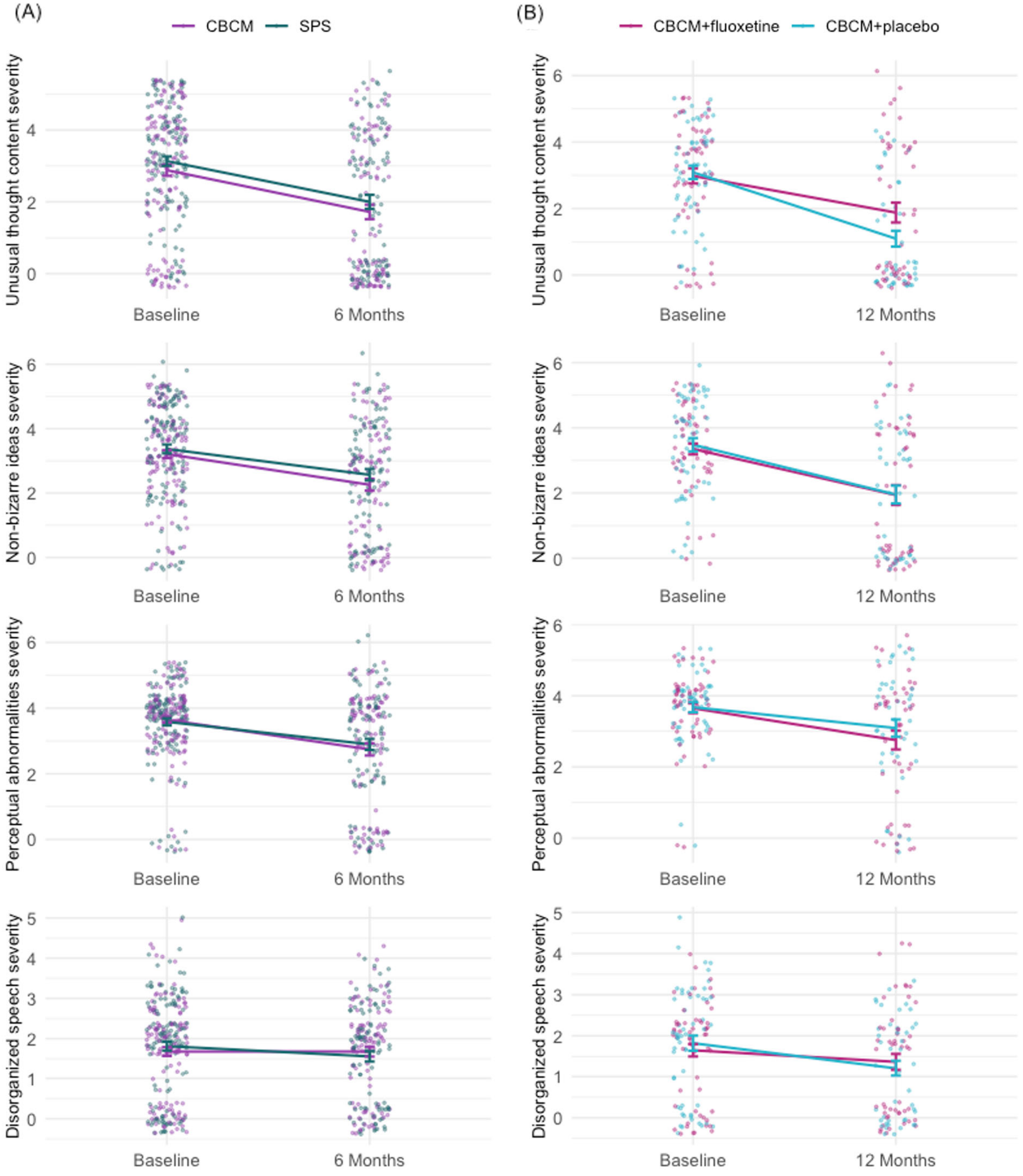

© 2023 McGorry PD et al. *JAMA Psychiatry.*

eFigure 3. Changes in mean CAARMS frequency scores from baseline to the end of Step 2 (panel A) and Step 3 (panel B). Circles represent individual participant data. Fluoxetine/placebo commenced at 6 months (start of Step 3).

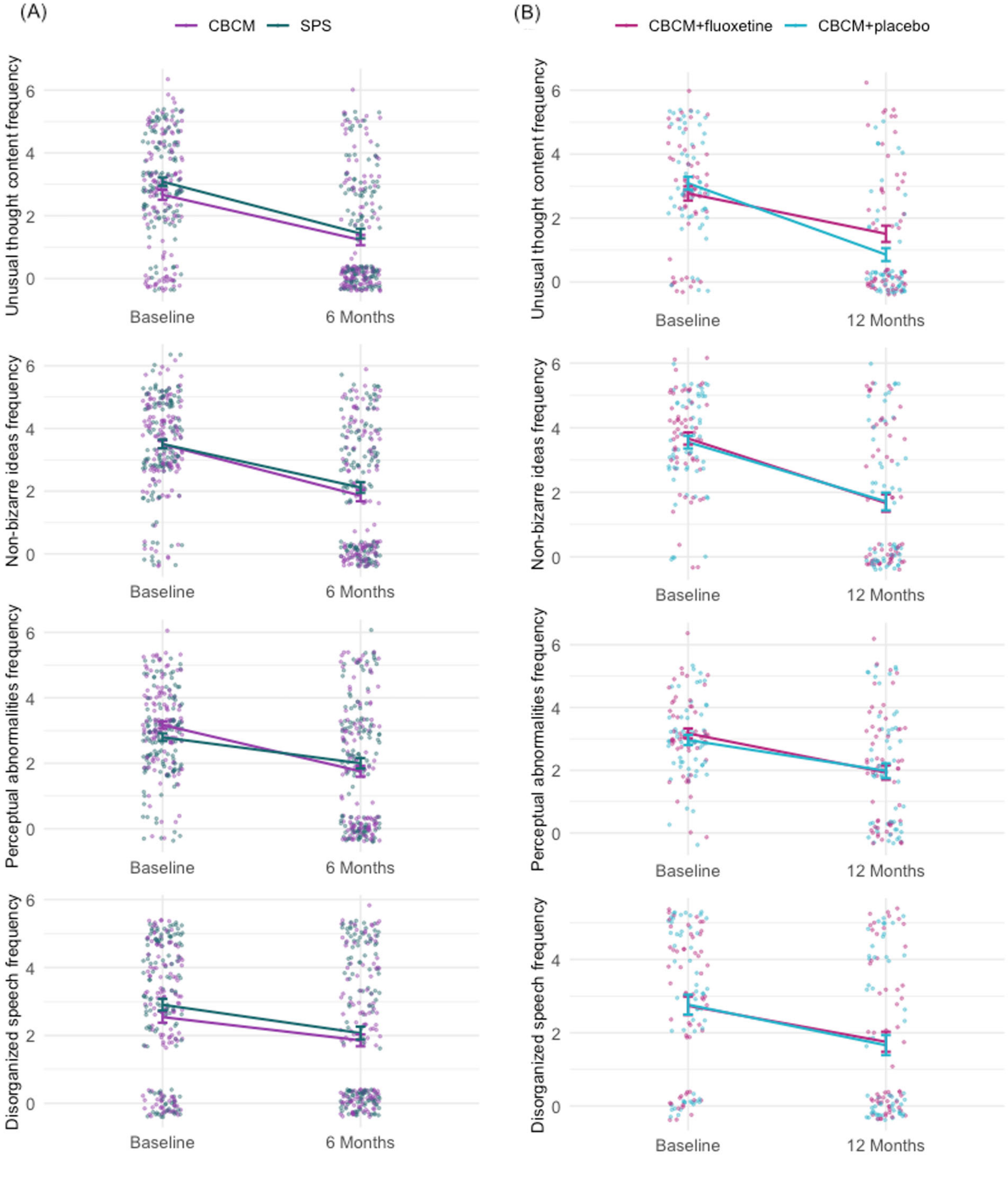

© 2023 McGorry PD et al. *JAMA Psychiatry.*
