# Supplementary material for: A Sequential Adaptive Intervention Strategy Targeting Remission and Functional Recovery in Young People at Ultrahigh Risk of Psychosis: The Staged Treatment in Early Psychosis (STEP) Sequential Multiple Assignment Randomized Trial
Source: JAMA Psychiatry. 2023 Jun 28;80(9):875–85. doi: 10.1001/jamapsychiatry.2023.1947 (PMC10308298; doi:10.1001/jamapsychiatry.2023.1947)
Supplement: Supplement 3. — Data sharing statement [file jamapsychiatry-e231947-s003.pdf]

Data Sharing Statement

McGorry. A Sequential Adaptive Intervention Strategy Targeting Remission and Functional Recovery in Young People at Ultrahigh Risk of Psychosis. *JAMA Psychiatry*. Published June 28, 2023. doi:10.1001/jamapsychiatry.2023.1947

Data
Data available: Yes
Data types: Deidentified participant data, Data dictionary
How to access data: The data that support the findings of this study are available from the corresponding author (pat.mcgorry@orygen.org.au) upon reasonable request.
When available: With publication

Supporting Documents
Document types: None

Additional Information
Who can access the data: Data will be shared with researchers whose proposed use of the data has been approved by an independent review committee.
Types of analyses: Data will be made available for analyses that achieve the aims in the approved protocol.
Mechanisms of data availability: Signed data access agreement.
